# Control of Cellular Differentiation Trajectories for Cancer Reversion

**DOI:** 10.1002/advs.202402132

**Published:** 2024-12-11

**Authors:** Jeong‐Ryeol Gong, Chun‐Kyung Lee, Hoon‐Min Kim, Juhee Kim, Jaeog Jeon, Sunmin Park, Kwang‐Hyun Cho

**Affiliations:** ^1^ Department of Bio and Brain Engineering Korea Advanced Institute of Science and Technology Daejeon 34141 Republic of Korea

**Keywords:** Boolean gene regulatory network model, cancer reversion, cell fate control, network reconstruction, single‐cell transcriptome, systems biology

## Abstract

Cellular differentiation is controlled by intricate layers of gene regulation, involving the modulation of gene expression by various transcriptional regulators. Due to the complexity of gene regulation, identifying master regulators across the differentiation trajectory has been a longstanding challenge. To tackle this problem, a computational framework, single‐cell Boolean network inference and control (BENEIN), is presented. Applying BENEIN to human large intestinal single‐cell transcriptome data, MYB, HDAC2, and FOXA2 are identified as the master regulators whose inhibition induces enterocyte differentiation. It is found that simultaneous knockdown of these master regulators can revert colorectal cancer cells into normal‐like enterocytes by synergistically inducing differentiation and suppressing malignancy, which is validated by in vitro and in vivo experiments.

## Graphical abstract

A computational framework, single‐cell Boolean network inference and control (BENEIN), is presented. Applying BENEIN to human large intestinal single‐cell transcriptome, MYB, HDAC2, and FOXA2 are identified as the master regulators whose inhibition induces enterocyte differentiation. It is found that knockdown of these master regulators can revert colorectal cancer cells into normal‐like enterocytes by synergistically inducing differentiation and suppressing malignancy.

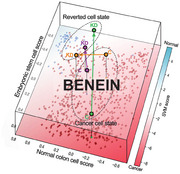

## 1 Introduction

Cancer reversion has been proposed as a new therapeutic approach that aims to revert cancer cells into their differentiated and non‐malignant state,^[^ ^1^, ^2^, ^3^, ^4^, ^5^ ^]^ by inducing re‐expression of differentiation associated genes.^[^ ^6^, ^7^, ^8^ ^]^ Interestingly, in acute myeloid leukemia, breast cancer, and hepatocellular carcinoma, it was found that differentiation or trans‐differentiation of cancer cells can induce such reversion.^[^ ^6^, ^7^, ^8^ ^]^ However, systematic identification of master regulators that induce differentiation/trans‐differentiation remains elusive. If master regulators across normal differentiation processes can be identified and utilized to regulate cancer cells, they may constitute an alternative approach to overcome the limitations of current anti‐cancer therapies.

Despite the importance of identifying master regulators of cellular differentiation, it remains a challenging problem due to the complex and strongly nonlinear nature of gene regulation.^[^ ^9^ ^]^ Hence, there is a pressing need to develop a computational framework to identify master regulators that encompass dynamic processes in cellular differentiation. Although the dynamics of Boolean networks may appear overly simplistic in contrast to the intricate nature of biological systems, they still represent the essential features of biological mechanisms, making Boolean network modeling an appropriate approach.^[^ ^10^, ^11^ ^]^ In previous studies, Boolean network modeling of cellular differentiation was proposed by constructing the structure of gene regulatory networks (GRNs) based on correlation coefficients, performing pseudotime analysis, and using Boolean satisfiability (SAT) solvers to infer Boolean logics.^[^ ^12^ ^]^ However, such studies have shown problems including limited scalability, incompleteness in elucidating specific structural information, and the assumption of irregular time point intervals in inferring the regulation logics of Boolean network models (Table S1, Supporting Information). To overcome these limitations, we develop a computational framework for single‐cell Boolean network inference and control (BENEIN).

BENEIN can reconstruct Boolean models of GRNs and identify a set of master regulators, whose regulation leads to the desired cellular differentiation.^[^ ^13^, ^14^ ^]^ In particular, BENEIN splits the transcriptional status of each single‐cell into pre‐ and post‐transition states using the exonic and intronic information of transcripts and infers the regulation logic of the underlying GRNs by assuming that the two states before and after the state transition correspond to exonic and intronic expression levels, which also remains unbiased with respect to uneven cell clusters upon the pseudotime trajectory. BENEIN further employs complex network control to identify the master regulators that can induce the desired cellular differentiation. BENEIN reveals insight into hidden gene regulation dynamics and offers a systemic way of controlling them.

Applying BENEIN to single‐cell transcriptome data of adult human intestine,^[^ ^15^ ^]^ we identified a combination of master regulators, consisting of MYB, HDAC2, and FOXA2, which play a critical role in blocking enterocyte differentiation. We examined the regulation effects of these control targets by in silico analysis of the reconstructed GRN model, as well as comparative analysis with various public transcriptome data. To further confirm the effect of cancer reversion, we simultaneously inhibited these targets in three colorectal cancer cell lines and xenograft mouse models and found that their combinatorial inhibition strongly induces differentiation into normal‐like cells. This demonstrates the potential for BENEIN to reveal novel control targets for differentiation trajectories in cancer reversion.

In addition to applying BENEIN to adult human intestine single‐cell transcriptome data, we explored its utility in a different organism and cellular context. We applied BENEIN to the granule neuron differentiation in the developing mouse hippocampus. Through this process, we identified a combination of control targets: Tcf4^[^ ^16^ ^]^ (overexpression), Klf9^[^ ^17^ ^]^ (overexpression), and Etv4^[^ ^18^ ^]^ (inhibition). These targets are known to play pivotal roles in granular cell differentiation, as validated by literature. This application of BENEIN highlights the capability of BENEIN not only in reconstructing Boolean GRN models but also in identifying control targets for control of the cellular differentiation trajectories in diverse contexts. These findings suggest that BENEIN is a powerful tool for identifying control targets that are potentially pivotal in cancer reversion and other biological processes.

## 2 Result

### 2.1 Overview of the BENEIN Workflow

**Figure 1:**
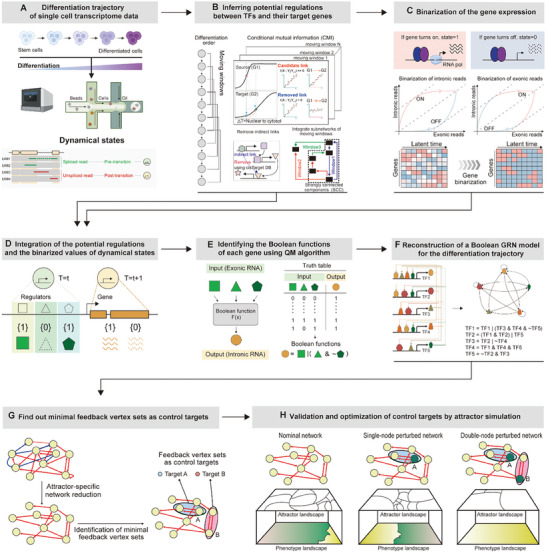
Schematic diagram of BENEIN for reconstruction of Boolean GRN from single‐cell transcriptome data and identification of master regulators for cancer reversion. A) BENEIN quantifies the abundance of pre‐mature and mature mRNA reads to separate the transcriptional status of each single‐cell into pre‐ and post‐transition states. B) BENEIN infers a potential regulatory structure between TFs and their TGs with a moving window strategy, by computing CMI and then using cisTarget database for eliminating indirect interactions for each window. C) BENEIN converts gene expressions of the pre‐ and post‐transition states into the binarized forms, where a value of 1 indicates that the gene is switched on and a value of 0 indicates that the gene is switched off. D–F) BENEIN infers Boolean functions for each gene by integrating the binarized matrices with the regulatory network structure to construct truth tables, then employing the QM algorithm. By iterating the process, BENEIN reconstructs the Boolean GRN model. G) BENEIN reduces the Boolean GRN model to its essential structure and finds minimal feedback vertex sets of the reduced network model to identify control target candidates. H) The identified control targets are optimized and validated by attractor simulation.

To regulate cellular differentiation trajectories for cancer reversion, we developed a computational framework, BENEIN. BENEIN utilizes single‐cell transcriptome data across a differentiation process and quantifies the abundance of pre‐mature and mature mRNA reads. This quantification allows the transcriptional status of each single‐cell to be separated into two dynamical states: pre‐ and post‐transition states (**Figure** **1**A). To reconstruct an accurate Boolean model of the GRN based on these pre‐ and post‐transition states, BENEIN first infers potential regulatory structures between transcription factors (TFs) and their target genes (TGs). To uncover temporal gene regulatory interactions during the differentiation process, BENEIN groups cells into several clusters along with the differentiation trajectory and infers a structure within each cluster by computing conditional mutual information (CMI)^[^ ^19^ ^]^ and eliminating indirect interactions between TFs and their TGs using the cisTarget database.^[^ ^20^ ^]^ BENEIN integrates these structures across the first half clusters and establishes the regulatory network structure. Since the dynamics of the GRN are dominantly driven by TFs with complex feedback structures,^[^ ^21^, ^22^, ^23^ ^]^ BENEIN extracts the largest strongly connected component (SCC) from the regulatory network structure (Figure 1B).

A Boolean network model is based on binarized ON/OFF values, which requires that gene expression of pre‐ and post‐transition states are binarized. BENEIN achieves this by identifying switching points on a phase plot of intronic and exonic reads for each gene, and binarizing the intronic and exonic gene expression based on these switching points. As a result, BENEIN generates two binarized matrices for the exonic and intronic states in a gene‐by‐cell configuration. The binarized values for the exonic reads represent the presence (ON) or absence (OFF) of the TFs, whereas those for the intronic reads indicate whether the genes are being transcribed (ON) or not (OFF) (Figure 1C).

By integrating the binarized matrices with the regulatory network structure, BENEIN generates a truth table and converts the truth table into a Boolean function by employing the Quine‐McCluskey (QM) algorithm.^[^ ^24^ ^]^ By iterating this process for all the genes in the network structure, BENEIN reconstructs the Boolean GRN model that can represent the regulatory mechanisms underlying cellular differentiation trajectories (Figure 1D–F).

To identify the master regulators of the Boolean GRN model, we employed the BNSimpleReduction algorithm^[^ ^25^ ^]^ and identified the minimal feedback vertex set (FVS)^[^ ^26^, ^27^ ^]^ which is a set of vertices (genes) in a directed graph that will make the graph acyclic when removed. The BNSimpleReduction algorithm reduces a Boolean GRN while maintaining the dynamical information for the desired state. BENEIN applies the FVS control algorithm to the reduced Boolean GRN model to identify master regulators that can induce differentiation into a desired state (Figure 1G,H) (Further details of BENEIN are provided in the Experimental Section).

### 2.2 Reconstruction of a Boolean GRN Model for Human Colon Enterocyte Differentiation Using BENEIN

**Figure 2:**
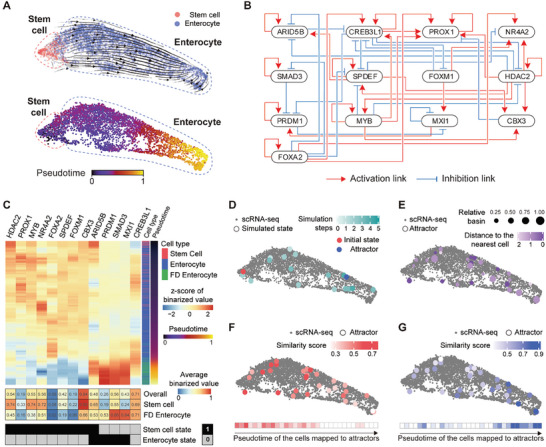
Inference and analysis of the Boolean GRN model for enterocyte differentiation. A) Single‐cell transcriptome data of enterocyte differentiation upon the UMAP embedding space with velocity stream plot (top) and pseudotime (bottom). B) The Boolean GRN model for enterocyte differentiation constructed by BENEIN. Red and blue edges represent activation and repression, respectively. C) Heatmap illustrating the gene expression dynamics along with the pseudotime. Gene expressions were binarized based on RNA splicing dynamics calculated by scVelo, then smoothed and converted to *z*‐score for improved visualization. Based on the gene expression dynamics, the binarized states for stem cell and enterocyte were determined by comparing the average gene activation in the cluster of each cell type and the average gene activation of all the cells in the trajectory. D) State transitions of the Boolean GRN model from a stem cell initial state (red) to an enterocyte attractor (blue) mapped onto the UMAP embedding space (see the Experimental Section for details on determining the position of each network state upon the UMAP embedding space). E) 32 point attractors of the Boolean GRN model mapped onto the UMAP embedding space, with the relative basin of attraction and Hamming distance between each attractor and binarized expression of the nearest cell. Similarity scores of the attractors with respect to F) the stem cell state and G) the enterocyte state upon the UMAP embedding space. The similarity score is defined by one minus the normalized Hamming distance between an attractor and the binarized state of the cell type.

We applied BENEIN to intestinal single‐cell transcriptome data to examine its usefulness in inferring the Boolean GRN model and controlling the differentiation process based on that model. Among various human intestinal differentiation processes, we focused on the enterocyte lineage since enterocytes play major functions in the large intestine, such as water absorption. For this purpose, we collected publicly available single‐cell transcriptome data^[^ ^15^ ^]^ of normal human colon and rectum samples and identified 4252 single‐cells undergoing enterocyte differentiation (**Figures** **2**A and Figure S1, Supporting Information).

By applying BENEIN to the single‐cell data, we inferred a GRN structure of 522 genes and 1841 interactions, and extracted the largest SCC including 17 TFs and 93 regulatory interactions. We finally obtained an executable Boolean GRN model consisting of 13 TFs and 46 interactions (Figure 2B). Detailed regulatory logics of the Boolean GRN model and supporting evidence obtained from literature or databases are provided in Table S2 (Supporting Information). Despite the purely data‐driven inference of the framework, about 60% of the interactions inferred by BENEIN coincide with the supporting evidence (Table S2, Supporting Information). In addition, the general structural properties of the GRNs are present in the reconstructed Boolean GRN model by BENEIN (Note S1, Supporting Information).^[^ ^28^ ^]^

### 2.3 Boolean GRN Model Inferred by BENEIN Reflects the Dynamics of the Original Single‐Cell Data

To examine whether BENEIN can infer a Boolean GRN model that properly represents the dynamics of a given single‐cell transcriptome data, we analyzed the dynamic properties of the reconstructed Boolean GRN model of human colon enterocyte differentiation. To compare the gene expression dynamics of the single‐cell data and the dynamics of the Boolean GRN model, we first determined the binarized states for each cell type by analyzing the gene expression dynamics over the differentiation trajectory. Intestinal stem cells and fully differentiated (FD) enterocyte cells were annotated by LGR5 and KRT20 expression, respectively. The binarized states for each cell type were then determined by computing whether a gene is activated or repressed in the cluster of each cell type compared to the average of all the cells in the differentiation trajectory (Figure 2C). The FD enterocytes exhibit high differentiation related signatures and low proliferation related signatures. In addition, we performed analyses with various cutoffs of KRT20 expression and clustering parameters to ensure the robustness of the FD enterocyte state. We showed that the FD enterocyte state is robustly defined across various criteria for determining FD enterocytes (Figure S2, Supporting Information).

We investigated the state transitions of the Boolean GRN model to examine how well they represent the differentiation trajectory. This investigation was carried out by performing a state transition simulation, starting from an initial state that represents the stem cell state, in order to obtain a simulated trajectory of differentiation. It turns out that the simulated trajectory from the initial state toward a steady state in the enterocyte cluster closely resembles the differentiation trajectory observed in the single‐cell transcriptome data (Figure 2D). This similarity indicates that the Boolean GRN model can provide a reasonably accurate representation of the differentiation trajectory through its state transitions. To investigate the steady state properties of the Boolean GRN model, we performed further simulation analysis and explored the attractor landscape, which is a set of stable states known as attractors that a system eventually reaches, along with the basin of attraction for each attractor. It is known that these attractors are associated with particular cell phenotypes.^[^ ^29^ ^]^ The attractor landscape of the Boolean GRN model consists of 12 cyclic attractors and 36 point attractors. By mapping the point attractors onto the uniform manifold approximation and projection (UMAP) embedding space,^[^ ^30^ ^]^ we found that these point attractors are well spread over the single‐cell transcriptome data, and that our GRN model can represent the overall features of the data (Figure 2E).

To examine the characteristics of the steady states in the Boolean GRN model, we defined the similarity score of each steady state based on a Hamming distance between the binarized state of each cell type and the steady states. By projecting the similarity scores of each point attractor on the UMAP embedding space, we observed a trend in the similarity score to the stem cell/enterocyte state decreasing/increasing over pseudotime (Figure 2F,G). This trend aligns with the conventional understanding of stem cell differentiation toward enterocytes. A detailed comparison between the gene expression patterns of the single‐cell transcriptome data and the dynamics of the Boolean GRN model is provided in Figure S3 (Supporting Information). We also assessed capability of BENEIN to predict the regulation logic of downstream genes by incorporating the logic of TGs controlled by the key TFs in the constructed Boolean GRN model. Although these TGs do not alter the dynamics of the GRN model since they do not further impact downstream elements, we built a TG‐inclusive GRN model consisting of 37 nodes. This model also effectively captures the key regulatory dynamics of the differentiation trajectory (Figure S4, Supporting Information). Overall, our findings suggest that BENEIN accurately inferred the Boolean GRN model of human colon enterocyte differentiation, incorporating both transient and steady state properties.

### 2.4 Identification of Optimal Control Targets for Induction of Enterocyte Differentiation

**Figure 3:**
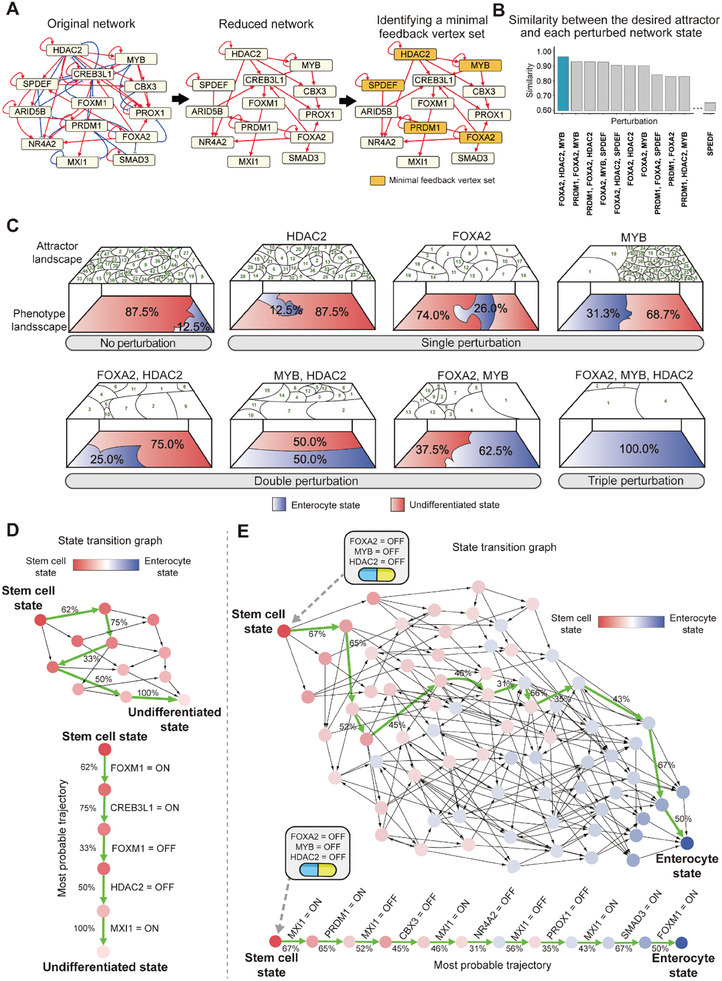
Identification of optimal control targets for enterocyte differentiation and numerical simulations for the effects of control targets on the Boolean GRN model. A) Identification of control target candidates. The original network is shown with red edges representing activation and blue edges representing inhibition (left). The reduced network was obtained by applying the BNSimpleReduction algorithm to the original network (middle). The minimal FVS consisting of 5 nodes (HDAC2, MYB, SPDEF, PRDM1, and FOXA2) is marked in yellow in the reduced network (right). B) Optimization of the control targets. The bar plot shows cosine similarity between the desired attractor and the average activity of each perturbed network. The simultaneous inhibition of MYB, HDAC2, and FOXA2 yielded the highest similarity (≈0.97) and were chosen as the optimal control targets. C) Attractor and phenotype landscape of the unperturbed and each perturbed Boolean GRN model. All possible combinations within the optimal targets are considered. Numbers on the attractor landscape indicate indices of the attractors, and the area indicates a basin of attraction for each attractor. Red/blue in the phenotype landscape indicates a basin of attraction of the undifferentiated/differentiated enterocyte state, respectively. The perturbation corresponding to simultaneous inhibition of optimal control targets resulted in a 100% basin of attraction for enterocyte phenotype states. D,E) State transition trajectories between unperturbed and controlled networks. The graph on the top illustrates all possible state transitions, with the most probable trajectory highlighted in green, along with the transition probability. The graph below illustrates the most probable trajectory with updated node states according to the state transition. Color coding of the state ranges from red (stem cell state) to blue (enterocyte state). Without perturbation, the colon stem cell state can transition toward an undifferentiated state through 13 possible trajectories (D). When the control targets were regulated, the colon stem cell state would transition toward the enterocyte state through 2412 possible trajectories (E).

To identify potential control targets for inducing the differentiation of enterocytes, we employed the BNSimpleReduction algorithm to reduce the reconstructed Boolean GRN model while preserving the essential dynamics leading to the desired state.^[^ ^25^ ^]^ In this study, the desired state was chosen to be the attractor with the highest enterocyte score. We then applied the FVS control algorithm to the reduced Boolean GRN to ensure that the controlled Boolean GRN converges to a desired state regardless of the initial states.^[^ ^26^, ^27^ ^]^ As a result, we identified HDAC2, MYB, SPDEF, PRDM1, and FOXA2 as potential control targets (**Figure** **3**A).

**Figure 4:**
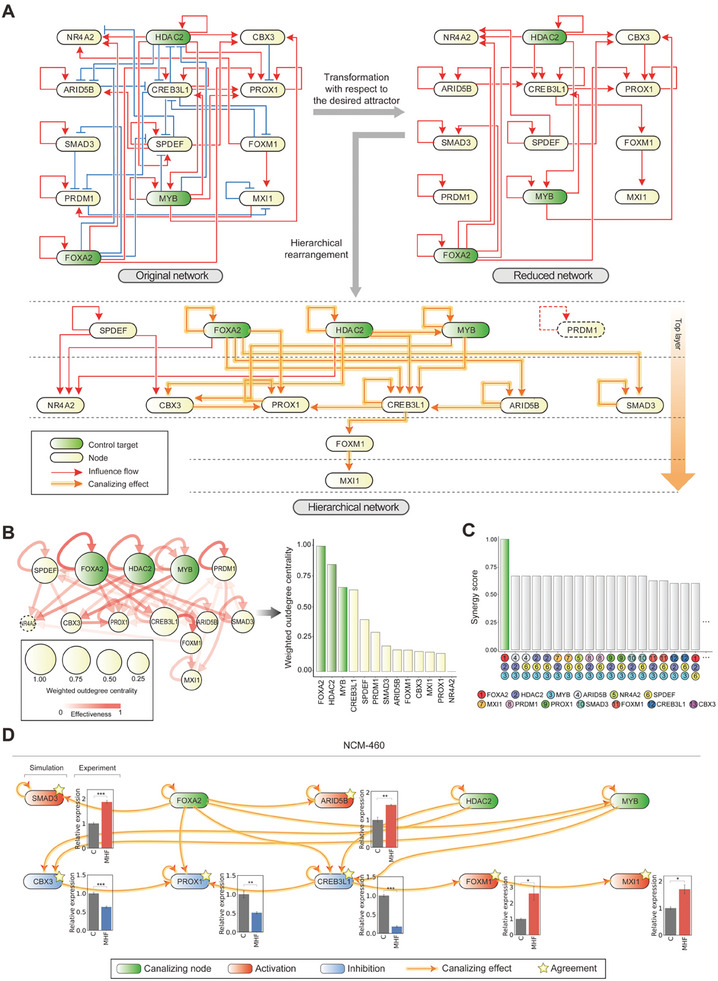
The underlying dynamical regulation mechanism of enterocyte differentiation induced by regulating the control targets of the Boolean GRN model. A) The transformation of the original network into a reduced network and its further rearrangement into a hierarchical network. The original network is shown with red edges representing activation and blue edges representing repression (top left). The reduced network was obtained by applying the BNSimpleReduction algorithm (top right). The reduced network is rearranged to visualize its hierarchical structure. Control targets are colored green in each of the networks, and their canalizing effects on the downstream nodes are highlighted in yellow on the edges (bottom). B) The Boolean GRN model visualized with the effectiveness (edges) and the weighted outdegree centrality (nodes). Color coding of each edge represents the effectiveness. Size of the node represents the weighted outdegree centrality, computed using effectiveness as weights (left). Bar plot showing the weighted outdegree of the effectiveness of each node, in descending order (right). The control targets show the highest weighted outdegree. C) Bar plot showing the synergy score for every possible three‐node perturbation. The nodes are each symbolized with a unique number and color. The combination of optimal control targets shows the highest synergy score. D) Comparison of the canalizing effect from the simultaneous perturbation of MYB, HDAC2, and FOXA2 in the Boolean GRN model with the in vitro transcript quantification on NCM‐460. The network is a subnetwork of the reduced network from Figure 4A, consisting of the canalized nodes. Nodes are colored red (activation) or blue (inhibition) according to the canalization effect. The in vitro transcript quantification results are shown in bar charts, with a scramble knockdown sample colored in gray and triple knockdown samples colored in red (up‐regulated) or blue (down‐regulated). Data are presented as the mean  ± SEM; *n* = 3 measurements (two‐tailed *t*‐test: *p < 0.05, **p < 0.01, ***p < 0.001) (NCM‐460 with scramble knockdown, C; NCM‐460 with simultaneous knockdown of MYB, HDAC2, and FOXA2, MHF).

To reduce the number of control targets while ensuring sufficient control efficacy, we examined all possible combinations of the potential control targets by performing attractor simulations. For each combination, we computed average activity vectors and cosine similarities between them and the desired state (Table S3, Supporting Information). As a result, simultaneous inhibition of MYB, HDAC2, and FOXA2 exhibited the highest similarity of ≈0.97 (Figure 3B). We also performed 268 perturbation simulations involving all possible combinations of three nodes within the network, including knockdown and overexpression, and confirmed that the simultaneous inhibition of MYB, HDAC2, and FOXA2 appears to be the most effective (Note S1, Supporting Information). To further investigate the significance of the three control targets for normal intestinal stem cell maintenance, we utilized scTenifoldKnK^[^ ^31^ ^]^ for the virtual knockdown of these targets. As a result, each control target was found to be essential in maintaining intestinal stem cell characteristics. Furthermore, those targets were significantly linked to the Wnt pathway, known to play a vital role in maintaining normal intestinal stem cells^[^ ^32^ ^]^ (**Figure** **4**A). Together, simultaneous inhibition of MYB, HDAC2, and FOXA2 were identified as optimal control targets for enterocyte differentiation.

### 2.5 Analysis of Attractor Landscapes of Controlled GRNs

To explore the effect of regulating the identified optimal control targets on the Boolean GRN model,^[^ ^33^ ^]^ we analyzed the phenotype and attractor landscapes for all partial combinations of the optimal control targets by performing attractor simulations^[^ ^34^ ^]^ (Table S4, Supporting Information). Here, the phenotype landscape refers to a map of phenotypes defined by the stable states of specific output molecules within the attractor landscape. Given that the four attractors resulting from the simultaneous perturbation of MYB, HDAC2, and FOXA2 are located within the fully differentiated enterocyte cluster, they can be considered as enterocyte phenotype states (Figure S5, Supporting Information).

The unperturbed network has 48 attractors and the proportion of the basin of attraction corresponding to the enterocyte phenotype state is only about 12.5%. As the number of perturbations increased, the proportion of enterocyte phenotype states in the phenotype landscape increased, while the number of attractors decreased (Figure 3C). When all the control targets, MYB, HDAC2, and FOXA2, were perturbed, the proportion of the basin of attraction corresponding to the enterocyte phenotype state became 100% (although there is a difference of 0.03 in the similarity between the average activity vector of the four attractors resulting from the simultaneous perturbation of all the control targets and the desired state, these attractors still corresponded to the enterocyte phenotype from a phenotypic perspective).

To compare state transition trajectories of the unperturbed and perturbed networks in response to regulation of control targets, we chose an initial state representing a colon stem cell and conducted state transition simulations from the initial state. Without perturbation, the colon stem cell state transitioned to an undifferentiated state through 13 trajectories with general asynchronous state update^[^ ^35^ ^]^ (Figure 3D). When the control targets were perturbed in the Boolean GRN, we observed transitions from the colon stem cell state into the enterocyte state through 2412 trajectories, which confirms that regulating the control targets is sufficient to drive differentiation in our model (Figure 3E).

### 2.6 Mechanistic Understanding of the Controlled Enterocyte Differentiation

Both structure and logic of the Boolean GRN determine the state transition dynamics of Boolean networks.^[^ ^10^, ^36^ ^]^ To elucidate the underlying mechanisms by which regulation of the control targets induces enterocyte differentiation, we conducted an analysis of the structure of the reduced Boolean GRN. Interestingly, we found that the reduced Boolean GRN does not include any feedback loops that were present in the original Boolean GRN model, resulting in a tree‐shaped network. By hierarchically rearranging the reduced network, we found that MYB, HDAC2, and FOXA2 are located at the top of the hierarchy, which indicates that they can exert the greatest influence on the GRN and thereby serve as direct driving factors for downstream genes, eventually leading to the enterocyte state (Figure 4A).

We further investigated the logic of the Boolean GRN model to analyze the regulation effects of MYB, HDAC2, and FOXA2 by computing and comparing the effectiveness of all links. Since the effectiveness quantifies the canalizing effect, higher values of the effectiveness indicate a higher probability that the affected node will be canalized.^[^ ^37^ ^]^ We computed the weighted out‐degree centrality of each node using the effectiveness as link weights, where the weighted out‐degree centrality represents the influence of nodes in the Boolean GRN (Table S5, Supporting Information). As a result, we found that the control targets, MYB, HDAC2, and FOXA2, have the highest weighted out‐degree centrality (Figure 4B). To further improve our understanding of such node‐specific influences, we employed a random forest regression model to calculate the importance of each node and found that MYB, HDAC2, and FOXA2 are the most influential (Note S1, Supporting Information). Moreover, we further investigated the TGs of the 13 TFs in the Boolean GRN model to understand differentiation of intestinal stem cells. In particular, regulons of the activated TFs are associated with the differentiation of intestinal enterocytes, while those of the inhibited TFs are associated with cancer cell and stem cell properties (Figure S6, Supporting Information).

To investigate whether the control targets have a synergistic effect, we defined a synergy score by dividing the number of canalized nodes by an additive score for all combinations of the control targets (Table S6, Supporting Information) (Further details on the definition and computation of the synergy score are provided in the experimental section). The combination of MYB, HDAC2, and FOXA2 showed a remarkably high synergistic effect, while the other combinations had either no or relatively small synergistic effects (Figure 4C and Figure S7, Supporting Information).

Simulation analysis shows that simultaneous regulation of MYB, HDAC2, and FOXA2 canalizes the CBX3, PROX1, CREB3L1, ARID5B, SMAD3, FOXM1, and MXI1 nodes. To investigate whether these canalizing effects of MYB, HDAC2, and FOXA2 are consistent with experimental results in normal cells, we performed in vitro transcript quantification of the canalized genes in the NCM‐460 colon normal cell line. This normal cell line was chosen as the representative non‐cancerous colon cell. We established the perturbed NCM‐460 cells with double (MYB+HDAC2, MH; MYB+FOXA2, MF; HDAC2+FOXA2, HF) and triple knockdowns (MYB+HDAC2+FOXA2, MHF), then measured mRNA expression levels of the canalized genes. The simulation results were consistent with the experimental results in the NCM‐460 cells with simultaneous MYB, HDAC2, and FOXA2 knockdown (Figure 4D). Concurrent analysis on HT‐29, HCT‐116, and CACO‐2 is provided in Figures S8–S10 (Supporting Information) and Table S7 (Supporting Information).

Together, our analysis demonstrates that the simultaneous perturbation of MYB, HDAC2, and FOXA2 has the most pronounced influence on controlling the Boolean GRN model toward the desired state and that the states of the downstream nodes canalized by the control of MYB, HDAC2, and FOXA2 in simulation analysis largely agree with the results of in vitro knockdown experiments on the colorectal cancer cells. Therefore, MYB, HDAC2, and FOXA2 are master regulators in controlling the Boolean GRN model toward an enterocyte state and could be effective targets for cancer treatment.

### 2.7 In Silico Analysis Reveals That Inhibition of the Master Regulators Has Potential for Cancer Reversion

Based on the in silico knockout analysis of the control targets in normal intestinal stem cells using scTenifoldKnK,^[^ ^31^ ^]^ we could infer that the three control targets are associated with colon cancer signatures (Figure S11A, Supporting Information). Furthermore, from LINCS L1000 data analysis, we could also infer that knockout of each control target is related to the differentiation of colon cancer. These suggest that inhibition of each control target is highly relevant to repressing colon cancer malignancy (Figure S11B, Supporting Information). In addition, we performed in silico knockout of each control target in pre‐cancerous cells from colon cancer and matched normal single‐cell RNA‐seq data of Zheng et al. We also found the control targets can induce reversion in benign tumor cells and pre‐cancerous cells as well as cancer cells (Figures S12 and S13, Supporting Information).

### 2.8 Simultaneous Knockdown of the Master Regulators Leads to Suppressed Proliferation of Three Colorectal Cancer Cells In Vitro and In Vivo

**Figure 5:**
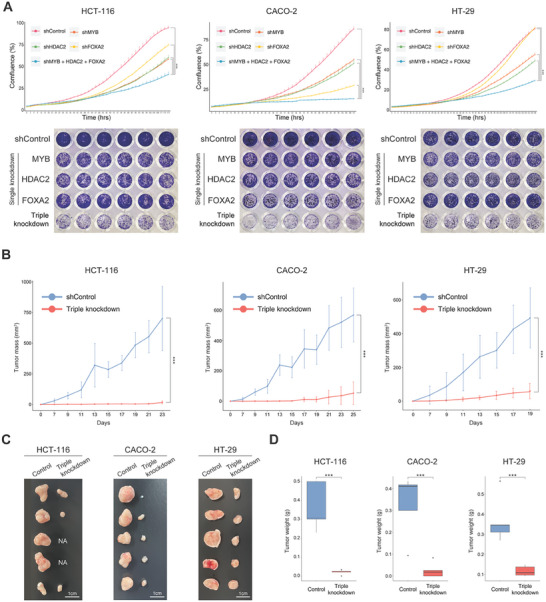
The simultaneous perturbation of control targets inhibited proliferation of three colorectal cancer cells in vitro and in vivo. A) The growth curves of colorectal cancer cells (HCT‐116, CACO‐2, and HT‐29) after knockdown of control targets (top). Cell growth rate was analyzed by IncuCyte. Representative images of crystal violet staining of cells (bottom). Data are presented as the mean ± SEM; *n* = 3 replicates (two tailed *t*‐test: **p < 0.01; ***p < 0.001). B–D) HCT‐116, CACO‐2, and HT‐29 were injected to female athymic nude mice (Foxn1^nu/nu^) and proliferation of cancer cells was observed in tumor‐bearing mice. Changes in the volume of three colorectal tumors 23 days after tumor injection (B). Photographs of tumors resected after sacrifice on day 23 (C). Tumor weight of a resected tumor (D). Data are presented as the mean ± SEM; *n* = 5 measurements (two tailed *t*‐test: ***p < 0.001).

Based on in silico analyses, we knocked down the master regulators in the colorectal cancer cells and measured their proliferation rate to examine whether the cancer cells can be reverted into normal‐like cells. Notably, the proliferation rates of the colorectal cancer cells with simultaneous knockdown were dramatically decreased relative to those of single knockdown (**Figure** **5**A; Figures S14 and S15, Supporting Information). Our in vitro results demonstrated that the simultaneous knockdown of the master regulators reverts three colorectal cancer cells into a normal‐like differentiated state resembling enterocytes with reduced proliferation. To examine whether the knockdown of the master regulators also suppresses proliferation in vivo, we established three control colorectal cancer cell‐engraftment nude mouse models and three simultaneously knocked‐down colorectal cancer cell‐engraftment nude mouse models. Notably, the volume and weight of HT‐29, HCT‐116, and CACO‐2 engrafted tumors with simultaneous knockdown of the master regulators were significantly reduced compared to those of the three control cells (Figure 5B–D). To examine whether simultaneous knockdown of the master regulators effectively reduces proliferation of colorectal cancer cells more than the cells with a single knockdown in vivo, we also established three single knocked‐down HT‐29 cancer cell‐engraftment nude mouse models. The volume of HT‐29 engrafted tumors with simultaneous knockdown of the master regulators was significantly reduced compared to those with the single master regulator knockdown (Figure S16, Supporting Information). These findings suggest that simultaneous knockdown of the master regulators could be a promising therapeutic approach for the treatment of colorectal cancer.

### 2.9 Control of the Master Regulators Leads to Reversion of Colorectal Cancer Cells into Differentiated Normal‐Like Enterocytes

**Figure 6:**
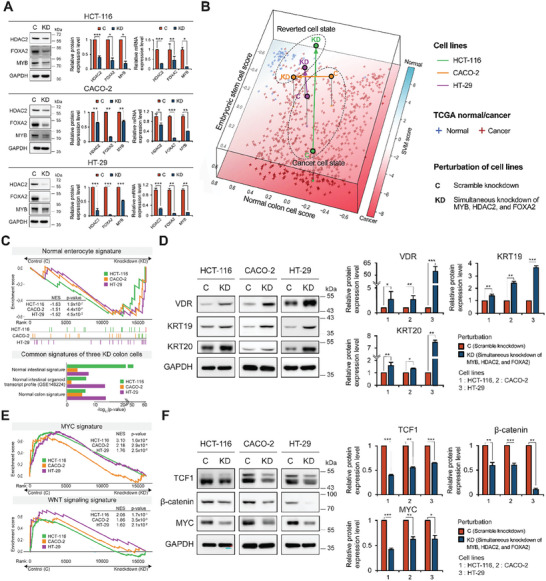
Suppression of control targets reverts three colorectal cancer cells into normal‐like enterocytes by inactivating MYC and WNT pathways. A) Protein and mRNA expression levels of MYB, HDAC2, and FOXA2 in HCT‐116, CACO‐2, and HT‐29 cells (Three colorectal cancer cells with scramble knockdown, C; three colorectal cancer cells with simultaneous MYB, HDAC2, and FOXA2 knockdown, KD). B) Scatter plot on the upper layer shows the transcriptome data from three colorectal cancer cells and their respective reverted cells in three colors (Green, HCT‐116; Orange, CACO‐2; Purple, HT‐29). Scatter plot on the lower layer shows the transcriptome data from TCGA colorectal cancer (red) and their adjacent normal (blue), along with the gradient color of the background representing a support vector machine score for TCGA colon cancer expression. The *x*‐ and *y*‐axes represent normal colon cell and embryonic stem cell signature scores, respectively. C) Enrichment plot shows that the normalized enrichment scores (NESs) of normal enterocyte signature were increased by simultaneous knockdown of the control targets for three colon cancer cells (top). Box plot displays results of gene ontology analysis using genes up‐regulated by the simultaneous knockdown of the control targets. The up‐regulated genes are associated with the normal colon signatures (bottom). D) Protein abundances were monitored by western blotting analysis of the representative genes of colonic enterocytes (KRT19, KRT20, and VDR). E) Enrichment plots illustrate that the NESs of MYC (top) and WNT (bottom) signatures were decreased by the simultaneous knockdown of the control targets for three colon cancer cells. F) Protein abundances were monitored by western blotting analysis of the representative genes of MYC and WNT pathways (TCF1, MYC, and β‐catenin). Transcript quantification by qRT‐PCR is presented relative to that in the scramble shRNA condition. GAPDH was used as a loading control. Data are presented as the mean ± SEM; *n* ≥ 3 measurements (two‐tailed *t*‐test: *p < 0.05, **p < 0.01, ***p < 0.001).

After the influence of knocking down MYB, HDAC2, and FOXA2 on the proliferation of cancer cells was validated by in vitro and in vivo experiments, we further investigated whether the simultaneous perturbation leads to reinstating the global gene expression of the three colorectal cancer cells into that of normal enterocyte cells. We measured transcriptome data of three colorectal cancer cells with scramble knockdown (C) and simultaneous knockdown of MYB, HDAC2, and FOXA2 (KD). By comparing the transcriptome data from our three colorectal cancer cells with those of colorectal cancer and adjacent normal samples from The Cancer Genome Atlas (TCGA),^[^ ^38^ ^]^ we confirmed that simultaneous perturbation of the master regulators can revert the three colorectal cancer cell states to the states resembling normal enterocytes. Interestingly, despite the transcriptomic heterogeneity of the three colorectal cancer cells and diverse trajectories of cancer reversion according to the perturbation, the reverted cells exhibit a strikingly similar transcriptomic state, analogous with the transcriptome data of TCGA adjacent normal colorectal tissues (**Figure** **6**B). Normal colon and rectum gene signature scores are also increased when the master regulators are simultaneously controlled (Figure 6A,C). In addition, we validated the normal colorectal features of the reverted colorectal cancer cells by Western blot analysis at the protein level. KRT19, KRT20, and VDR,^[^ ^39^ ^]^ which are markers of colonic enterocytes, were increased in reverted colorectal cancer cells (Figure 6D; Figure S17B, Supporting Information).

We further investigated the underlying mechanism of reversion achieved by perturbation of master regulators. We found that MYC and WNT associated signature scores of the reverted colorectal cancer cells (KD) were lower than those of the colorectal cancer cells (C) (Figure 6A,E). We also validated the decrease of TCF1, MYC, and β‐catenin in the reverted colorectal cancer cells by western blot analysis (Figure 6F; Figure S17C, Supporting Information).

We also conducted overexpression of master regulators independently in three colon cancer cells (HCT‐116, CACO‐2, and HT‐29) and investigated expression changes of cancer‐related genes and markers of colonic enterocytes. We found that the overexpression of the master regulators does not induce either more a cancerous state or a normal‐like reverted state in colon cancer cells. We also conducted the overexpression of the master regulators in NCM‐460 cell line and did not observe increased proliferation. However, protein expression levels of TCF1 and MYC were relatively elevated (Figure S18, Supporting Information). Together, these results indicate that simultaneous knockdown of MYB, HDAC2, and FOXA2 can induce the reversion of colorectal cancer cells.

### 2.10 Application of BENEIN to Mouse Granular Cell Differentiation Shows the Generality and Flexibility of BENEIN

To examine the generality of BENEIN in a different organism and context, we applied BENEIN to single‐cell transcriptome data from a developing mouse hippocampus, specifically focusing on granular cell differentiation (Note S2, Supporting Information). We performed scVelo analysis of granular cell differentiation trajectory consisting of neural intermediate progenitor cells, neuroblast, immature granule, and granule cell types to calculate the RNA velocity and pseudotime. Then, we reconstructed a Boolean GRN model of granular cell differentiation using BENEIN, comprising 27 nodes and 123 links. We investigated the attractor landscape of the Boolean GRN model and found that the Boolean GRN model properly represents the dynamics of granular cell differentiation.

By designating the attractor representing the fully differentiated granular cell as the desired attractor, we identified a set of control targets for inducing granular cell differentiation using BENEIN, Tcf4 (overexpression), Klf9 (overexpression), and Etv4 (inhibition). Each of these control targets has been previously reported as a key regulator in the differentiation of granular cells.^[^ ^16^, ^17^, ^18^ ^]^ Details of the application of BENEIN to mouse granular cell differentiation are provided in Note S2 (Supporting Information). This application demonstrates the capability of BENEIN to reconstruct Boolean GRN models as well as its proficiency in identifying viable control targets for a given single‐cell transcriptome data.

### 2.11 Benchmarking BENEIN with SCENIC and VIPER in Master Regulator Identification for T Cell Development and CD8 T Cell Activation

To further evaluate the capability of BENEIN in reconstructing Boolean GRN models and identifying master regulators, we applied it to single‐cell RNA‐seq data from T cell development^[^ ^40^ ^]^ and CD8 T cell activation.^[^ ^41^ ^]^

For the T cell development trajectory, spanning from early T precursor (ETP) to double negative 3 (DN3) cells, a Boolean GRN model consisting of 15 nodes was reconstructed using BENEIN. This model effectively captured the key transition dynamics from ETP to DN3 cells. Moreover, BENEIN identified Gata3 overexpression as the most efficient control target to promote T cell development. Similarly, for the T cell activation trajectory, from naive to effector cells, a Boolean GRN model with five nodes was reconstructed, and BENEIN identified Eomes overexpression as a key regulatory target for driving the activation process.

To benchmark the performance of BENEIN, we compared it with SCENIC^[^ ^42^ ^]^ and VIPER^[^ ^43^ ^]^ by applying them to the same datasets. SCENIC and VIPER identified regulons specifically activated in DN3 and effector cells. However, a comparison of the top‐ranked genes revealed that BENEIN identified more biologically plausible targets, consistent with known regulatory factors, whereas the other tools, relying primarily on statistical correlations, often identified less relevant or unlikely targets. These results demonstrate the robustness of BENEIN in reconstructing GRN models and its proficiency in identifying biologically more meaningful control targets. Detailed analyses of these applications have been provided in Note S2 (Supporting Information).

## 3 Discussion

In this study, we presented a computational framework, BENEIN, to reconstruct a Boolean GRN and identify master regulators for desired differentiation on the basis of single‐cell transcriptomic data. BENEIN has three distinct features. First, BENEIN utilizes information from exonic and intronic transcripts to split the transcriptional status of each single‐cell into two dynamical states. These dynamical states can be regarded as the pre‐ and post‐transition states according to the underlying gene regulation logic, enabling inference of more accurate regulation logic (Note S1, Supporting Information). In contrast, existing algorithms utilize pseudotime analysis with uneven time intervals between cells, leading to inaccurate regulatory logics. Second, the minimal hyperparameter design of BENEIN facilitates tailored model reconstruction specific to datasets, by simplifying the complicated process of hyperparameter tuning. While existing algorithms^[^ ^40^, ^44^ ^]^ require prior knowledge to reconstruct Boolean GRN models, BENEIN automatically reconstructs Boolean GRN models in an unbiased manner and without such prior knowledge. Therefore, BENEIN effectively incorporates molecular regulation logic into the Boolean GRN model. Lastly, BENEIN is the first attempt to systematically integrate identification of master regulators based on complex network control and inference of an optimal Boolean GRN model, resulting in a complete framework to identify master regulators from single‐cell transcriptomic data. Although various methods of inferring logical regulatory network models for GRNs from single‐cell transcriptome data have been suggested, no single framework has been suggested^[^ ^12^, ^45^, ^46^, ^47^ ^]^ for systemically identifying master regulators for the desired differentiation trajectory on the basis of dynamical analysis of GRNs.

BENEIN is designed to be highly modular, and each module can be easily replaced with another up‐to‐date algorithm module. For instance, although scVelo was employed for binarization of the dynamical states and pseudotime analysis in this study, it can be replaced with other velocity inference algorithms based on deep learning such as DeepVelo^[^ ^48^ ^]^ or cellDancer.^[^ ^49^ ^]^ In addition, other control strategies like “divide and conquer” for global stabilization (DCGS) framework^[^ ^50^ ^]^ or the stable motif control algorithm^[^ ^51^ ^]^ can also be employed for global stabilization.

Previous studies^[^ ^6^, ^8^, ^52^ ^]^ have demonstrated that controlling master regulators for differentiation or trans‐differentiation in cancer cells can lead to reverted or trans‐differentiated cell states. For instance, three TFs (FOXA3, HNF1A, and HNF4A) were shown to induce trans‐differentiation of fibroblasts into hepatocytes.^[^ ^53^ ^]^ Cheng et al. applied these factors to hepatocarcinoma cells to revert them into normal‐like functional hepatocytes.^[^ ^8^ ^]^ However, they lacked a systemic method for identifying such master regulators and also a mechanistic understanding of how regulation of those factors can induce such reversion or trans‐differentiation. To overcome this limitation, BENEIN was developed to systematically investigate the intestinal differentiation process through Boolean GRN analysis and identify the master regulators required for differentiation into enterocytes. We found that the states of the nodes canalized by the simultaneous perturbation of MYB, HDAC2, and FOXA2 in the Boolean GRN model were similar to the results from the in vitro knockdown experiments on the various colorectal cancer cell lines. We also discovered that perturbation of these master regulators can revert colon cancer cells into normal‐like cells. The reverted colon cancer cells exhibited enterocyte‐specific marker gene expressions and limited proliferation compared to original colon cancer cells in vitro and in vivo. Furthermore, we conducted a mechanism analysis of the GRN by applying attractor‐specific network reduction, resulting in an essential network topology associated with the desired state. This transformed the original Boolean GRN into a tree‐shaped backbone network structure. MYB, HDAC2, and FOXA2 were found to be at the top of the hierarchy, implying their pivotal function in driving canalization for the desired enterocyte state.

Extending the utility of BENEIN beyond the human intestinal differentiation context, we applied it to single‐cell transcriptome data from a developing mouse hippocampus, focusing on the differentiation of granular cells. For inducing granular cell differentiation, we identified a combination of control targets using BENEIN: Tcf4 (overexpression), Klf9 (overexpression), and Etv4 (inhibition). Each identified control target had been previously recognized as a key regulator in the differentiation of granular cells. Such cross‐contextual applications underscore the versatility of BENEIN not only in Boolean GRN model reconstruction but also in its ability to identify potential control targets across various differentiation processes.

BENEIN has several advantages for constructing Boolean GRN models and identifying master regulators by analyzing the model. It can robustly construct Boolean GRN models under uncertain noise such as isoform changes due to its capability of robust Boolean function inference (Figures S19 and S20, Supporting Information). Moreover, BENEIN was analyzed as the most effective tool compared to other techniques for identifying master regulators of enterocyte differentiation, T cell development, and CD8 T cell activation (Tables S3, S5 and Figures S5–S9, Note S2, Supporting Information). However, there are several potential limitations in BENEIN's application to cancer reversion. The accumulation of mutations alters the logic of gene expression regulation during tumorigenesis. Therefore, the Boolean GRN model constructed by BENEIN, which simulates the normal differentiation, is limited in its ability to predict the differentiation state of cancer cells. In spite of this limitation, since the ability of normal cells to differentiate can be retained in cancer cells, the cancer reversion targets identified by BENEIN can induce cancer cells to differentiate and to revert into their normal‐like state. We also need to note that alternative splicing events may influence the accuracy of identifying TFs. In our case study, this effect was not significant since the affected TFs were output nodes and thereby the overall regulatory dynamics were not changed (Figure S8, Note S1, Supporting Information), but the effect of alternative splicing may in general significantly influence the inference of GRN and investigation to minimize such effects remains as a further study.

BENEIN can identify the master regulators of a differentiation process through attractor analysis of the reconstructed Boolean GRN model. However, there are potential limitations in its application to cancer reversion. The accumulation of mutations can alter the logic of gene expression regulation during tumorigenesis. Therefore, the Boolean GRN model constructed by BENEIN, which simulates the normal differentiation, is limited in its ability to identify reversion targets and predict the resulting differentiation state of cancer cells. In the example shown in this study, we found that the primary canalizing effect induced by controlling the combination targets identified from normal colon cell differentiation still holds for cancer cells in spite of some discrepancies between simulation and experiments, but this cannot be always guaranteed in other kinds of cancer. Such translation can be effective only if the genetic alteration of cancer cells does not influence the primary canalizing mechanism of differentiation induction.

In this study, we applied BENEIN to intestinal differentiation and identified master regulators that can revert colorectal cancer cells. Since single‐cell transcriptomic data of differentiation trajectories of many other human tissues are becoming more available, BENEIN can be further utilized to identify master regulators of pan‐tissue differentiation^[^ ^54^, ^55^ ^]^ as well as reversion of pan‐cancer cells. BENEIN provides a systemic approach to identify master regulators of cell differentiation and reprogramming and opens a novel route to further investigate the role of such regulators for mechanism‐based therapeutic strategies.

## 4 Experimental Section

### Preprocessing of the Single‐Cell RNA Sequencing Data of Human Colon and Rectum Samples

The single‐cell transcriptome sequencing data of the human colon and rectum were obtained from the Gene Expression Omnibus (GEO) with the accession number GSE125970.^[^ ^15^ ^]^ The acquired sequencing data include two samples from the colon and two from the rectum. Each of these samples was aligned using the Cell Ranger count and aggr pipelines (v6.0.1) with the hg38 reference genome, resulting in a total of 4472 cells from the colon and 3898 cells from the rectum. The Seurat R package (v3.2) was used for quality control (QC) and integration of the count matrix for each sample. While conducting the standard pre‐processing workflow, initial QC was performed with the following criteria: RNA feature count between 200 and 4500, total RNA count less than 10000, and less than 15% mitochondrial gene expression. FindIntegrationAnchors and IntegrateData functions were then run using default parameters to integrate the four samples, resulting in a total of 7283 single‐cell expression data. Dimension reduction using UMAP was performed using 10 PC (principal component) dimensions and other parameters set to default. Unsupervised clustering was performed using FindNeighbors and FindClusters functions in Seurat, with parameters set to 20 PC dimensions and a resolution of 0.5 to identify 24 clusters. Each cluster was then annotated based on the expression of known marker genes such as enterocyte cells (ALPI, SLC26A3, TMEM37, and FABP2), goblet cells (ZG16, CLCA1, FFAR4, TFF3, and SPINK4), Paneth cells (LYZ [Lyz1 and Lyz2 in mouse], CA7, SPIB, CA4, and FKBP1A), enteroendocrine cells (CHGA, CHGB, CPE, NEUROD1, and PYY), progenitor cells (SOX9, CDK6, MUC4, FABP5, PLA2G2A, and LCN2), transient‐amplifying (TA) cells (KI67, PCNA, TOP2A, CCNA2, and MCM5), and stem cells (LGR5, RGMB, SMOC2, and ASCL2).^[^ ^15^ ^]^ Finally, 4252 cells corresponding to stem cells and enterocytes were selected for Boolean GRN model construction.

### Identification of Enterocytes, Benign Tumor Cells, Precancer Cells, and Cancer Cells among Epithelial Cell Subtypes

To investigate the tumorigenesis of colorectal cancer, enterocytes, benign tumor cells, precancerous cells, and cancer cells were classified from single‐cell transcriptome data of normal colon tissues, adenoma tissues, and carcinoma tissues. For this purpose, both epithelial pathological genetic markers and canonical colorectal epithelial markers were employed^[^ ^56^, ^57^ ^]^ as follows: malignant cells: MMP7, LYZ, ERO1A, and BST2; pre‐cancerous cells: TUBA1B, H2AFZ, HMGB2, and HIST1H4C; benign cells: SMOC2; and enterocytes: GUCA2A, GUCA2B, CA4, and CA2.

### scVelo Analysis of the Single‐Cell RNA Sequencing Data

After the preprocessing, RNA velocity analysis was performed on the enterocyte differentiation. The velocyto run10× pipeline (v0.17.15) was applied to the output from the Cell Ranger count pipeline of each sample, yielding loom files containing counts of exonic and intronic reads. These loom files were merged, and the filter_and_normalize and moments functions of scVelo (v0.2.2)^[^ ^58^ ^]^ were performed to preprocess for velocity analysis. Subsequently, a dynamical modeling mode was employed to calculate parameters of significant transcriptional dynamics, including transcription rate, splicing rate, and degradation rate, across 684 genes.

### Inference of Gene–Gene Interaction Backbone Network Structure Using Single‐Cell Data

The backbone network structure used for Boolean logic inference was determined using CMI and a moving window strategy. Initially, the 4252 cells involved in the enterocyte differentiation process were divided into 20 windows, with 400 cells each. For each of these windows, the following steps were undertaken to construct a network structure: First, using the 684 genes with significant velocity and known human TFs, the CMI between TF‐TF or TF‐TG was calculated. The CMI was computed using the cmi function from Scribe (v0.1),^[^ ^19^ ^]^ which, unlike mutual information that only considers the expression of two genes, contemplates the short‐term changes in the target gene calculated through velocity analysis to construct a directed network. Subsequently, a motif enrichment analysis was performed using the ctx step of pySCENIC (v0.10.3).^[^ ^20^ ^]^ This process employed two sections of the cisTarget DB v9 database: hg19‐500bp‐upstream‐7species and hg19‐tss‐centered‐10kb‐7species.^[^ ^20^ ^]^ Through these steps, a network structure for each window was constructed. The link lists from the first half windows were then merged, preserving the maximum importance for each edge to form a backbone network structure comprising 522 genes and 1841 interactions. Finally, a core network structure, the SCC, consisting of 17 TFs and their 93 interactions, was extracted for regulation logic inference.

### Binarization of Intronic and Exonic Reads of Single‐Cell Data

As a result of the scVelo analysis, each gene's parameters for the splicing kinetic model, as hypothesized by scVelo, were estimated. This model visualizes splicing dynamics in a rugby ball shape on a phase plot where exonic and intronic reads are the *x* and *y* axes, respectively. From these dynamics, the point where the exonic read increases/decreases faster than the intronic read was considered to be the current dynamical state of the cell, denoted as ON/OFF. This represents a state where the exonic read is rapidly generated or degraded according to the quantity of the intronic read. Furthermore, the point where the intronic read monotonically increases/decreases was considered to be the cell's next dynamical state, also denoted as ON/OFF. This represents a state where the intronic read is either being produced or not. This process was repeated for all genes where scVelo successfully inferred transcriptional dynamics, determining two binarized dynamical states for each cell.

### Boolean Regulation Logic Inference Using Quine‐McClusky Algorithm

Using the network structure and binarized dynamical states, a pseudo‐truth table can be constructed for each gene for which regulation logic is to be inferred. This pseudo‐truth table is organized with the target gene as output and the genes regulating the target gene as inputs. Each row of the pseudo‐truth table is filled with the next state of the target gene and the current states of the regulators depending on the dynamical state of each cell. Since the pseudo‐truth table is filled based on the dynamical state data of each cell, conflicts where output states are different for the same input states can happen due to the noisy nature of single‐cell data and the stochasticity of cells. To resolve these conflicts, the number of different outputs for a specific input condition in the pseudo‐truth table can be counted, and the output with the higher count was selected to refine the truth table. The refined truth table can determine a minimized regulation logic with determined outputs for input conditions not defined in the truth table (“don't care” terms), using the QM algorithm. This logic inference and can be repeated for all genes in the network to determine the initial Boolean GRN model. However, during the logic minimization process, some genes might be inferred to be always True or False due to the characteristics of the truth table. In such cases, the canalization effect—where some genes can fix the values of their downstream genes irrespective of other remaining genes in the network—is identified and reflected. By removing these fixed‐value genes, the final Boolean GRN model is determined.

### Attractor Analysis

An attractor refers to a steady state where a specific initial state in the state space of a system converges to after state transitions. These attractors are associated with distinct cellular phenotypes.^[^ ^29^ ^]^ To assess whether the reconstructed Boolean GRN model accurately captures the transition dynamics from the colonic stem cell state to the enterocyte state and to simulate the perturbation effects of control targets, attractor simulations using a synchronous state update scheme, starting from all initial states, were conducted. Furthermore, to investigate how the perturbation of control targets can lead to a state transition from colonic stem cell state to enterocyte state, attractor simulations using the synchronous scheme were performed. Here, perturbation refers to the permanent fixation of a node's state as either OFF or ON.

### Mapping the Attractors onto the UMAP Embedding Space

To align the attractors from the Boolean GRN model with the single‐cell data visualized in the UMAP embedding space, following analyses were performed. For each attractor, the nearest single‐cell based on the Hamming distance between the attractor state and the binarized expression state of cells was identified. The most proximate cell was determined by the lowest Hamming distance. Using the pseudotime assigned to each cell, the pseudotime was computed from the most proximate cell identified in the previous step. This process aimed to temporally position the attractor within the differentiation trajectory. In this way, the cell whose pseudotime most closely matches the computed pseudotime was selected as the representative of the attractor in the UMAP embedding space.

### BNsimpleReduction

The BNsimpleReduction algorithm is a kind of a control scheme designed to achieve global stabilization of complex biological networks. This algorithm begins with a simple state‐space coordinate transformation, thereby converting the original Boolean network into an equivalent configuration that facilitates global stabilization to a desired attractor. A network reduction is then implemented on the transformed Boolean network while preserving the essential topology associated with the desired attractor ensuring that the reduced Boolean network still possesses the information on the desired attractor. Then, the control targets are derived by searching for a minimum set of nodes in the reduced Boolean network, whose permanent perturbation guarantees that the topology is acyclic. FVS control algorithm is employed to identify appropriate control targets. The proposed control scheme guarantees global stabilization while maintaining a manageable level of computational complexity and ensures the number of control targets identified by the control scheme is close to optimality.

### Average Activity Vector

The average activity vector was derived by computing a weighted sum of the attractor state vectors composed of 0's and 1's, where each attractor state vector is multiplied by its corresponding ratio of basin of attraction, and the products are then element‐wisely added together. In the case of cyclic attractors, the ratio of basin of attraction by the number of states within each cyclic attractor was divided and this scalar value was then multiplied by each state vector in the cyclic attractor, and the resulting products are element‐wisely added together. The average activity vector is used to derive the similarity with the desired state. The formula for calculating the average activity vector is as follows:

(1) $$\begin{array}{ccc} & & \mathrm{Average}\;\mathrm{activity}\;\mathrm{vector}\\ & & =\frac{1}{n}\left(\sum_{i=1}^{n}\mathrm{Attractor}\;\mathrm{state}\;\mathrm{vecto}r_{i}\cdot \frac{\mathrm{Ratio}\;\mathrm{of}\;\mathrm{basin}\;\mathrm{of}\;\mathrm{attraction}}{\mathrm{Number}\;\mathrm{of}\;\mathrm{states}}\right) \end{array}$$

### Similarity between the Desired Attractor State and the Average Activity Vector of the Perturbed Network

The similarity between the desired state vector and each average activity vector was computed. Among various similarity measures, the widely used and intuitively understood cosine similarity measure was utilized. Cosine similarity is determined by the angle (theta) between two vectors, and a value close to 1 indicates a high degree of similarity, while a value close to 0 indicates dissimilarity. The formula for cosine similarity is as follows:

(2) $$\mathrm{Similarity}=\frac{\mathrm{Desired}\;\mathrm{attractor}\;\mathrm{state}\;\mathrm{vector}\cdot \mathrm{Average}\;\mathrm{activity}\;\mathrm{vector}}{\left|\mathrm{Desired}\;\mathrm{attractor}\;\mathrm{state}\;\mathrm{vector}\right|\cdot \left|\mathrm{Average}\;\mathrm{activity}\;\mathrm{vector}\right|}$$

### Synergy Score of Identified Control Targets

The synergy score measures the number of canalized nodes when all possible combinations of nodes are perturbed together, divided by the additive score, which is the number of canalized nodes that each node canalizes independently. The formula for synergy score is as follows:

(3) $$\mathrm{Synergy}\;\mathrm{score}=\frac{\mathrm{Number}\;\mathrm{of}\;\mathrm{canalized}\;\mathrm{nodes}}{\mathrm{Additive}\;\mathrm{score}}$$

### scTenifoldKnk In Silico Knockout Analysis

The analysis of virtual knockout was conducted using the scTenifoldKnk tool.^[^ ^31^ ^]^ The ribosomal and mitochondrial genes were removed from the intestinal stem cell raw count matrix, considering only those genes expressed in over 15% of the total cell population for the analysis. In silico knockout analysis was performed using default parameters. For the genes affected by virtual knockout perturbations, functional annotation, and enrichment analysis were carried out using the Enrichr software package.^[^ ^59^ ^]^

### GSEA Analysis of Three Types of Colon Cancer Cell Line

Cell lines were compared with knockdown of all three control targets against wild‐type cell lines, ranking all genes based on the magnitude of differential gene expression. Subsequently, these ranked genes were cross‐referenced with enterocyte,^[^ ^60^ ^]^ Wnt,^[^ ^61^ ^]^ and Myc^[^ ^61^ ^]^ signatures using Gene Set Enrichment Analysis (GSEA). GSEA was performed utilizing the “fgsea” (fast GSEA) R‐package,^[^ ^62^ ^]^ according to its default parameters.

### Cell Culture and Reagents

Human colon cancer cells, HT‐29, CACO‐2, and HCT‐116, obtained from the Korean Cell Line Bank and normal human colon epithelial cell line, NCM‐460, a kind gift from Seyun Kim (Korea Advanced Institute of Science and Technology) were cultured in Dulbecco's modified Eagle's medium (DMEM) (WelGENE Inc.) containing 10% fetal bovine serum (FBS, WelGENE Inc.) and antibiotics (100 units mL^−1^ of penicillin, 100 µg mL^−1^ streptomycin and 0.25 µg mL^−1^ of Fungizone) (Life Technologies Corp.) at 37 °C in a humidified atmosphere containing 5% CO_2_.

### Virus Production and Establishment of Stable Cell Lines

Lentiviral particles were generated by transfecting HEK293T cells with lentivirus mediated short hairpin RNA plasmid (Sigma‐Aldrich), packaging and envelope plasmid mixture (pLP1, pLP2, pLP/VSVG, Invitrogen) using Lipofectamine 2000 (Invitrogen) according to the manufacturer's instructions. Moreover, cells overexpressing MYB, HDAC2, and FOXA2 using lentiviral particles were established. Lentiviral particles were generated by transfecting HEK293T cells with MYB, HDAC2, and FOXA2 overexpression vector (Vector Builder), psPAX2 packaging (a gift from Didier Trono, Addgene # 12260), and pMD2.G envelope (a gift from Didier Trono, Addgene # 12259) using Lipofectamine 2000 (Invitrogen). The medium was harvested after 48 h of transfection and filtered through a 0.22‐µm cellulose acetate filter (Sartorius). Filtered lentiviral medium and complete DMEM 10% supplemented with 8 µg mL^−1^ polybrene were infected to NCM‐460, HT‐29, CACO‐2, and HCT‐116 cell lines. shRNA sequences used are given in Table S9 (Supporting Information), and overexpression vector map is given in Figure S18A (Supporting Information).

### Total RNA Extraction and qRT‐PCR

RNA was extracted using an RNA‐spin kit (INTRON), and cDNA was synthesized using a DiaStar RT kit (Solgent) according to the manufacturer's instructions. After reverse transcription, qRT‐PCR was performed using QuantStudio5 (Applied Biosystems) with SYBR Master Mix (GeNet Bio). qRT‐PCR primer sequences used are given in Table S8 (Supporting Information).

### Alignment and Pre‐Processing of Bulk RNA‐Seq Data of Colon cancer Cell Lines

Sequencing libraries were prepared using TruSeq RNA Sample Preparation kit v2 (Illumina Inc., USA). After pooled libraries were denatured, sequencing of each library was carried out using the 100 bp paired‐end mode of the TruSeq Rapid PE Cluster Kit and TruSeq Rapid SBS Kit with HiSeq 2500 (Illumina Inc., USA). To quantify total RNA of colorectal cancer cell lines, sequencing libraries were prepared using TruSeq RNA Sample Preparation kit v2. After pooled libraries were denatured, sequencing of each library was carried out using the 100 bp paired‐end mode of the TruSeq Rapid PE Cluster Kit and TruSeq Rapid SBS Kit with HiSeq2500 (Illumina Inc., USA). The prepared RNA‐seq data were trimmed using Trimmomatic version 0.39. The trimmed reads were aligned to the mm10 reference genome using STAR version 2.7.7a with the default parameter. The mapped reads were indexed and sorted by samtools version 1.7. Then HTSeq version 0.12.4 was used to quantify read coverage per gene. For all human RNA seq data, the alignment pipeline (Trimmomatic—STAR—HTSeq) with hg38 reference genome was also performed. Next, batch‐effect corrections were performed by ComBat‐seq.

### Western Blot Analysis

Cells were washed in PBS and lysed in lysis buffer [20 mm HEPES pH 7.2, 0.5% Triton X‐100, 150 mm NaCl, 10% Glycerol, protease/phosphatase inhibitor cocktail (Thermo Fisher)]. The lysates were centrifuged at 13 000 rpm for 15 min at 4 °C and the supernatants were separated by SDS‐PAGE followed by immunoblotting. For immunoblotting, anti‐HDAC2, anti‐FOXA2, anti‐c‐MYB, anti‐KRT20, anti‐TCF1/7, anti‐c‐MYC, anti‐VDR, anti‐β‐catenin, and anti‐GAPDH purchased from Cell Signaling Technology Inc., and anti‐KRT19 purchased from Santa Cruz Biotechnology Inc. were used. For quantifying intensity of protein bands, ImageJ software was used (http://imagej.nih.gov/ij) and normalized by GAPDH.

### Cell Growth Assay

To analyze the effect of control targets on cell growth, transfected cells were seeded in 96‐well plate (4  ×  10^3^ cells per well). Cell images were captured every 3 h interval with IncuCyte ZOOM (Sartorius). Cell growth was analyzed based on confluence of adherent living cell using IncuCyte software.

### Mouse Injection Condition

Athymic NCr‐nu/nu [7 weeks per female] (mouse) (Koatech) were implanted subcutaneously with 2  ×  10^6^ HCT‐116 cells, 1.5  ×  10^6^ HT‐29 cells, and 3  ×  10^6^ CACO‐2 cells (in 100 µL PBS). Tumor growth was measured using digital caliper and tumor volume was calculated as 0.5 × long length  × short length^2^ (mm^3^). All animal procedures were IACUC approved (KA2019‐24) and performed in Laboratory Animal Research Center at Korea Advanced Institute of Science and Technology. Athymic Balb/c nude mice were purchased from Koatech (Korea). Mice were housed in the ventilated cage (max 5 mice per cage) supplied with food and water in a 12‐h light/12‐h dark cycle at 22 °C and 41% humidity.

### Statistical Analysis

Wilcoxon rank‐sum test or *t*‐test was used to evaluate differences between groups for continuous variables. Overall Survival (OS) was defined as the time interval between dates of curative surgery and death from any cause. Disease Free Survival (DFS) was defined as the time interval between dates of curative surgery and tumor recurrence or death. OS and DFS rates were estimated using the Kaplan–Meier method and were compared based on survival distributions between two groups using the log‐rank test. The Cox proportional hazards model was used for univariate and multivariate analysis. Clinicopathologic factors, which were statistically significant in univariate analysis, were included as covariables in multivariate analysis. Hazard ratios (HR) and 95% confidence intervals (CI) were assessed for each factor. All tests were two‐sided, and a *p*‐value of less than 0.05 was considered statistically significant.

## Conflict of Interest

K.‐H.C. is an inventor of patents licensed to, board member of and equity owner of biorevert, Inc. No disclosures were reported by the other authors.

## Author Contributions

J.‐R.G., C.‐K.L., H.‐M.K., and J.K. are co‐first authors and contributed equally to this work. K.‐H.C. conceived the study. J.‐R.G., C.‐K.L., and H.‐M.K. developed BENEIN framework and performed Boolean network simulation and analysis. J.‐R.G. and J.K. designed data analysis and in vitro and in vivo experiments. J.‐R.G., J.K., J.J., and S.P. performed in vitro and in vivo experiments. All authors wrote the manuscript and reviewed and discussed the manuscript. K.‐H.C. supervised all aspects of the work and approved the final manuscript.

## Supporting information



Supporting Information

Supporting Information

Supporting Information

Supporting Information

Supporting Information

Supporting Information

Supporting Information

Supporting Information

Supporting Information

## Data Availability

Data sharing is not applicable to this article as no new data were created or analyzed in this study.
