# Correction to “Control of Cellular Differentiation Trajectories for Cancer Reversion”

**DOI:** 10.1002/advs.75708

**Published:** 2026-05-15

Jeong‐Ryeol Gong, Chun‐Kyung Lee, Hoon‐Min Kim, Juhee Kim, Jaeog Jeon, Sunmin Park, and Kwang‐Hyun Cho, “Control of Cellular Differentiation Trajectories for Cancer Reversion,” *Advanced Science* 12, no. 3 (2025): e2402132, https://doi.org/10.1002/advs.202402132.

**Data Availability Statement**

The “Data Availability” statement is incorrect as presented. The authors have provided the following statement to be used in place of the published statement:

“The RNA‐seq data of three colorectal cancer cell lines (HCT116, CACO2, and HT29) with scramble shRNA control and simultaneous knockdown of MYB, HDAC2, and FOXA2 have been deposited in the NCBI Gene Expression Omnibus (GEO) under accession number GSE327208 (https://www.ncbi.nlm.nih.gov/geo/query/acc.cgi?acc=GSE327208).”

