# Supplementary material for: Control of Cellular Differentiation Trajectories for Cancer Reversion
Source: Adv Sci (Weinh). 2024 Dec 11;12(3):2402132. doi: 10.1002/advs.202402132 (PMC11744559; doi:10.1002/advs.202402132)
Supplement: Supplementary file 1 — Supporting Information [file ADVS-12-2402132-s002.pdf]

ADVANCED SCIENCE
Open Access

Supporting Information

for *Adv. Sci.*, DOI 10.1002/advs.202402132

Control of Cellular Differentiation Trajectories for Cancer Reversion

*Jeong-Ryeol Gong, Chun-Kyung Lee, Hoon-Min Kim, Juhee Kim, Jaeog Jeon, Sunmin Park and Kwang-Hyun Cho**

Supporting Information

Control of cellular differentiation trajectories for cancer reversion

*Jeong-Ryeol Gong, Chun-Kyung Lee, Hoon-Min Kim, Juhee Kim, Jaeog Jeon, Sunmin Park, and Kwang-Hyun Cho**

This file includes:

Supplementary Figures S1 to S21

Other supplementary materials for this manuscript include the following:

Supplementary Tables S1, S8, and S9

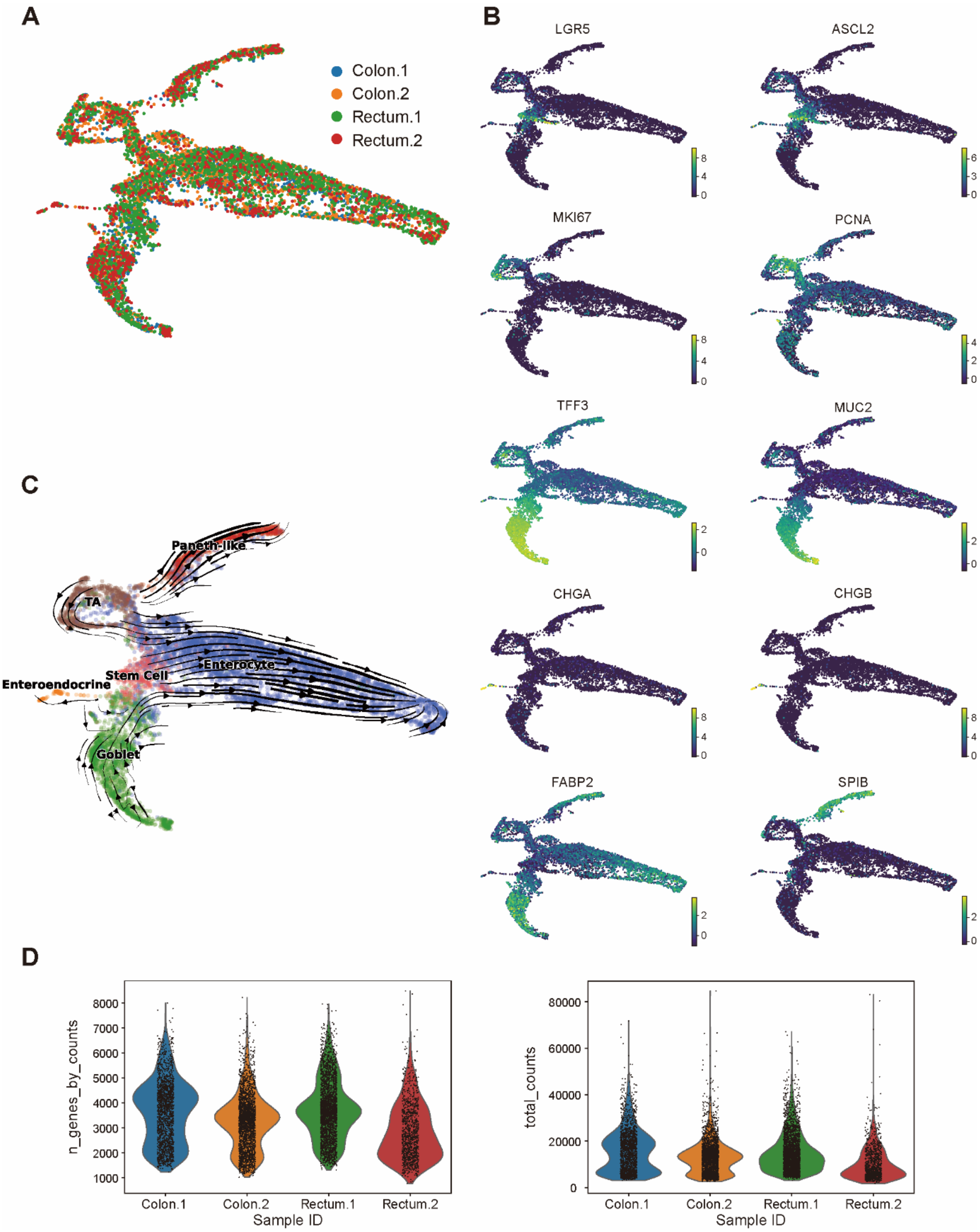

Figure S1. Preprocessing the single cell transcriptome data of human colon differentiation
(A) UMAP embedding of human intestinal data overlaid with sample ID.
(B) UMAP embedding overlaid with scaled marker gene expression for stem cell (LGR5, ASCL2), transit amplifying (TA) cell (MKI67, PCNA), goblet cell (TFF3, MUC2), enteroendocrine cell (CHGA, CHGB), enterocyte (FABP2), and paneth-like cell (SPIB).
(C) RNA velocity stream plot over UMAP embedding overlaid with annotated cell types.
(D) Violin plot of the number of genes per cell, and total counts grouped by sample ID.

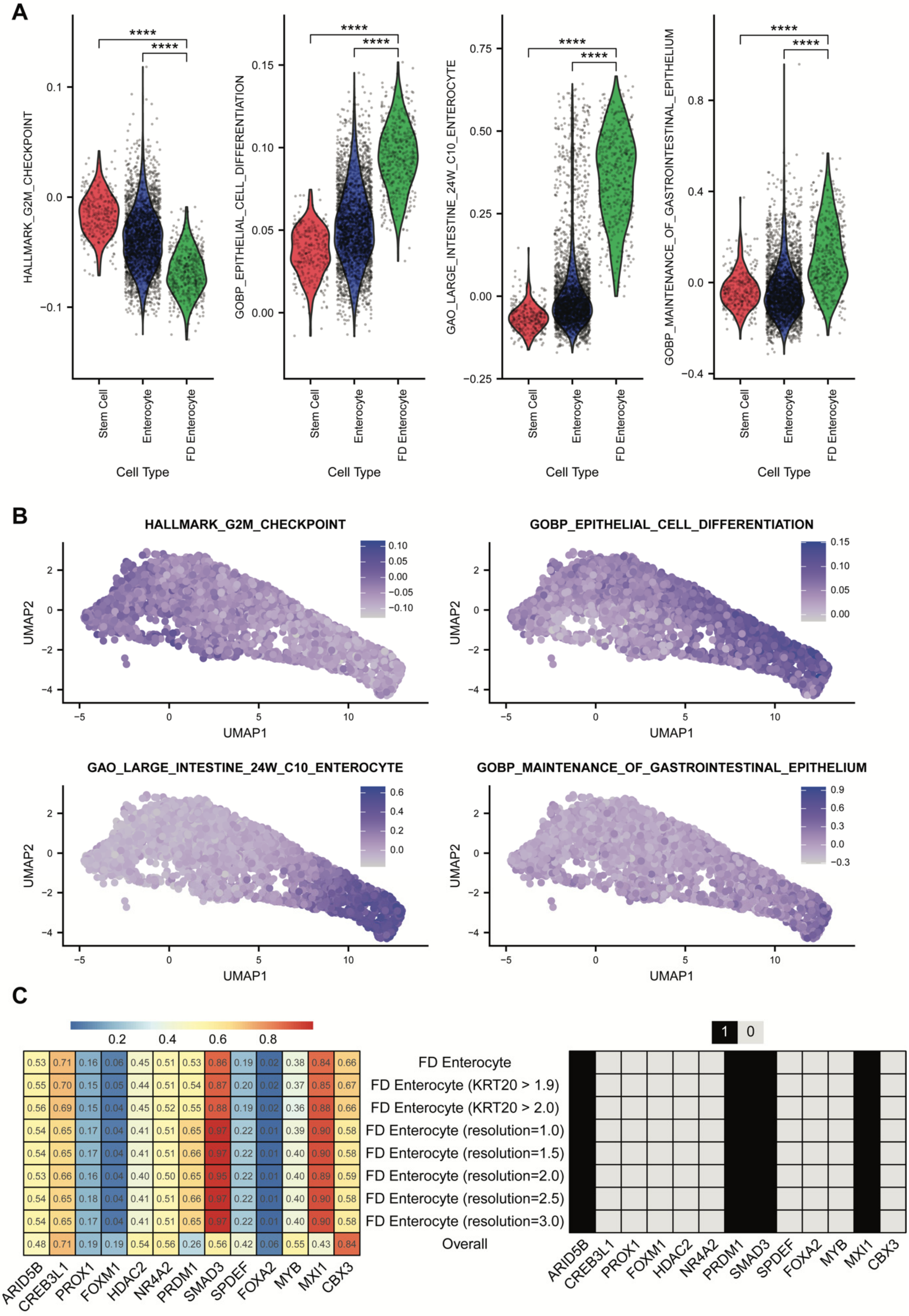
A
HALLMARK_G2M_CHECKPOINT
GOBP_EPITHELIAL_CELL_DIFFERENTIATION
GAO_LARGE_INTESTINE_24W_C10_ENTEROCYTE
GOBP_MAINTENANCE_OF_GASTROINTESTINAL_EPITHELIUM
****
Stem Cell
Enterocyte
FD Enterocyte
Cell Type
B
HALLMARK_G2M_CHECKPOINT
GOBP_EPITHELIAL_CELL_DIFFERENTIATION
GAO_LARGE_INTESTINE_24W_C10_ENTEROCYTE
GOBP_MAINTENANCE_OF_GASTROINTESTINAL_EPITHELIUM
UMAP1
UMAP2
C
0.2 0.4 0.6 0.8
0.53 0.71 0.16 0.06 0.45 0.51 0.53 0.86 0.19 0.02 0.38 0.84 0.66
0.55 0.70 0.15 0.05 0.44 0.51 0.54 0.87 0.20 0.02 0.37 0.85 0.67
0.56 0.69 0.15 0.04 0.45 0.52 0.55 0.88 0.19 0.02 0.36 0.88 0.66
0.54 0.65 0.17 0.04 0.41 0.51 0.65 0.97 0.22 0.01 0.39 0.90 0.58
0.54 0.65 0.17 0.04 0.41 0.51 0.66 0.97 0.22 0.01 0.40 0.90 0.58
0.53 0.66 0.16 0.04 0.40 0.50 0.65 0.95 0.22 0.01 0.40 0.89 0.59
0.54 0.65 0.18 0.04 0.41 0.51 0.66 0.97 0.22 0.01 0.40 0.90 0.58
0.54 0.65 0.17 0.04 0.41 0.51 0.65 0.97 0.22 0.01 0.40 0.90 0.58
0.48 0.71 0.19 0.19 0.54 0.56 0.26 0.56 0.42 0.06 0.55 0.43 0.84
FD Enterocyte
FD Enterocyte (KRT20 > 1.9)
FD Enterocyte (KRT20 > 2.0)
FD Enterocyte (resolution=1.0)
FD Enterocyte (resolution=1.5)
FD Enterocyte (resolution=2.0)
FD Enterocyte (resolution=2.5)
FD Enterocyte (resolution=3.0)
Overall
1 0
ARID5B CREB3L1 PROX1 FOXM1 HDAC2 NR4A2 PRDM1 SMAD3 SPDEF FOXA2 MYB MXI1 CBX3

Figure S2. Fully differentiated enterocyte state shows a clear distinction as a desired state and is robustly defined across various criteria for determining FD enterocytes
(A) Violin plot of signature scores related to fully differentiated enterocytes grouped by cell types.
(B) Signature scores related to fully differentiated enterocytes in the UMAP embedding space.
(C) Heatmaps of average binarized expressions (left) and binarized states (right) of the genes in the GRN model of FD enterocyte determined with different clustering resolutions and cutoffs of KRT20 expression. (Wilcoxon rank-sum test: **** $p < 0.0001$)

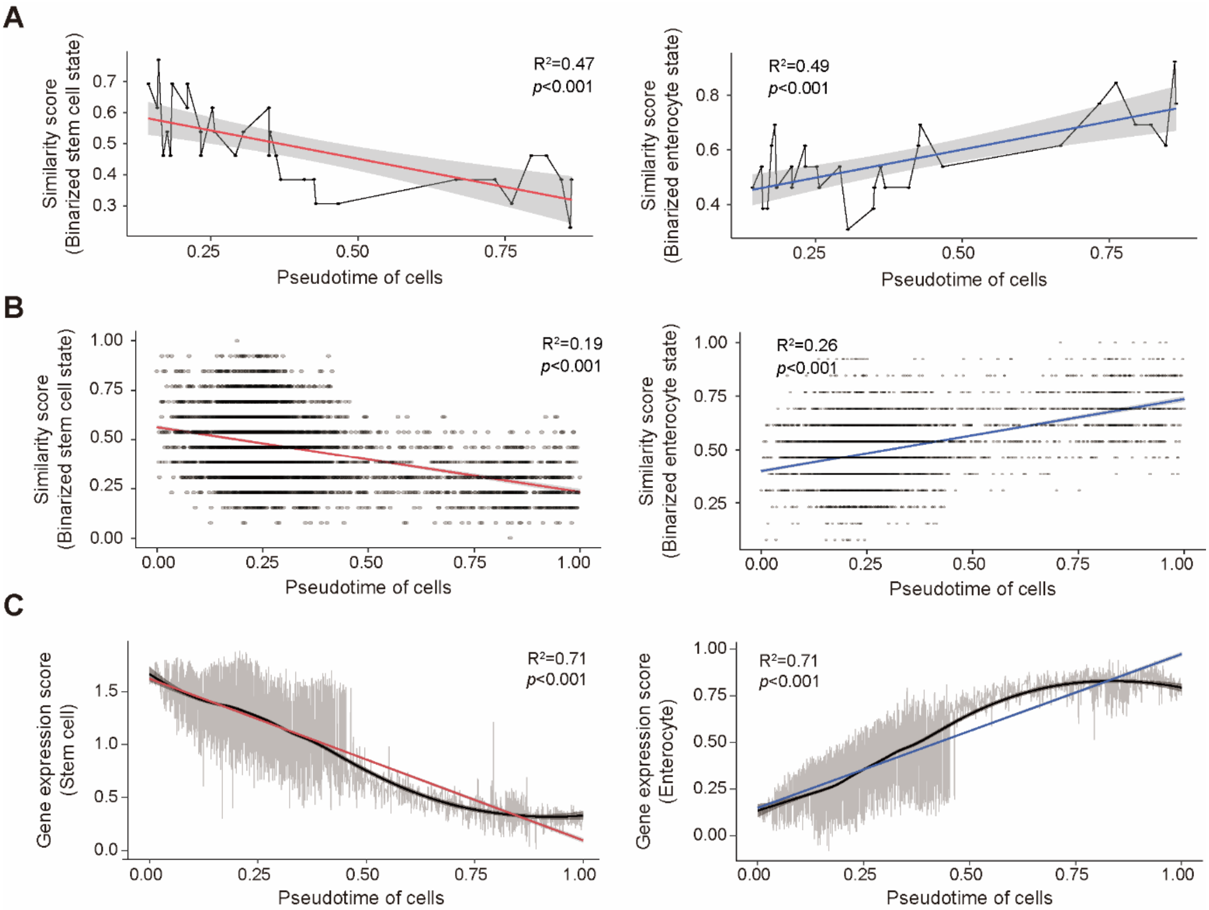

Figure S3. Comparative analysis of similarity scores and gene expression scores between single cell transcriptome data and the BENEIN-constructed Boolean GRN model dynamics over the pseudotime of enterocyte differentiation trajectory

(A) Similarity scores for stem cell and enterocyte states of the 32 point attractors of the GRN model across pseudotime.

(B) Similarity scores of binarized cell states derived from single cell transcriptome data for each cell type across pseudotime.

(C) Gene expression scores calculated using differentially expressed genes for each cell type from the single cell transcriptome data across pseudotime. Together, these results indicate that both the scores derived from the single cell transcriptome data and the dynamics of the Boolean GRN model show a decreasing trend for stem cell scores and an increasing trend for enterocyte scores over pseudotime. This underscores that the Boolean GRN model can properly reproduce the primary dynamics along the differentiation trajectory.

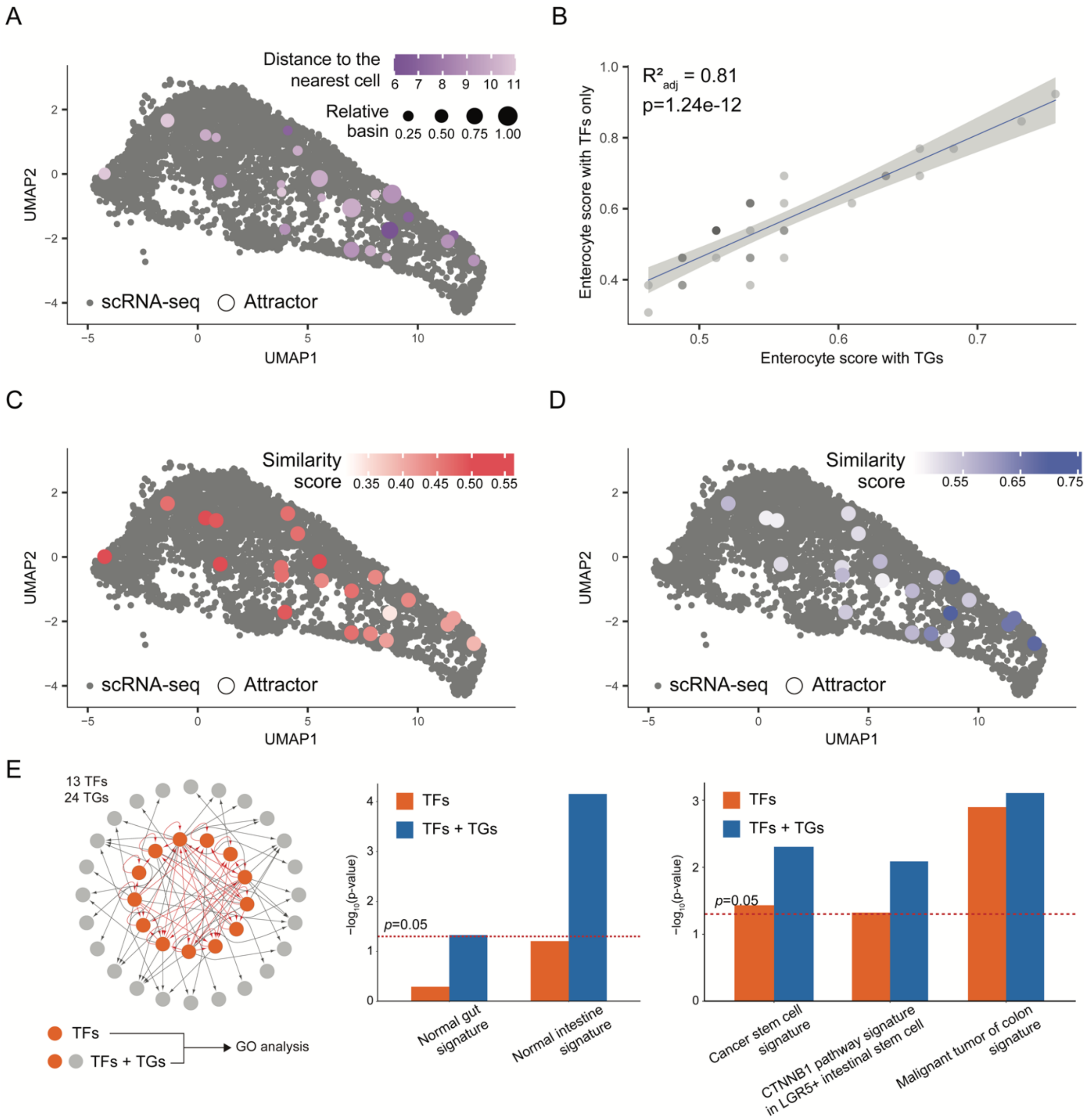

Figure S4. Analysis of the Boolean GRN model including the target genes
(A) 32 point attractors of the Boolean GRN model including TGs mapped onto the UMAP embedding space, with the relative basin of attraction and Hamming distance between each attractor and binarized expression of the nearest cell.
(B) Similarity scores of the attractors with respect to the enterocyte state with TGs (x-axis) and without TGs (y-axis).
(C and D) Similarity scores of the attractors with respect to the stem cell state (C) and the enterocyte state (D) upon the UMAP embedding space.

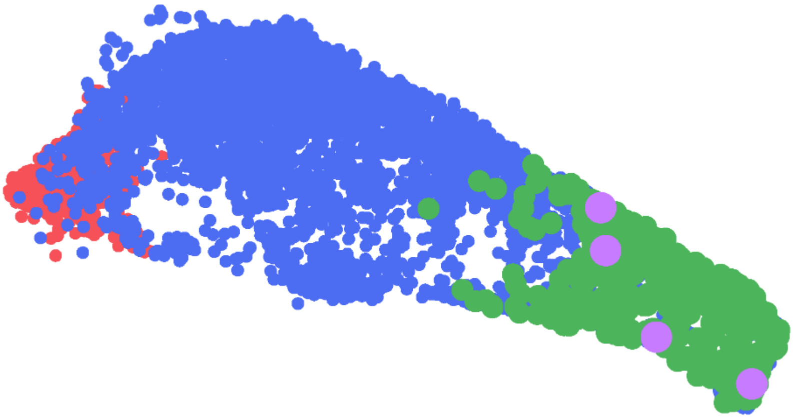

Figure S5. The attractors phenotype after controlling the identified control targets in the Boolean GRN model exhibited the fully differentiated (FD) enterocyte
Four attractors emerge when the identified three control targets are controlled (simultaneous inhibition of FOXA2, MYB, HDAC2) in the Boolean GRN model. When mapped onto the UMAP embedding space, these four attractors are included in the FD enterocyte cluster where KRT20 is highly expressed. Therefore, the phenotype of these four attractors is FD enterocyte, which suggests that the Boolean GRN model is controlled into the enterocyte state when the three control targets are controlled.

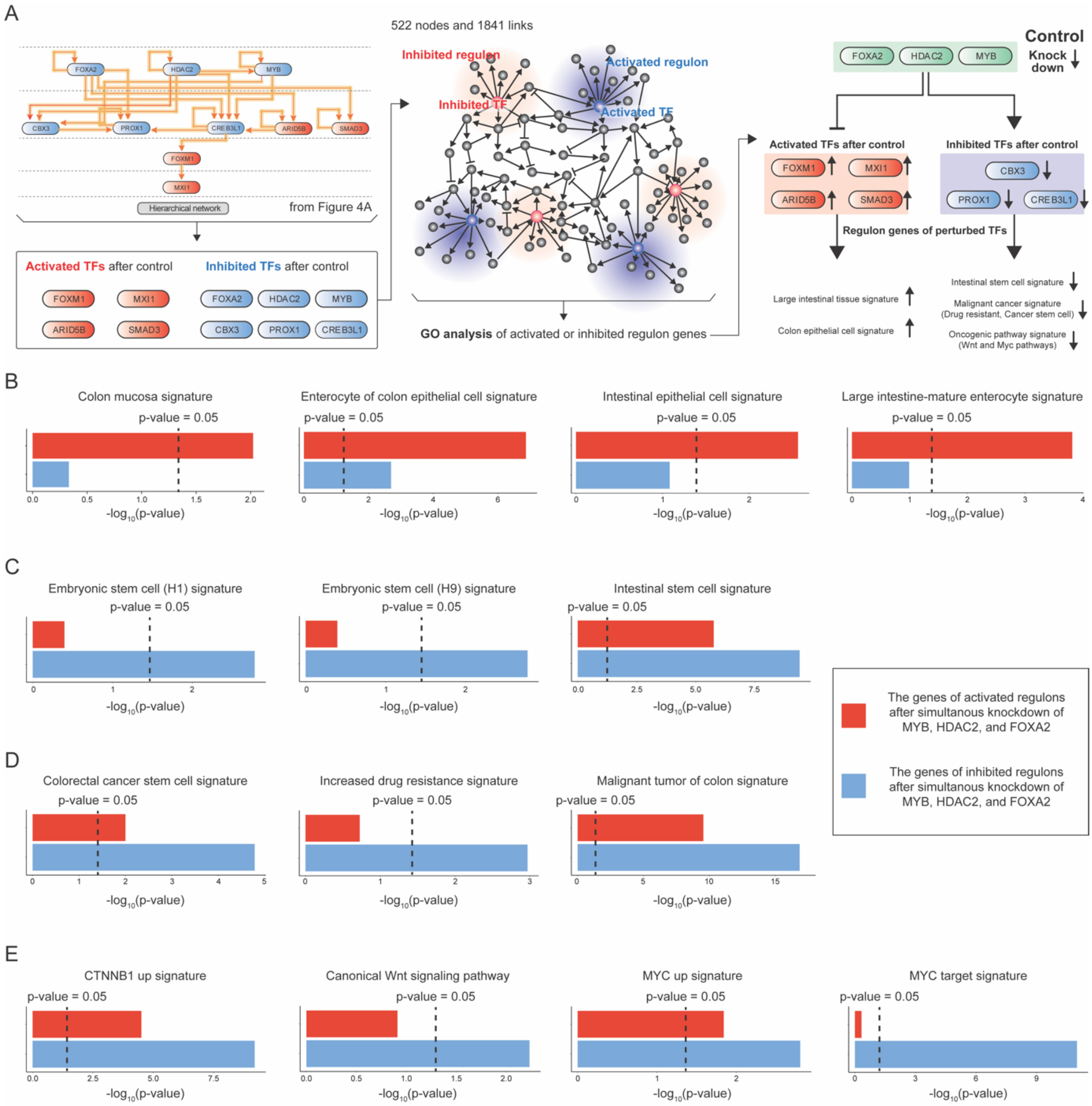

Figure S6. GO analysis reveals that TGs could be important for differentiation and cancer reversion

(A) The schematic figure illustrates the process of selecting activated TFs and inhibited TFs in the Boolean GRN model, as well as identifying the state of genes in their regulons.

(B) The TGs regulated by the activated TFs following a simultaneous knockdown of MYB, HDAC2, and FOXA2 are strongly associated with normal enterocyte function.

(C-E) The TGs regulated by the inhibited TFs are significantly related to the characteristics of cancer cells, particularly stemness (C), colon cancer signature (D), and WNT and MYC pathways (E).

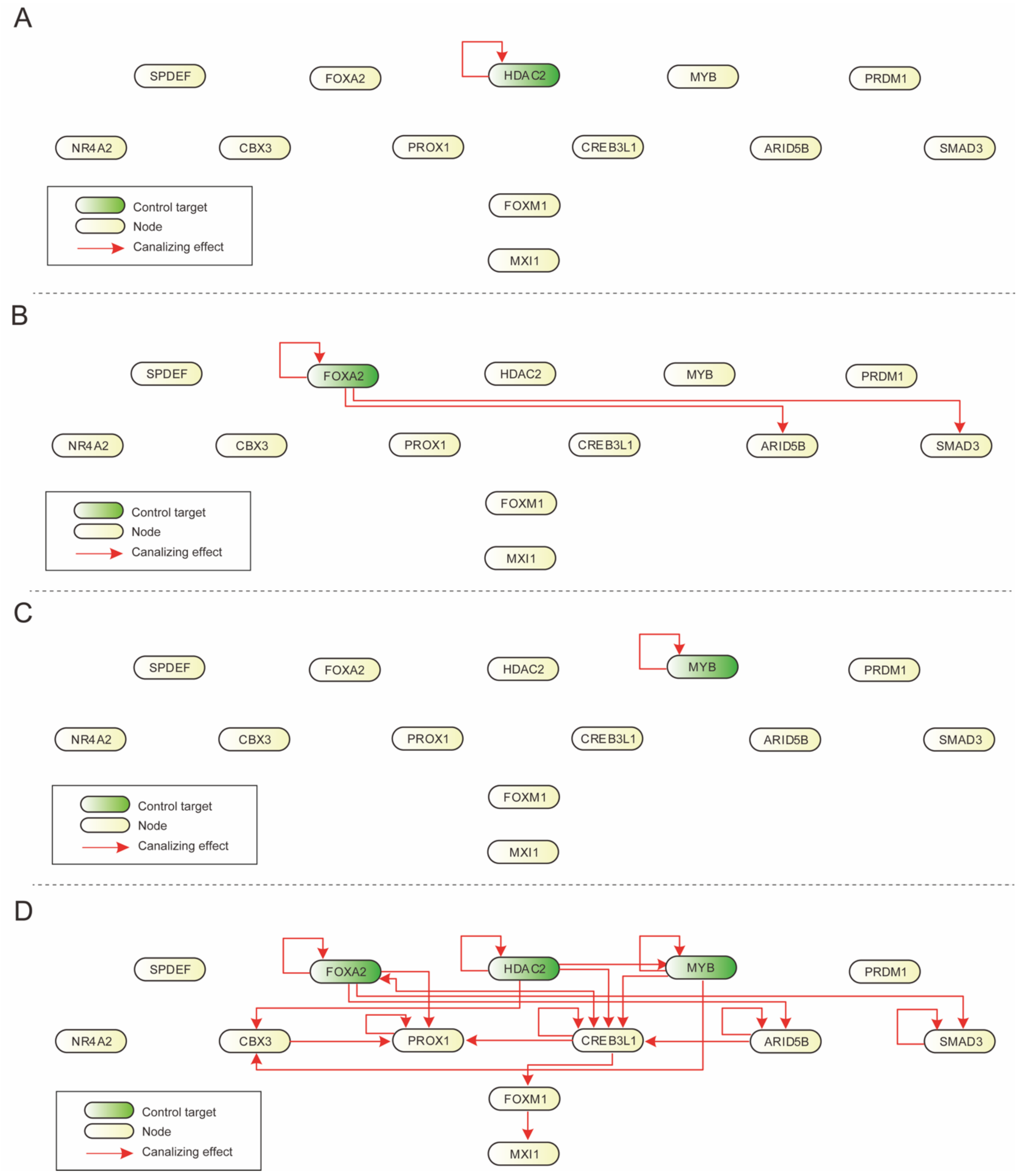

Figure S7. Canalizing effects of MYB, HDAC2, and FOXA2 on the reduced network
Canalizing effects of HDAC2(A), FOXA2 (B), MYB (C), and all three targets (MYB, HDAC2, and FOXA2) (D). The nodes are rearranged based on the reduced network by BNSimpleReduction to show their hierarchical structure. Perturbed nodes are colored green in each of the networks, and their canalizing effects on the downstream nodes are connected with red edges.

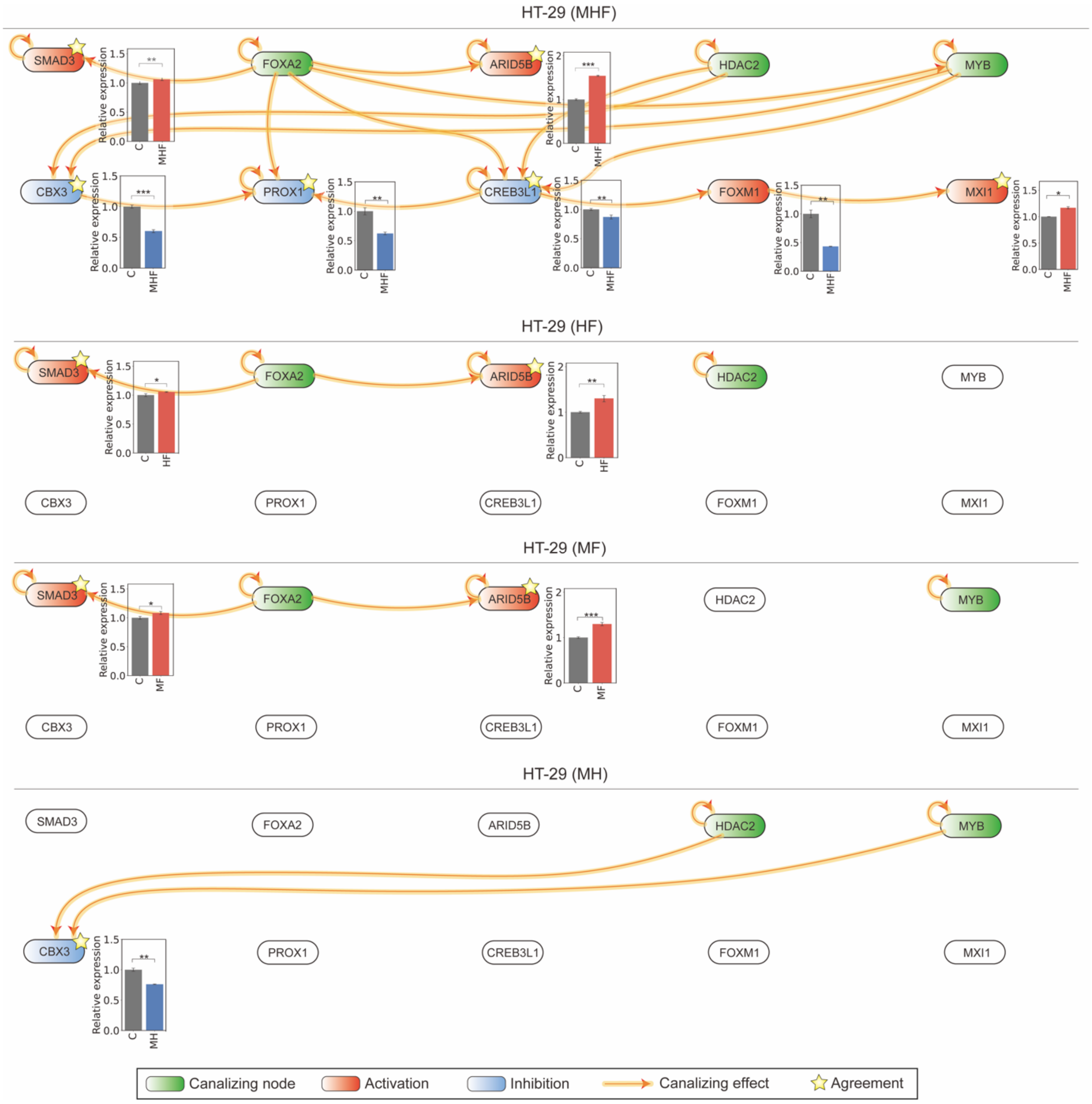

Figure S8. Details of the comparative analysis of the canalizing effect from the simultaneous perturbation of MYB, HDAC2, and FOXA2 in the Boolean GRN model with the *in vitro* transcript quantification on HT-29

The network is a subnetwork of the reduced network consisting of the canalized nodes. Nodes are colored red (activation) or blue (inhibition) according to the canalization effect. The *in vitro* transcript quantification results are shown in bar charts, with a scramble knockdown sample colored in grey and knockdown samples colored in red (up-regulated) or blue (down-regulated). The discrepancy between the experiments and the simulation results is attributed to differences in the regulatory mechanisms between normal and cancer cells. When knockdown experiments were further conducted in a normal human colon cell line, it was found that the resulting gene expressions were well aligned with the simulation predictions. Even though some discrepancies were observed, when simultaneous knockdown of the identified combination targets (MYB, HDAC2, and FOXA2) was performed in colon cancer cells, the expected reversion from a cancer cell state to a normal-like cell state was still effectively induced. Data are presented as the mean ± SEM; n = 3 measurements (two-tailed t-test: *$p < 0.05$, **$p < 0.01$, ***$p < 0.001$) (HT-29 with scramble knockdown, C; double knockdown MYB+HDAC2, MH; MYB+FOXA2, MF; HDAC2+FOXA2, HF).

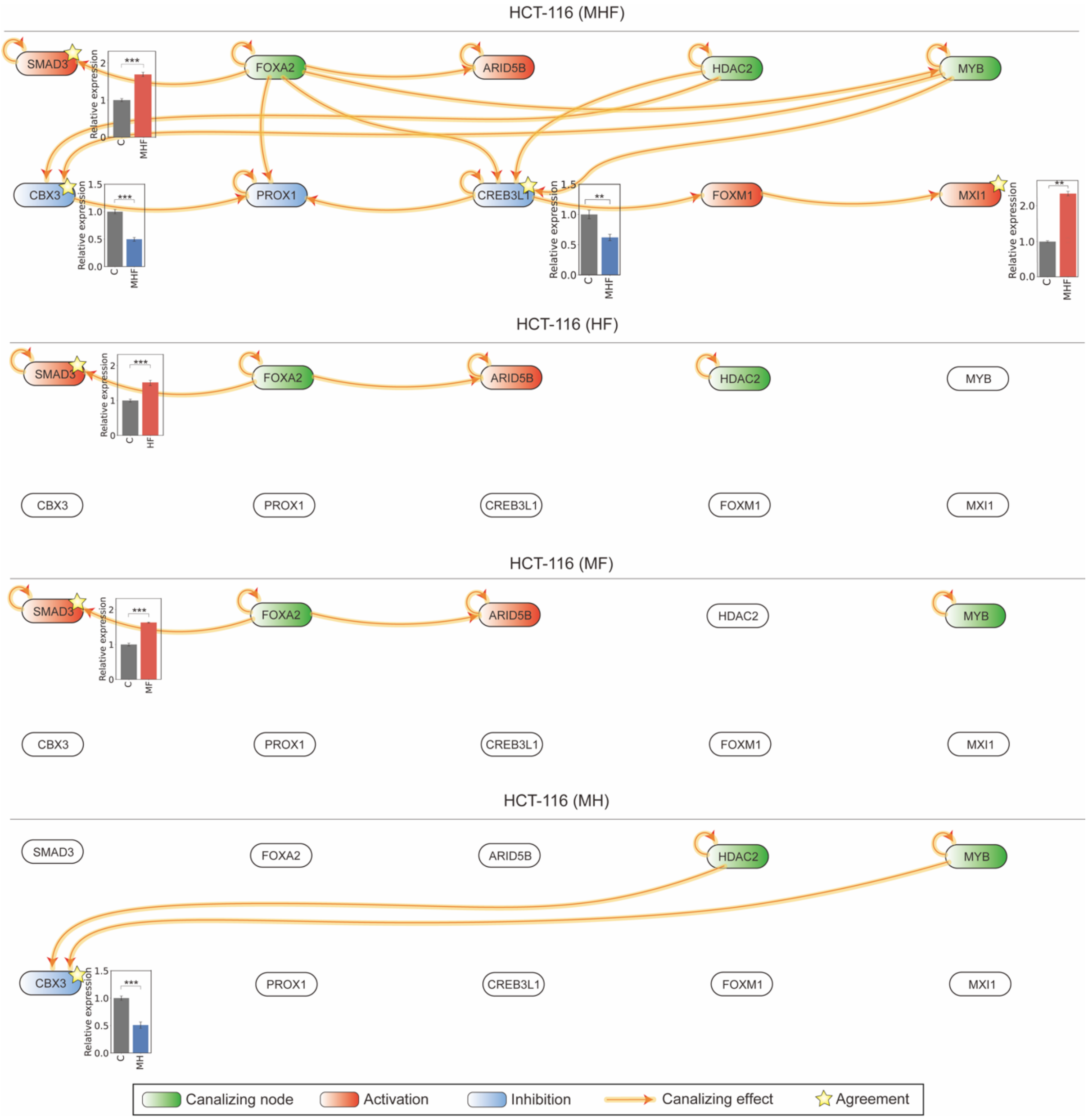

Figure S9. Details of the comparative analysis of the canalizing effect from the simultaneous perturbation of MYB, HDAC2, and FOXA2 in the Boolean GRN model with the *in vitro* transcript quantification on HCT-116

The network is a subnetwork of the reduced network consisting of the canalized nodes. Nodes are colored red (activation) or blue (inhibition) according to the canalization effect. The *in vitro* transcript quantification results are shown in bar charts, with a scramble knockdown sample colored in grey and knockdown samples colored in red (up-regulated) or blue (down-regulated). The discrepancy between the experiments and the simulation results is attributed to differences in the regulatory mechanisms between normal and cancer cells. When knockdown experiments were further conducted in a normal human colon cell line, it was found that the resulting gene expressions were well aligned with the simulation predictions. Even though some discrepancies were observed, when simultaneous knockdown of the identified combination targets (MYB, HDAC2, and FOXA2) was performed in colon cancer cells, the expected reversion from a cancer cell state to a normal-like cell state was still effectively induced. Data are presented as the mean ± SEM; n = 3 measurements (two-tailed t-test: $^{*}p < 0.05$, $^{**}p < 0.01$, $^{***}p < 0.001$) (HCT-116 with scramble knockdown, C; double knockdown MYB+HDAC2, MH; MYB+FOXA2, MF; HDAC2+FOXA2, HF and triple knockdown MYB+HDAC2+FOXA2, MHF).

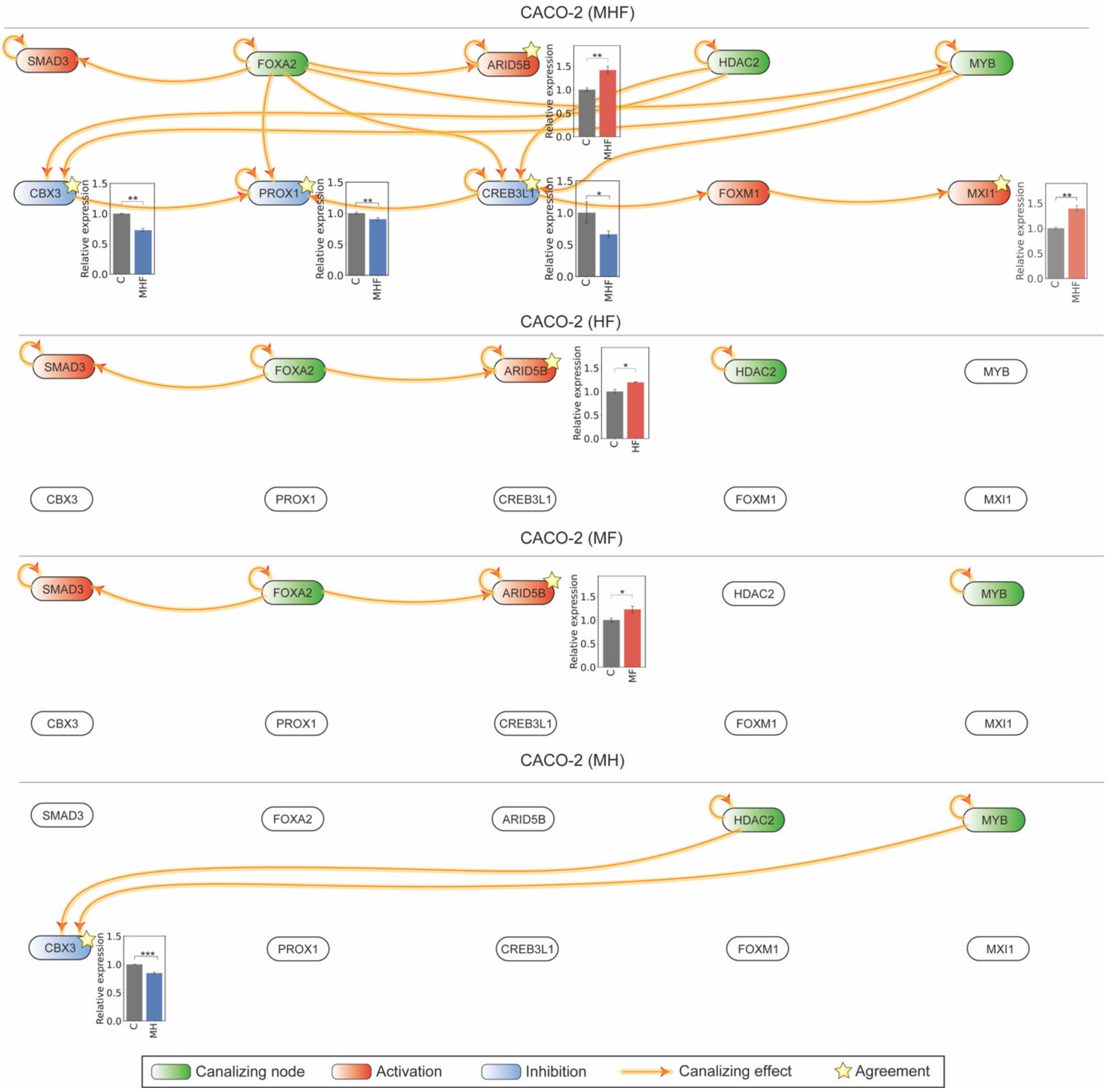

Figure S10. Details of the comparative analysis of the canalizing effect from the simultaneous perturbation of MYB, HDAC2, and FOXA2 in the Boolean GRN model with the *in vitro* transcript quantification on CACO-2

The network is a subnetwork of the reduced network consisting of the canalized nodes. Nodes are colored red (activation) or blue (inhibition) according to the canalization effect. The *in vitro* transcript quantification results are shown in bar charts, with a scramble knockdown sample colored in grey and knockdown samples colored in red (up-regulated) or blue (down-regulated). The discrepancy between the experiments and the simulation results is attributed to differences in the regulatory mechanisms between normal and cancer cells. When knockdown experiments were further conducted in a normal human colon cell line, it was found that the resulting gene expressions were well aligned with the simulation predictions. Even though some discrepancies were observed, when simultaneous knockdown of the identified combination targets (MYB, HDAC2, and FOXA2) was performed in colon cancer cells, the expected reversion from a cancer cell state to a normal-like cell state was still effectively induced. Data are presented as the mean ± SEM; n = 3 measurements (two-tailed t-test: *$p < 0.05$, **$p < 0.01$, ***$p < 0.001$) (CACO-2 with scramble knockdown, C; double knockdown MYB+HDAC2, MH; MYB+FOXA2, MF; HDAC2+FOXA2, HF and triple knockdown MYB+HDAC2+FOXA2, MHF).

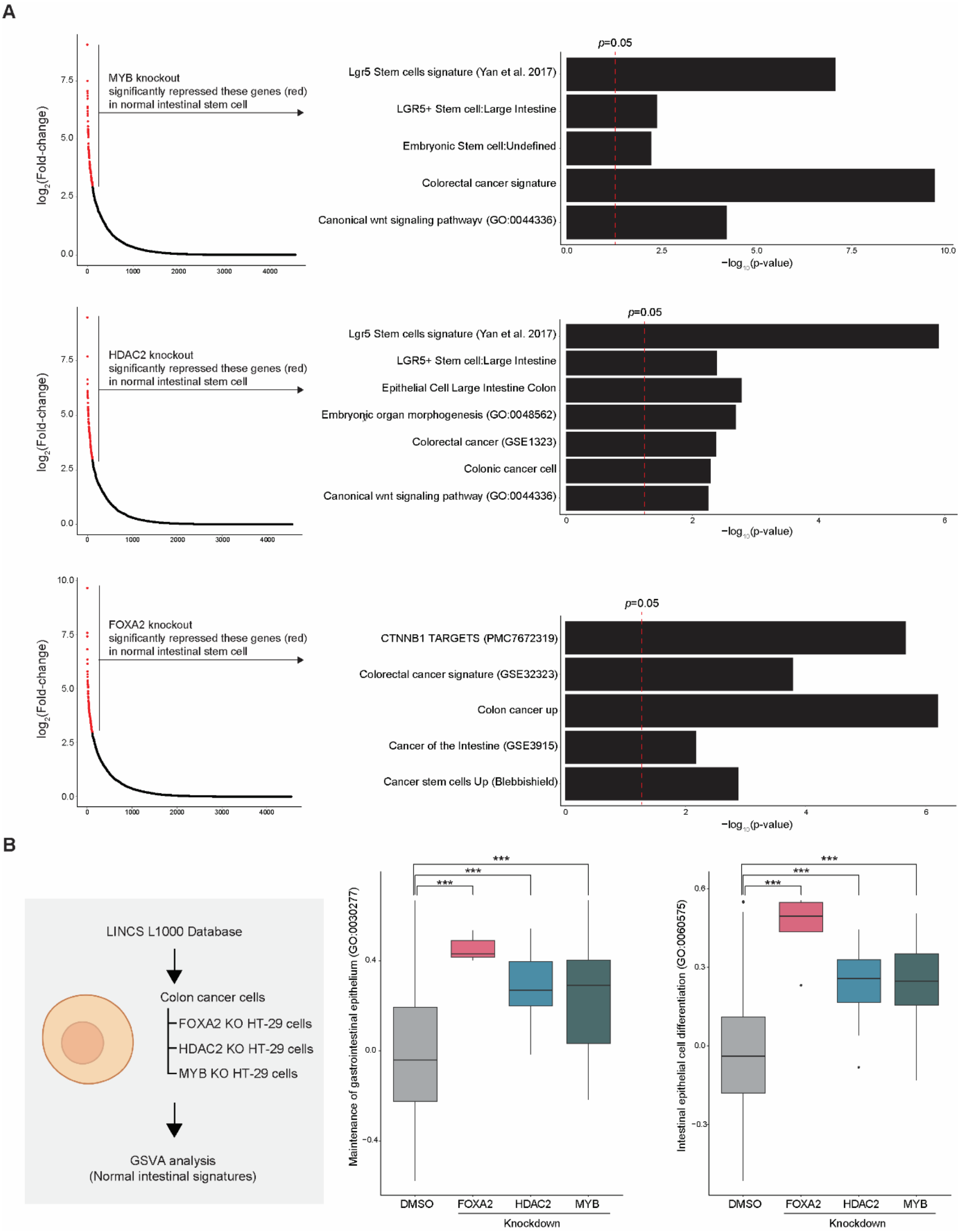

Figure S11. Impacts of control targets on normal enterocytes and colon cancer cell lines
(A) To determine the effects of three control targets—MYB, HDAC2, and FOXA2—on normal enterocytes, scTenifoldKnk was utilized to simulate *in silico* perturbations of each target. In the Hockey plot, genes exhibiting significant expression reduction following the virtual knockdown of each control target were indicated with red dots. Gene ontology analysis of these genes indicated that the three control targets are involved in regulating both LGR5+ large intestinal stem cell signatures and canonical Wnt pathway signatures. Notably, the canonical Wnt pathway plays a crucial role in maintaining

LGR5+ intestinal stem cells. This finding elucidates the mechanism by which knocking down these control targets triggers the differentiation of intestinal stem cells into enterocytes.
(B) Schematic diagrams illustrate the workflow for data processing from the LINCS L1000 database, aiming to assess whether the knockdown of three control targets in a colon cancer cell line can reversibly convert cancer cells to normal cells (left panel). The boxplots illustrated elevated signatures of normal intestinal epithelial cells after the knockdown of each control target compared to the DMSO treatment.

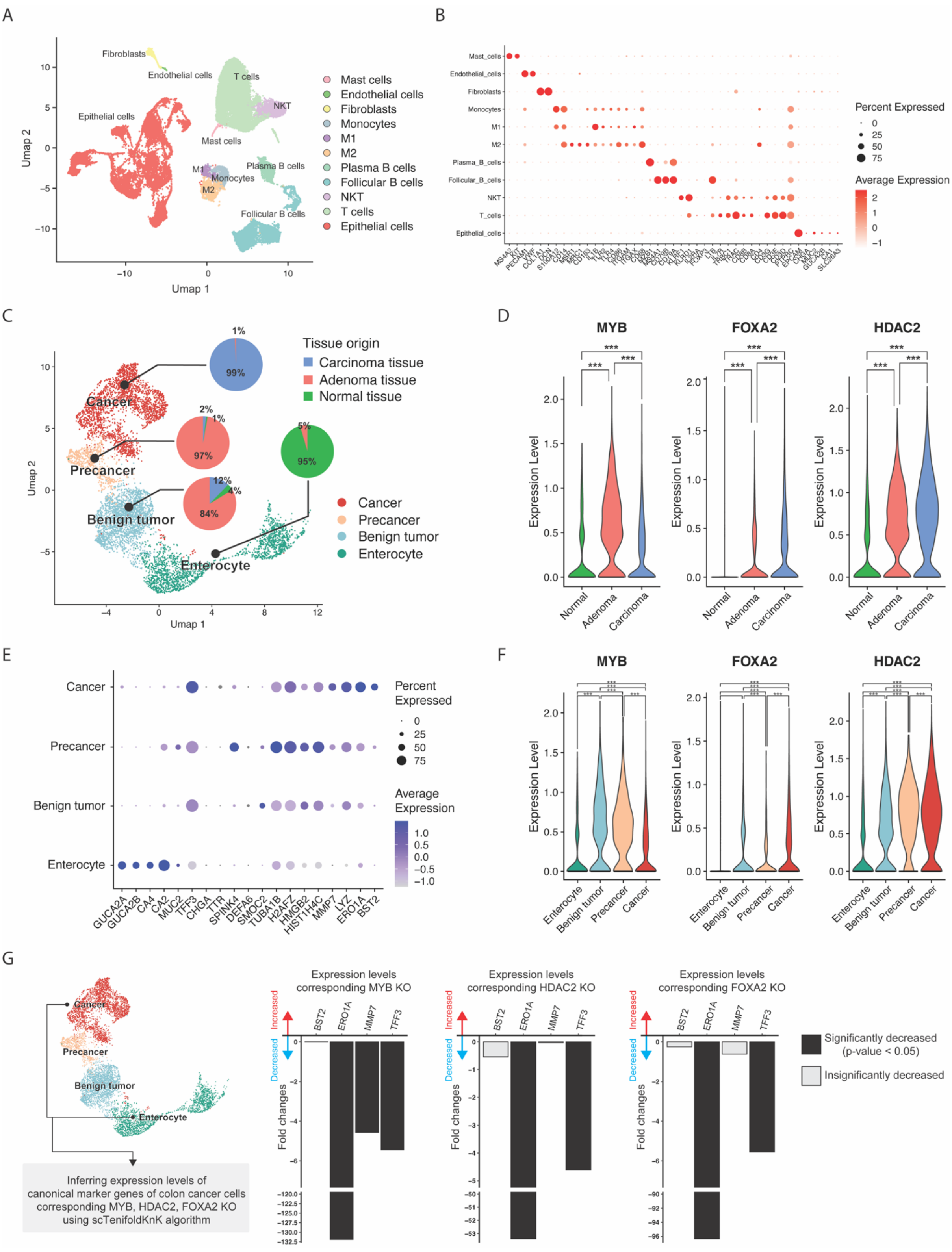

Figure S12. Expression levels of MYB, HDAC2, and FOXA2 and canonical marker genes in colon cancer cells during tumorigenesis.

(A) Cell type annotation of single-cell RNA-seq data consisting of normal colon tissues, adenoma tissues, and carcinoma tissues. A uniform manifold approximation and projection (UMAP) plot of the

cells obtained from four patients (12 samples) is shown; different colors represent different cell types.
(B) Expression levels of canonical marker genes for 11 cell types.
(C) Annotations of colon cancer, colon adenoma, and normal enterocytes among the enterocyte cells isolated in (A).
(D) Gene expression levels of the control targets in colon cancer, colon adenoma, and normal enterocytes.
(E) Violin plots showing the expression levels of canonical marker genes of colon cancer, colon adenoma, and normal enterocytes.
(F) Violin plots showing the expression levels of MYB, HDAC2, and FOXA2 in enterocytes, benign tumor cells, precancer cells, and cancer cells.
(G) Bar plots showing that the expression levels of canonical marker genes in colon cancer cells are significantly decreased following *in silico* knockout of MYB, HDAC2, and FOXA2, as inferred by the scTenifoldKnK algorithm (Wilcoxon rank-sum test: *** $p < 0.001$).

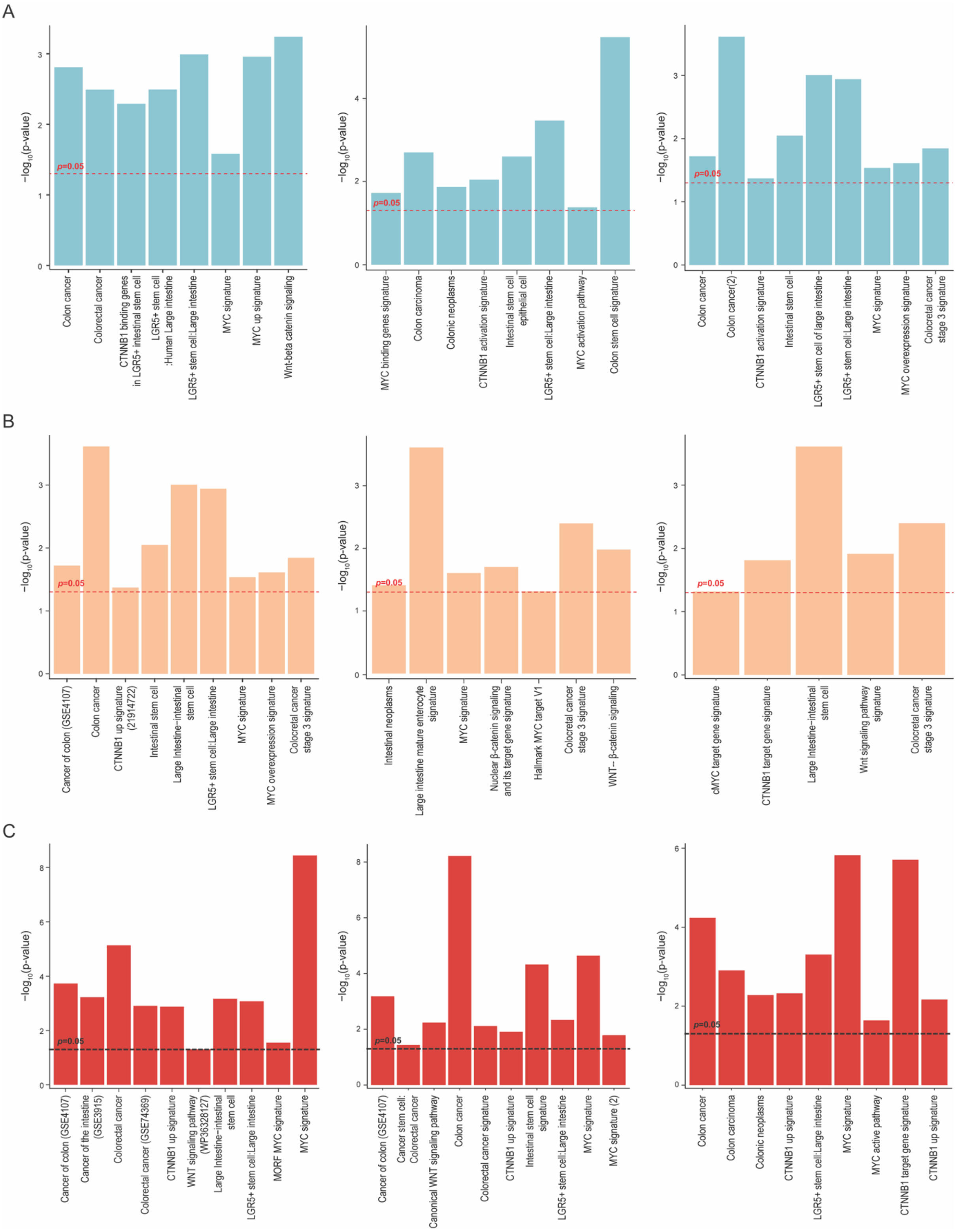

Figure S13. GO analysis of genes affected by *in silico* knockout of MYB, FOXA2 and HDAC2 in benign tumor cells, pre-cancer cells, and cancer cells

The results are presented in three groups: benign tumor cells (A), pre-cancerous cells (B), and cancer cells (C). In each group, the analysis results from knockout of MYB (left), HDAC2 (middle), and FOXA2 (right) are presented.

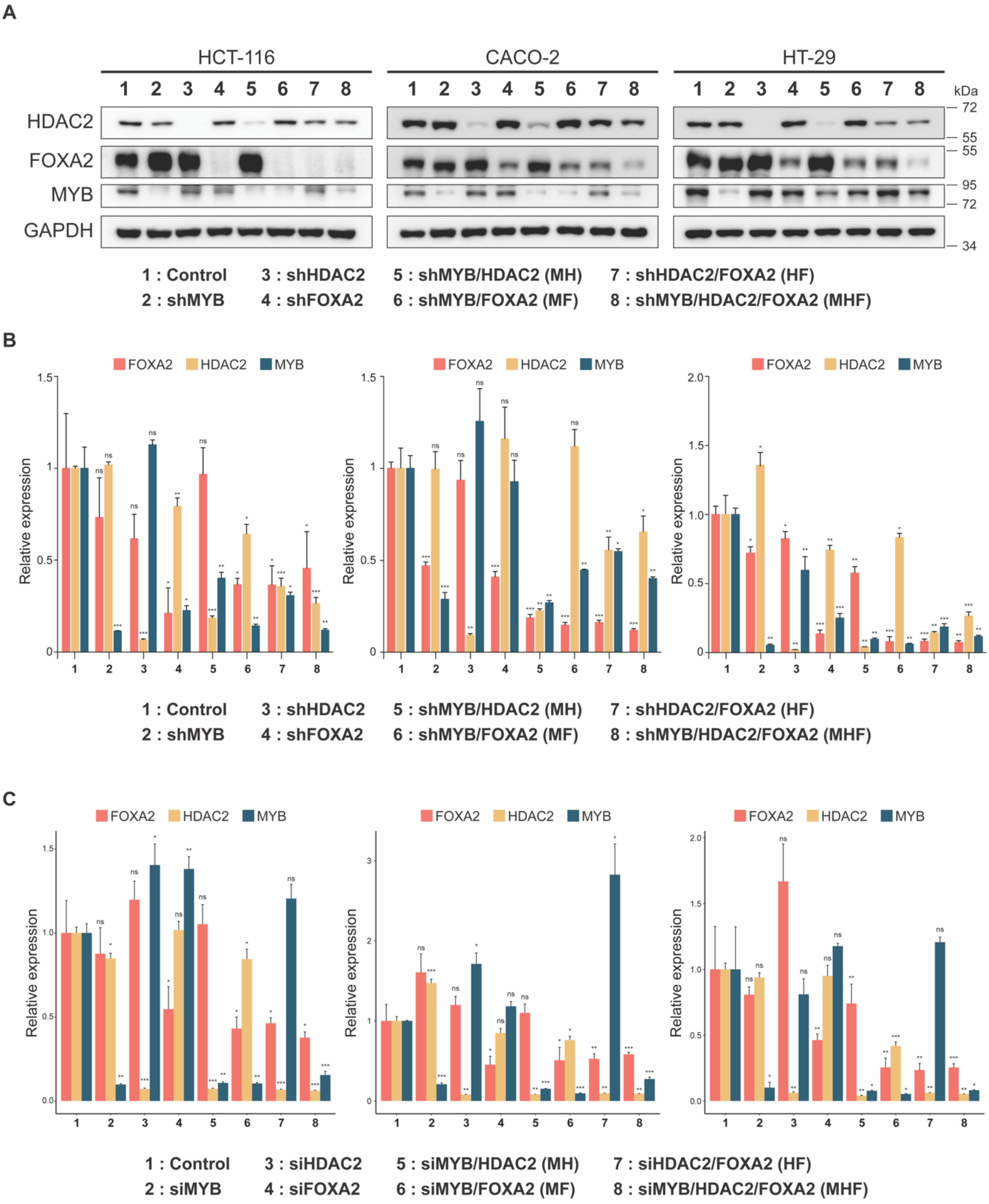

Figure S14. The protein and mRNA expression levels for combinatorial knockdown of MYB, HDAC2, and FOXA2 were measured by western blot analysis and qRT-PCR (siRNA and shRNA) (A-C) After knockdown of MYB, HDAC2, and FOXA2, their protein (A) and mRNA (B and C) expression levels were significantly decreased in HCT-116, CACO-2, and HT-29 cells.

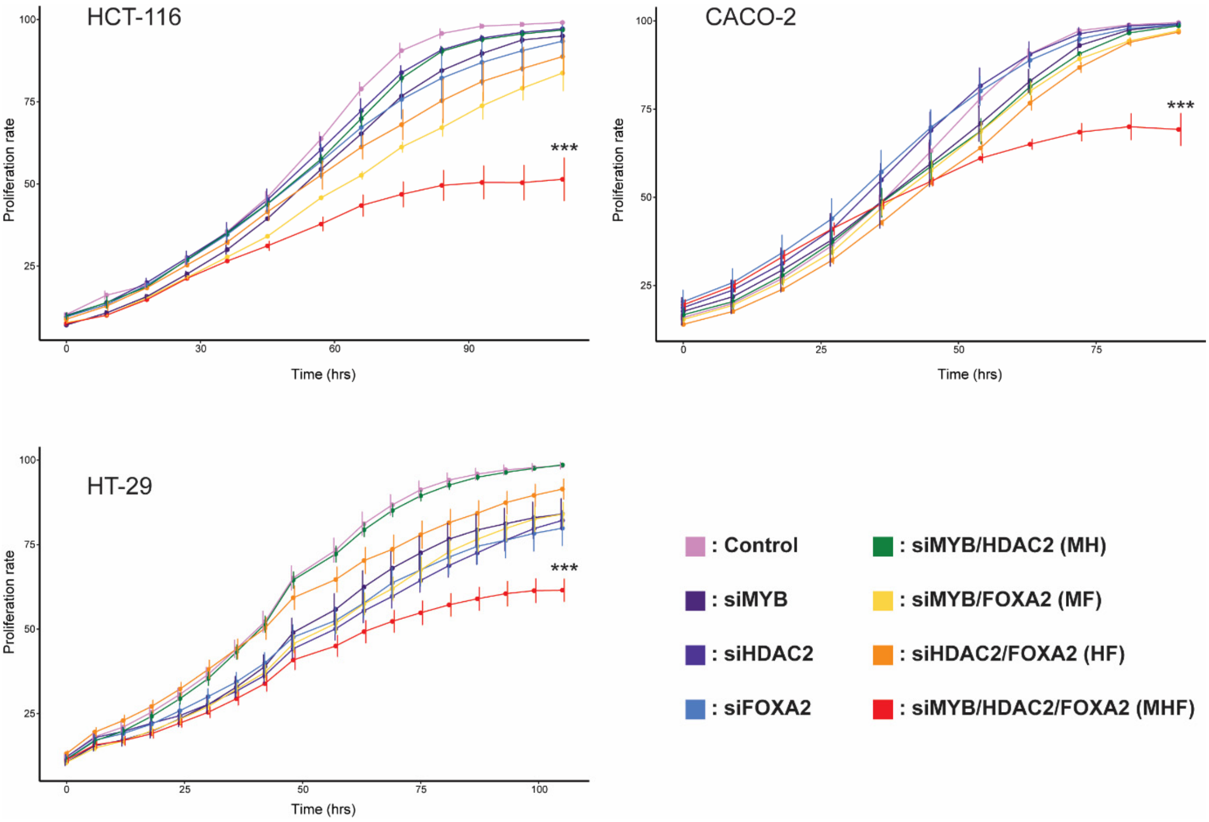

Figure S15. Growth curves of three colon cancer cell lines after suppression of three control targets using siRNA

The growth curves of colorectal cancer cells (HCT-116, CACO-2, and HT-29) after knockdown of control targets using siRNA (HCT-116: top left, CACO-2: top right, HT-29: bottom left). Cell growth rate was analyzed by IncuCyte. Data are presented as the mean ± SEM; n = 3 replicates (two tailed t-test: *** $p < 0.001$).

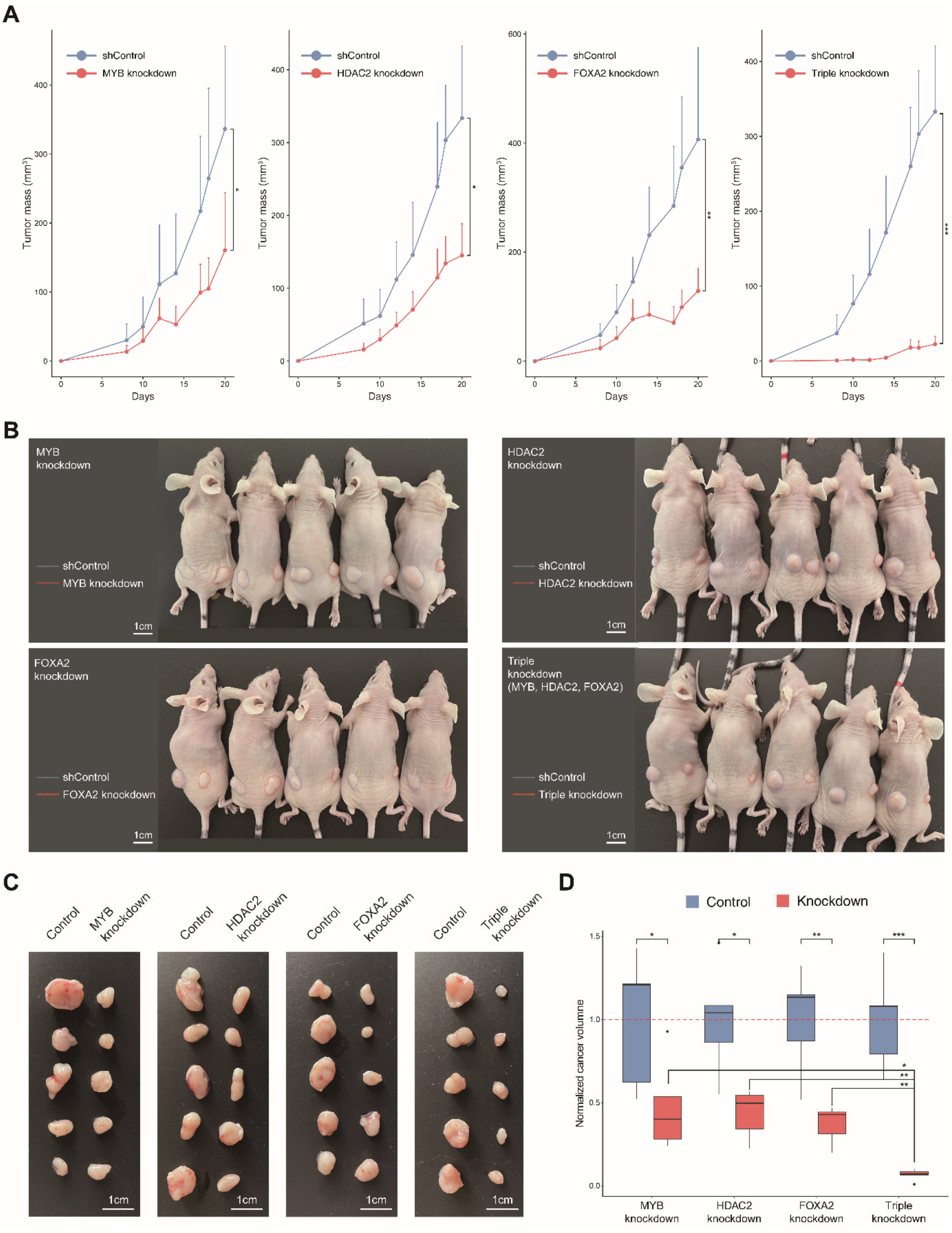

Figure S16. The simultaneous knockdown of the three targets more effectively reduces proliferation than single target knockdown *in vivo*

(A-D) HT-29 cells were injected to female athymic nude mice (Foxn1$^{nu/nu}$) and the proliferation of cancer cells was observed in tumor-bearing mice. Changes in the volume of three colon tumors 20 days (A) Changes in the volume of three colon tumors 20 days after tumor injection.

(B) Photographs of tumor-bearing mice with scramble control tumor (left, blue dotted line) and knockdown tumor (right, orange dotted line) on day 20.
(C) Photographs of tumors resected after sacrifice on day 20.
(D) Normalized cancer volume of a resected tumor.
Data are presented as the mean ± SEM; n = 5 measurements (two-tailed t-test: *** $p < 0.001$).

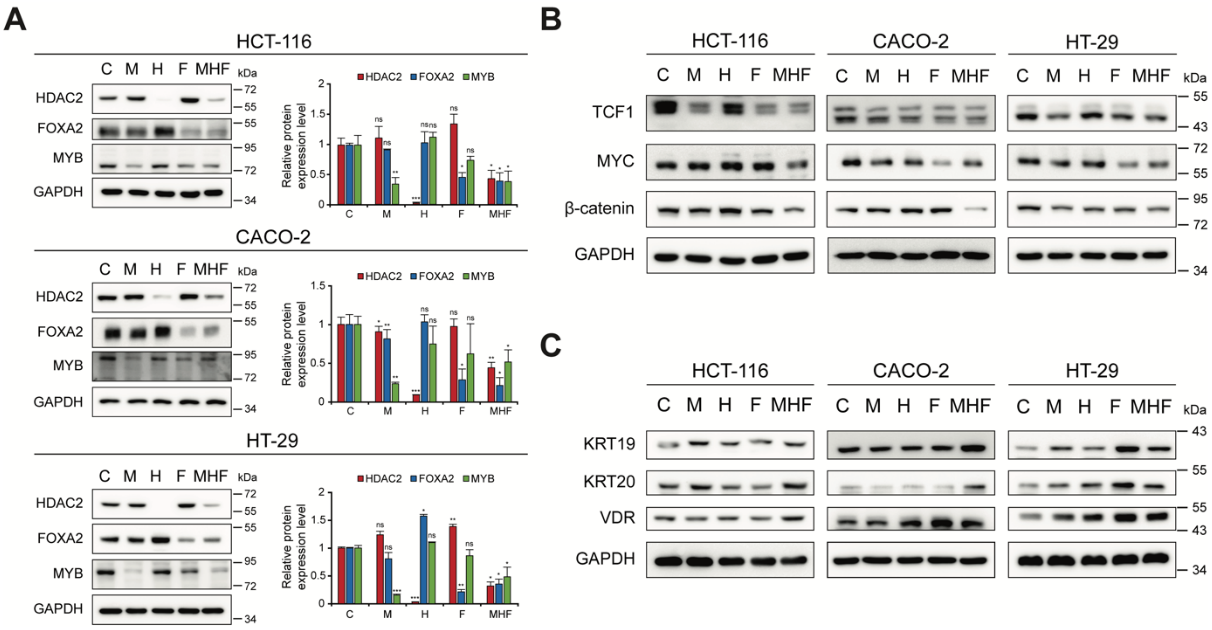

Figure S17. The simultaneous knockdown of the three targets effectively induced reverted colon cancer cells more efficiently than a single knockdown in three different colon cancer cell lines
(A) Protein expression levels of the three targets in three different colon cancer cell lines (scramble knockdown, C; single MYB knockdown, M; single HDAC2 knockdown, H; single FOXA2 knockdown, F; simultaneous MYB, HDAC2, and FOXA2 knockdown, MHF).
(B and C) Protein abundances were monitored by western blotting analysis of the representative genes of colonic enterocytes (KRT19, KRT20, and VDR) (B), MYC and WNT pathways (TCF1, MYC, and β-catenin) (C). (two tailed t-test: $* < 0.05$; $** \ p < 0.01$).

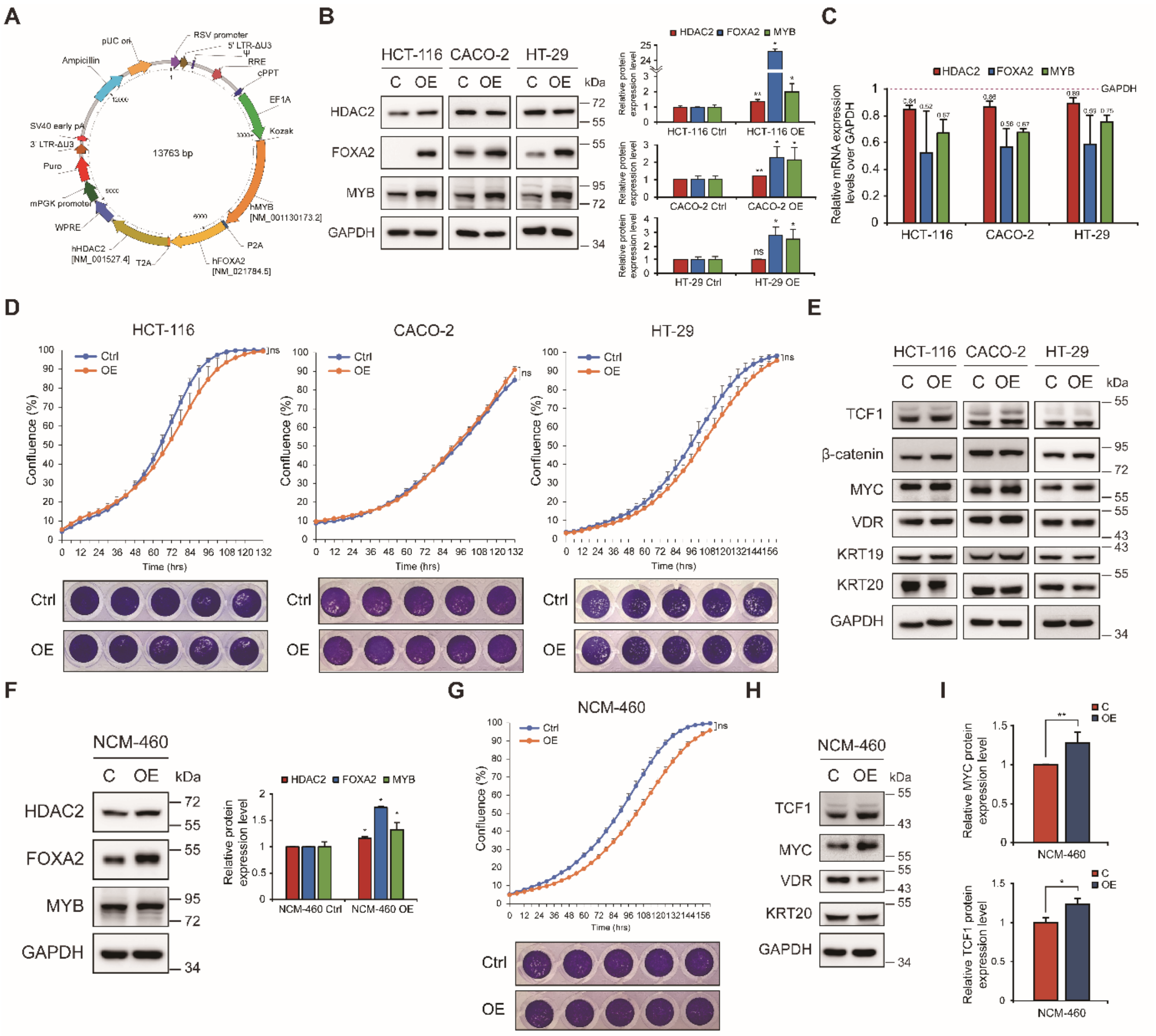

Figure S18. Overexpression of the three targets in the three colon cancer cells and a normal human colon epithelial cell line did not significantly impact their state
(A) Map of plasmid vector for MYB, HDAC2 and FOXA2 overexpression.
(B) Protein expression levels of the three targets in three colon cancer cells. (control, C; simultaneous overexpression of three targets, OE).
(C) Relative mRNA expression levels of MYB, HDAC2, and FOAX2 over GAPDH in three colon cancer cells. The value on top of each bar represents the relative mRNA expression level of each gene, with GAPDH expression set as 1.
(D) The growth curves of colorectal cancer cells (HCT-116, CACO-2, and HT-29) after overexpression of the targets (top). Cell growth rate was analyzed by IncuCyte. Representative images of crystal violet staining of cells (bottom).
(E) Protein abundances of the representative genes of MYC and WNT pathways and colonic enterocytes.
(F) Protein expression levels of the three targets in NCM-460 cells.
(G) The growth curves of normal colon epithelial cells (NCM-460) after overexpression of the targets (top). Cell growth rate was analyzed by IncuCyte. Representative images of crystal violet staining of cells (bottom).
(H) Protein expression levels of the representative genes (TCF1 and MYC) of MYC and WNT pathways and colonic enterocytes (VDR and KRT20).
(I) Relative protein expression levels of TCF1 and MYC in NCM-460 and NCM-460 with overexpression of the three targets. Data are presented as the mean ± SEM; n = 3 replicates (two tailed t-test: *<0.05; ** $p < 0.01$). We found that high basal expression level of MYB, HDAC2, and FOXA2

in colon cancer cells has been sufficiently contributing to sustaining the cancerous state, limiting further malignant effects upon overexpression, while overexpression in normal stem-cell like colon cell line helps maintain their stem-cell like state without increasing oncogenicity.

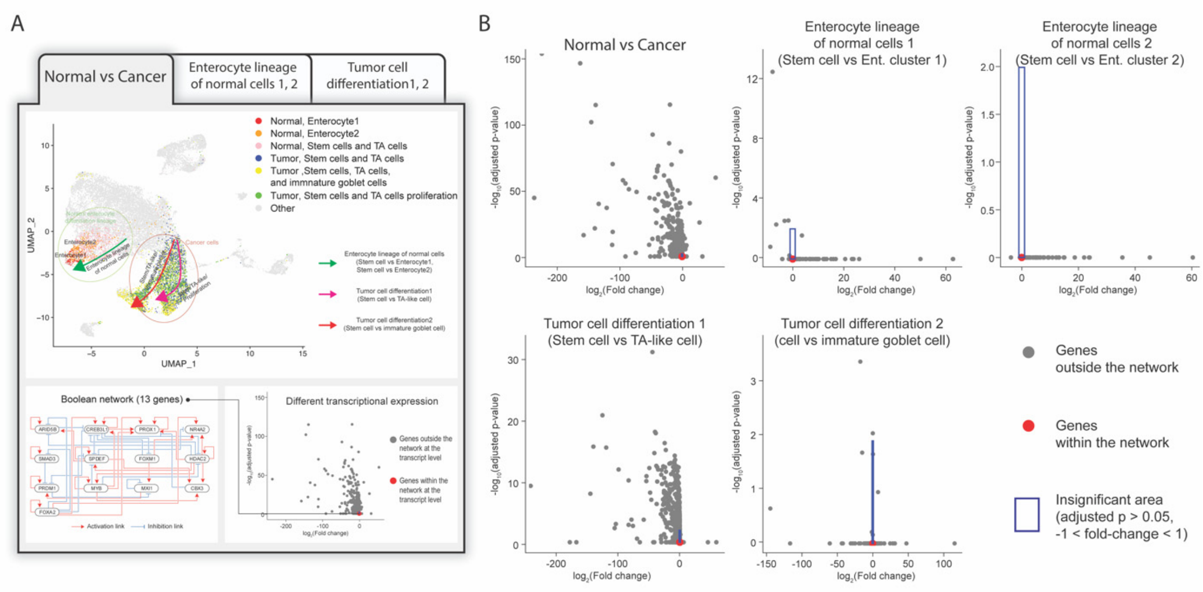

Figure S19. The long-read single-cell RNA analysis reveals that 13 TFs which are included in our Boolean network model exhibit no significant isoform alterations

(A) Classification of long-read RNA-seq, from matched normal colon and colon cancer cells, accompanied by a schematic illustration of the analytical process.

(B) For each group, the 13 TFs included in the Boolean network model demonstrate no significant isoform change: 1) Normal intestine and colon cancer, 2) Differentiation of enterocytes 1 (cE04), 3) Differentiation of enterocytes 2 (cE05), 4) Differentiation of colon cancer stem cells into TA-like cells, 5) Differentiation of colon cancer stem cells into immature goblet cells.

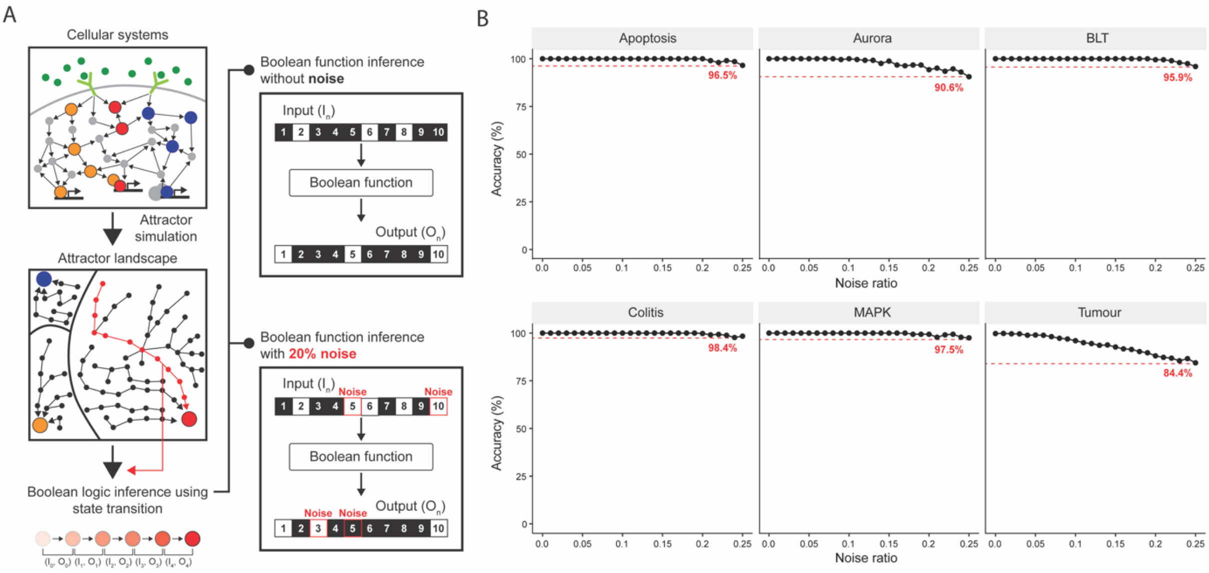

Figure S20. Robustness of the BENEIN framework to noise
(A) Schematic illustration of the methodology employed to analyze the resilience of the framework to noise.
(B) Dot plots depict the Boolean functions inferred by the BENEIN framework from the Apoptosis, Aurora, BLT, Colitis, MAPK, and Tumour Boolean networks from CellCollective (Apoptosis: Apoptosis network, Aurora: Aurora kinase A in neuroblastoma network, BLT: Basal-to-luminal A transition network, Colitis: Colitis associated colon cancer network, MAPK: MAPK network, and Tumour: Tumour cell invasion and migration network) from CellCollective.

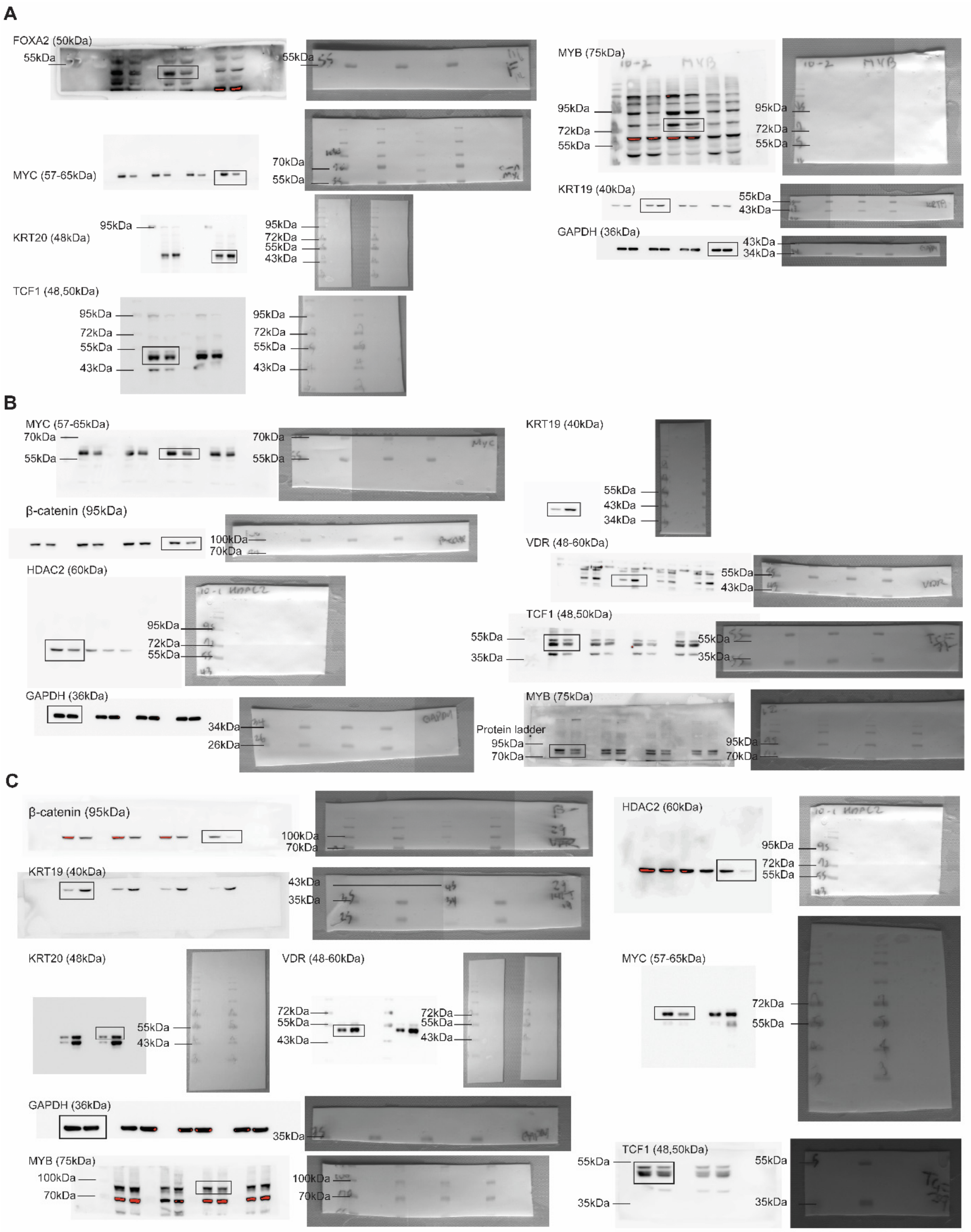

Figure S21. Uncropped images of western blots analysis corresponding to the indicated blots in this study
(A-C) Full blots of western blot analysis using HCT-116 cells (A), CACO-2 (B), and HT-29 (C) in this study.

	SCNS	Hamey *et al.* 2017	IQCELL	BENEIN
Maximum in-degree for logic inference	8	8	6	13
Logic form constraints	4 activators and 4 repressors	4 activators and 4 repressors	4 activators and 2 repressors	No constraints
Feedback Loop Inference	○	○	×	○
Equidistance assurance*	×	×	×	○

* Ensuring regular time point intervals in inferring the regulation logics of Boolean network models

Table S1. Studies for Boolean network inference regarding cell differentiation
The Single-Cell Network Synthesis (SCNS), Hamey *et al.,* and the Integrated Platform for Predicting the Qualitative Effects of Gene Perturbations on Developmental Trajectories (IQCELL) are representative studies for Boolean network model reconstruction using single-cell data.

Table S8. List of primers used in this study.

Target gene	Forward primer sequence (5'-3')	Reverse primer sequence (5'-3')
ARID5B	AAGGTTGCCATTGGTGAAGAGTGC	GACGGCGGGCTGTTATTGTTTCAT
CBX3	TGGCCTCCAACAAAACTACA	TCCCATTCACTACACGTCGA
CREB3L1	GGAGAATGCCAACAGGACC	GCACCAGAACAAAGCACAAG
CTNNB1	CACAAGCAGAGTGCTGAAGGTG	GATTCCTGAGAGTCCAAAGACAG
FOXA2	AACGACTGTTTCCTGAAGGT	CTGCTTCTCGCACTTGAAGC
FOXM1	CCTTCTGGACCATTCACCCC	TCACCGGGAACTGGATAGGT
GAPDH	TGATGACATCAAGAAGGTGGTGAAG	TCCTTGGAGGCCATGTGGGCCAT
HDAC2	GAGGTGGCTACACAATCCGTA	ACACCAGGTGCATGAGGTAAC
KRT19	GCGAGCTAGAGGTGAAGATC	CGGAAGTCATCTGCAGCCA
KRT20	ACGCCAGAACAACGAATACC	ACGACCTTGCCATCCACTAC
MXI1	CAACGTGCAGCGTCTGCTGGAGGC	CGATTCTTTTCCAGCTCATTGTG
MYB	GGGAACAGATGGGCAGAAATCG	GCTGGCTTTTGAAGACTCCTGC
MYC	GGCTCCTGGCAAAAGGTCA	CTGCGTAGTTGTGCTGATGT
PROX1	TCACCTTATTCGGGAAGTGC	GTACTGGTGACCCCATCGTT
SMAD3	CATCGAGCCCCAGAGCAATA	GTGGTTCATCTGGTGGTCACT
TCF7	GACATCAGCCAGAAGCAAG	CACCAGAACCTAGCATCAAG
VDR	AGATGACCCTTCTGTGACCCTA	ATGGCACTTGACTTCAGCAGTA

Table S9. List of shRNA sequences used in this study.

Name	Target sequence	Catalog Number
shMYB	CCGGGCTATCAAGAACCACTGGAATCTC GAGATTCCAGTGGTTCTTGATAGCTTTTTG	TRCN0000288601
shHDAC2	CCGGCAGTCTCACCAATTTCAGAAACTC GAGTTTCTGAAATTGGTGAGACTGTTTTT	TRCN0000004819
shFOXA2	CCGGGGAACACCACTACGCCTTCAACTC GAGTTGAAGGCGTAGTGGTGTTCCTTTTTG	TRCN0000329716
