# Supplementary material for: Control of Cellular Differentiation Trajectories for Cancer Reversion
Source: Adv Sci (Weinh). 2024 Dec 11;12(3):2402132. doi: 10.1002/advs.202402132 (PMC11744559; doi:10.1002/advs.202402132)
Supplement: Supplementary file 2 — Supporting Information [file ADVS-12-2402132-s003.docx]

Supplementary Note 1

Control of cellular differentiation trajectories for cancer reversion

*Jeong-Ryeol Gong, Chun-Kyung Lee, Hoon-Min Kim, Juhee Kim, Jaeog Jeon, Sunmin Park, and Kwang-Hyun Cho**

**This file includes:**

**Supplementary Note 1.**

**Comprehensive analysis including the structure and dynamics of the reconstructed Boolean GRN model, the importance of the control targets, and challenges in logic inference.**

**Degree distribution of the reconstructed Boolean GRN**

As it is well-known that transcription regulatory networks are generally scale-free [1], we examined whether the Boolean GRN reconstructed by BENEIN also shows such a scale-free property. A fundamental feature of a scale-free network lies in its degree distribution, which adheres to a power-law distribution. This entails the presence of a hub node, characterized by a limited number of nodes exhibiting significantly higher connectivity in comparison to other nodes. Such a network structure facilitates efficient information propagation and enhances network stability. Unfortunately, the number of nodes (13) in the reconstructed Boolean GRN is relatively small, so we cannot draw any definite conclusion from the degree distribution. However, we found that the degree distribution is not uniform and has a shape similar to the power-law distribution with a long tail, which means that the network has a large number of nodes with low connectivity and a small number of nodes with high connectivity (Supplementary Note Figure 1).


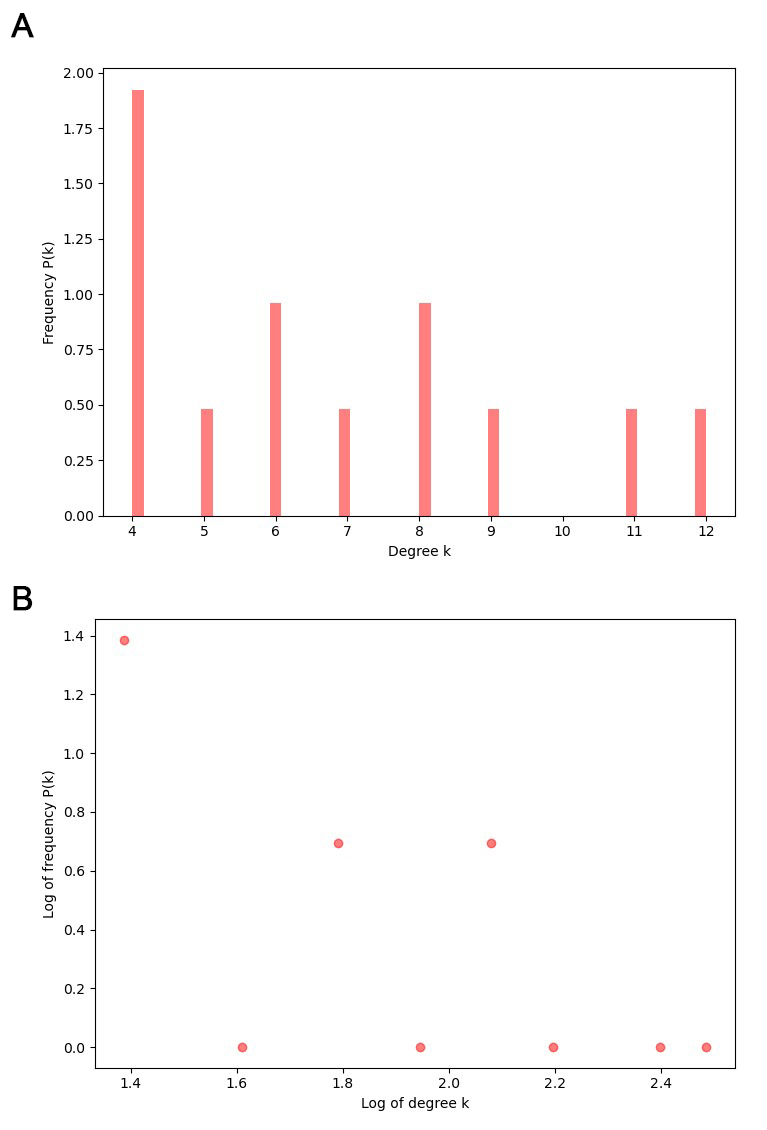


**Supplementary Note Figure 1.** The degree distribution of the reconstructed Boolean GRN by BENEIN is similar to the power-law distribution

(A) Degree distribution without log transformation.

(B) Degree distribution with log transformation.

**The average shortest path length and average clustering coefficient of the reconstructed Boolean GRN**

The average shortest path length is an important characteristic on network structure that provides various insights [2]. A shorter average shortest path length indicates that nodes within the network are more closely connected, allowing for faster information propagation. Therefore, a network with a shorter average shortest path length operates more efficiently. The reconstructed Boolean GRNs have a much shorter average shortest path length than the randomly rewired 1,000 Boolean networks with the same number of nodes and links, suggesting that the reconstructed GRNs have structural characteristics of fast information propagation (Supplementary Note Figure 2).


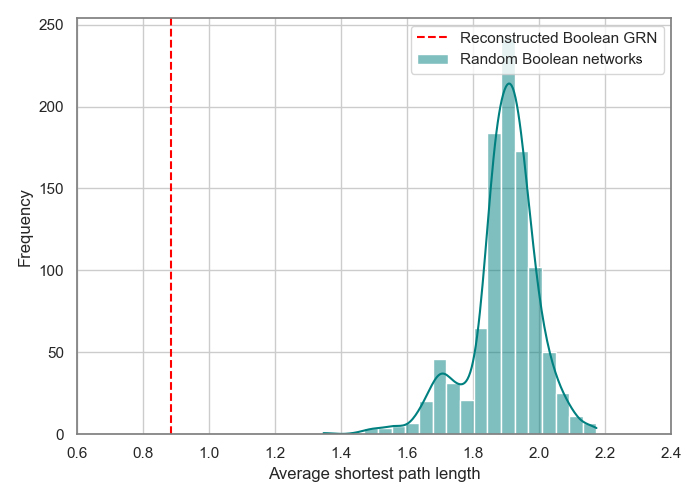
**Supplementary Note Figure 2.** Comparison of the average shortest path length of the reconstructed Boolean GRN by BENEIN and the randomly rewired 1,000 Boolean networks with the same number of nodes and links.

The average clustering coefficient of a network is an important indicator showing how closely the nodes are interconnected within the network [2]. A network with a low average clustering coefficient indicates that there are fewer local connections between nodes. This suggests that small groups or clusters do not form within the network, and that the nodes have relatively even interconnections. The average clustering coefficient of the reconstructed Boolean GRN is slightly lower than that of 1,000 random Boolean networks with the same number of nodes and links rewired. This means that the nodes in the reconstructed Boolean GRN exhibit relatively little clustering (Supplementary Note Figure 3).


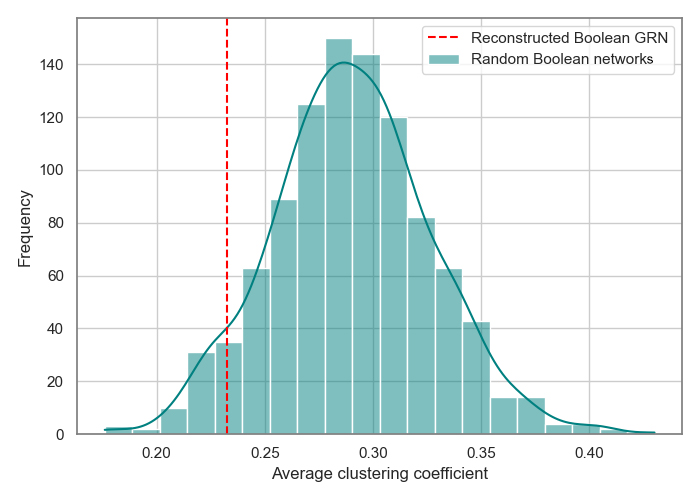
**Supplementary Note Figure 3.** Comparison of the average clustering coefficient of the reconstructed Boolean GRN by BENEIN and that of the randomly rewired 1,000 Boolean networks with the same number of nodes and links.

**Importance analysis of each node using a machine learning model**

In the main text, we described the results of a weighted outdegree centrality analysis based on the effectiveness obtained for edges, which can be considered indicative of their influence. To further enhance our understanding of such node-specific influences, we employed a random forest regression model and calculated and compared the importance of each node based on the training results of the model.

To reverse-generate the data for training from the reconstructed Boolean GRN model, we performed a perturbation simulation, in which we perturbed all possible combinations of 1-3 nodes, and calculated the average activity based on the attractor information obtained. Therefore, the random forest regression model learns the information of the perturbed nodes and the average activity accordingly. Then, the importance was obtained from the learned random forest regression model to rank the nodes that have the greatest influence on each node. As a result, a rank-sum analysis was conducted, revealing that FOXA2, MYB, and HDAC2 ranked the lowest at 25, 40, and 47, respectively. This finding reaffirms the notion that MYB, HDAC2, and FOXA2 exert the most substantial influence in the reconstructed Boolean GRN model (Supplementary Note Figure 4).


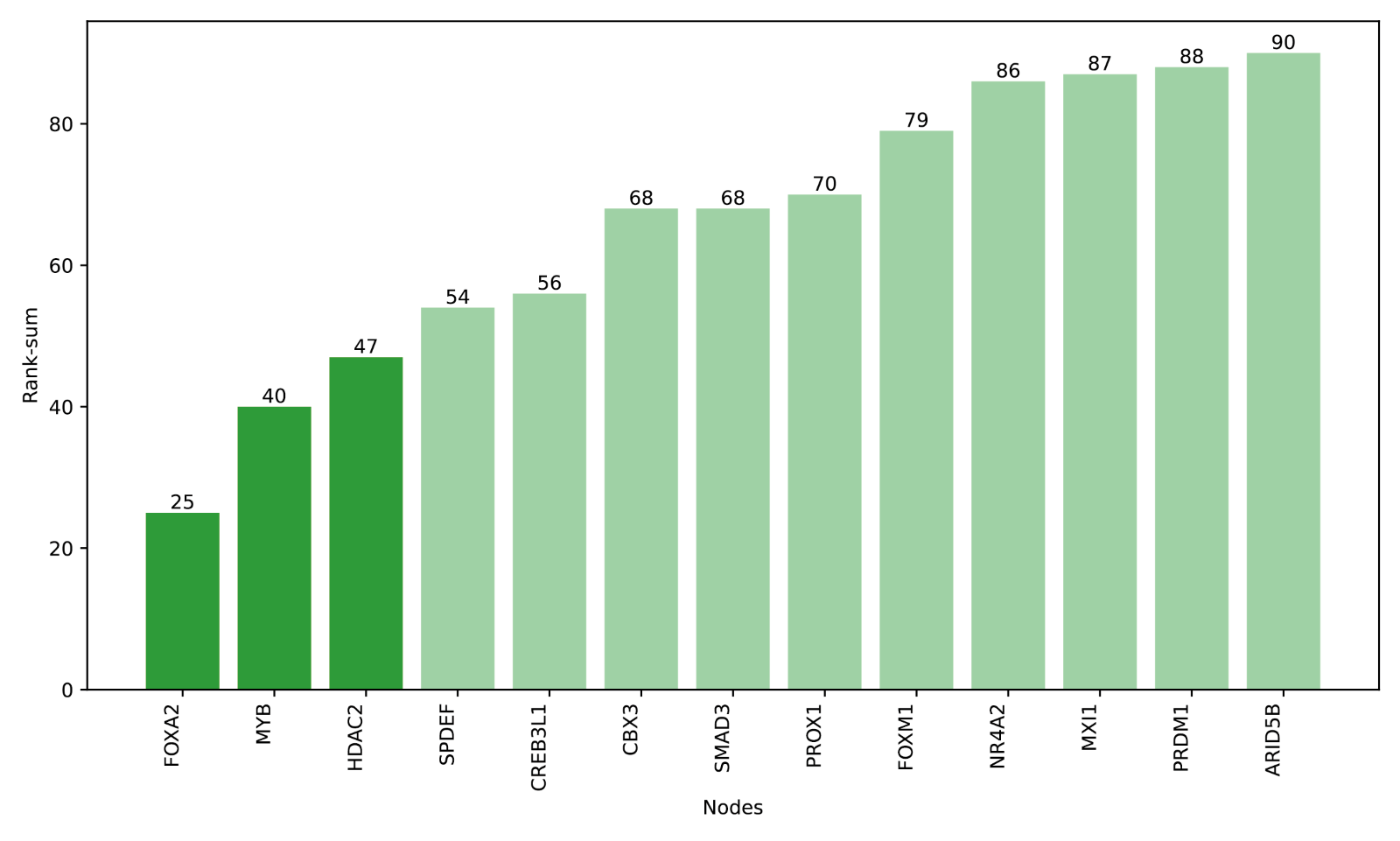
**Supplementary Note Figure 4.** A bar chart displaying the results of the rank-sum analysis is presented. The dark green bars represent FOXA2, MYB, and HDAC2, which exhibited the lowest ranks.

Impact of both upregulated and downregulated regulatory elements

We conducted a comprehensive perturbation analysis to assess the impact of both upregulated and downregulated regulatory elements on the differentiation process within the Boolean GRN model. In particular, we performed 268 perturbation simulations on every possible three node combination within the Boolean GRN model. The most effective outcome, with a maximum cosine similarity of 0.968 between the average activity of the perturbed GRN model and the FD enterocyte state, was achieved by the simultaneous knockdown of MYB, HDAC2, and FOXA2 as identified in this study (Supplementary Note Figure 5).


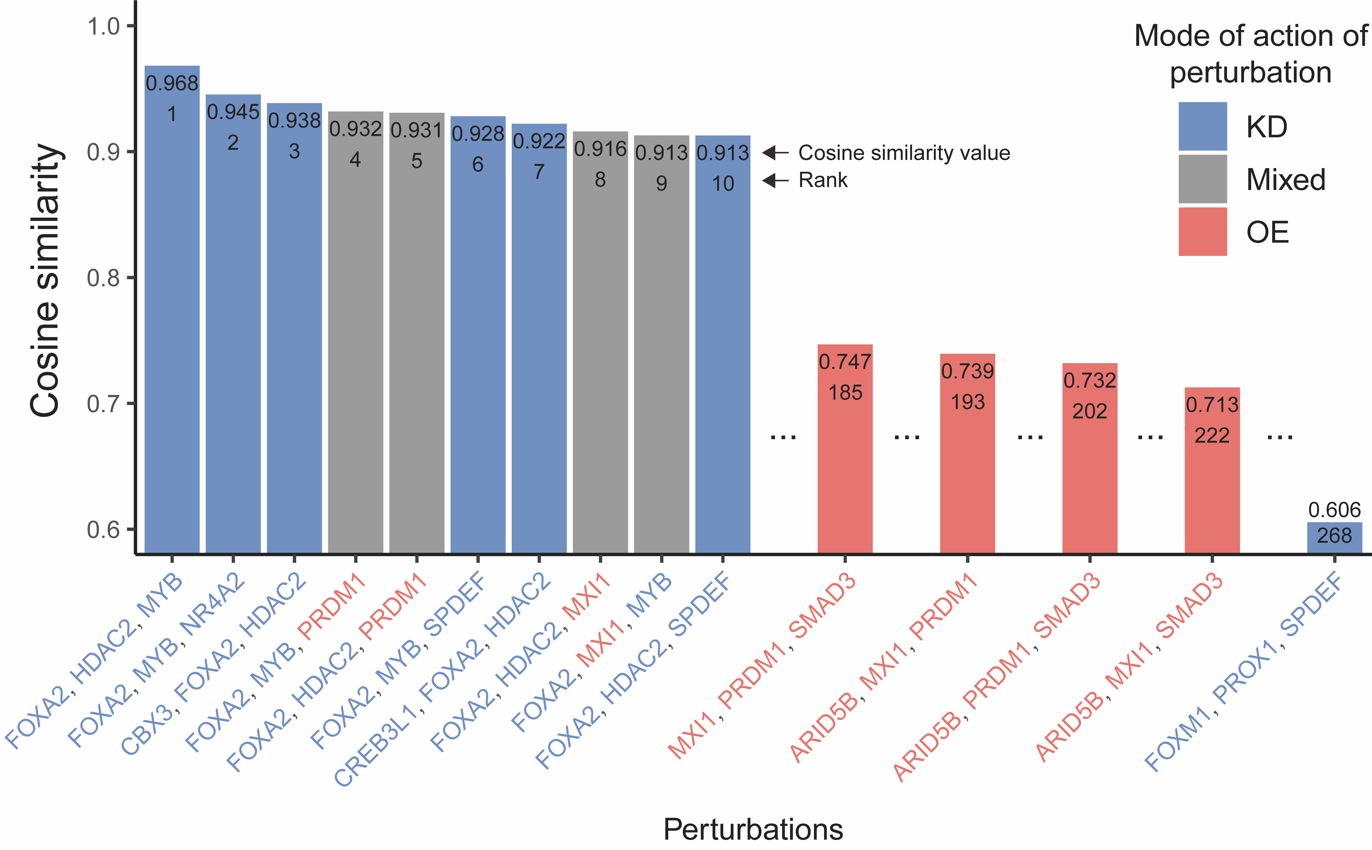


**Supplementary Note Figure 5**. Bar plot of cosine similarities between the node activity of the perturbed network and the desired attractor for every three-node combination of perturbations. The value on top of each bar is a cosine similarity value, and the value on the second line represents rank among every combination. The color of the text for each target is marked blue for knockdown, and red for overexpression.

**Challenges in logic inference along the cell trajectories with inconsistent spacing between single cells during cellular differentiation**

Cellular differentiation is a nonlinear dynamic process. Hence, time-series gene expression profiles are critical to investigate the underlying dynamic regulation. Pseudotime cell trajectories inferred from single-cell transcriptome data can be used for such a purpose, whereas the direct measurement of time-series gene expression profiles remains impractical, if not impossible. However, because the distance between cell state vectors is uneven in practice, pseudotime inference algorithms that infer trajectories based on the similarity of gene expression patterns inevitably have challenges caused by such uneven time intervals between cells along the trajectory. Therefore, pseudotime inference algorithms are not appropriate for investigating cellular dynamics.

To highlight the aforementioned limitation, we designed numerical experiments for which we generated simulated data from six existing biological Boolean network models (Supplementary Note Table 1) and inferred the Boolean logic from the data by reverse-engineering. Two distinct sets of simulated datasets were generated. In the first dataset, an equidistance was maintained between the current and the next state vectors. In contrast, the second dataset has heterogeneous distances with random intervals. Both datasets were structured to include a minimum of 100 data points and incrementally augmented by 100 data points, up to a maximum of 2,000, to facilitate comprehensive simulation analysis.

Utilizing the Quine-McCluskey algorithm, Boolean logic was inferred from each dataset. As a result, when inferring Boolean logic from the dataset with an equidistance, we obtained an average accuracy of approximately 6% higher compared to the Boolean logic inferred from the dataset with heterogeneous distances (Supplementary Note Figure 6). In particular, the Boolean logic from the dataset with an equidistance distance showed an accuracy close to 100% when the number of data points exceeded 1,500 (Supplementary Note Figure 7).

These findings imply that maintaining consistency in the data interval between the current and the next state significantly contributes to improving the accuracy of logic inference.

**Supplementary Note Table 1**. Structural characteristics of the six Boolean network models that were employed to generate the simulated data (Apoptosis; Apoptosis network [3], Aurora; Aurora kinase A in neuroblastoma network [4], BLT; Basal-to-luminal A transition network [5], Colitis; Colitis associated colon cancer network [6], MAPK; MAPK network [7], and Tumour; Tumour cell invasion and migration network [8]).

| Biological Boolean model | Number of nodes | Total indegree | Average indegree | Maximum indegree |
| --- | --- | --- | --- | --- |
| Tumor | 32 | 158 | 4.9375 | 8 |
| MAPK | 53 | 108 | 2.037735849 | 5 |
| BLT | 30 | 67 | 2.233333333 | 5 |
| Aurora | 23 | 47 | 2.043478261 | 7 |
| Apoptosis | 41 | 75 | 1.829268293 | 4 |
| Colitis | 70 | 154 | 2.2 | 5 |


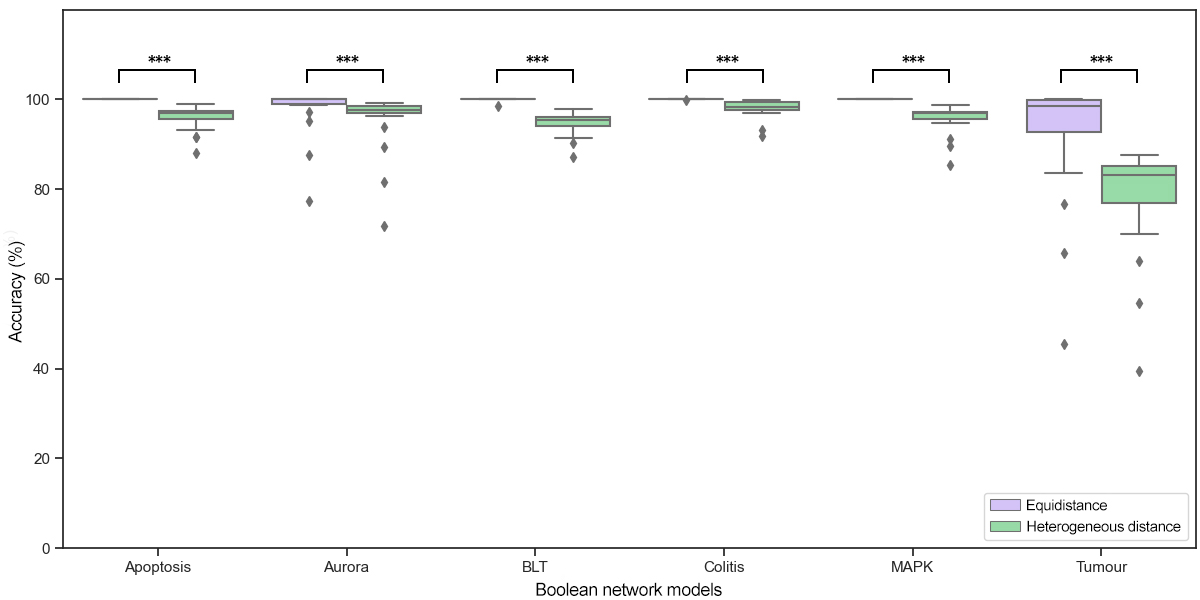
**Supplementary Note Figure 6.** Box plots comparing the accuracy of Boolean logic inferred from datasets with an equidistance and that with heterogeneous distances between current and next state vectors.


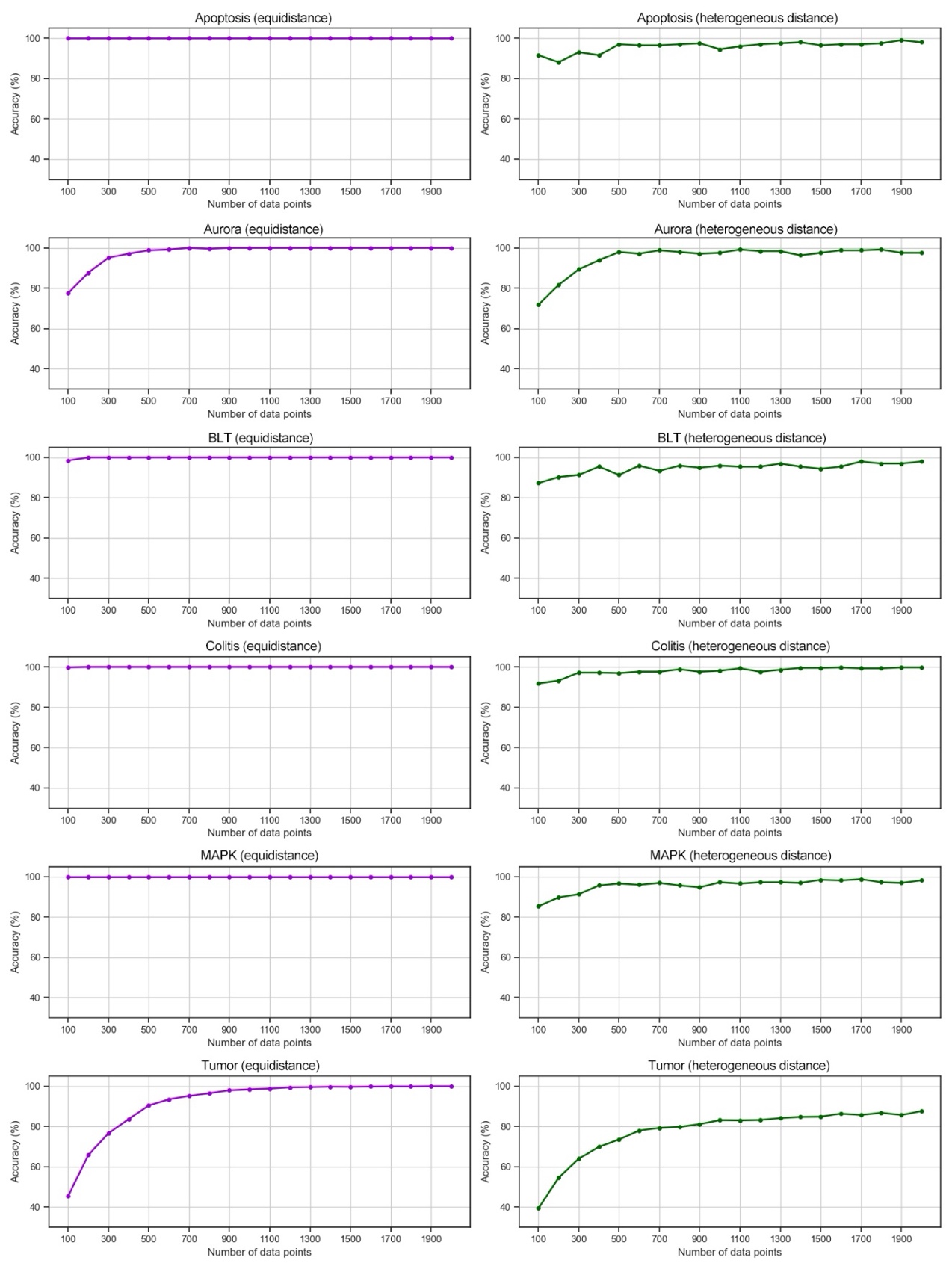


**Supplementary Note Figure 7.** Multiplot comparing the accuracy of Boolean logic inferred from datasets with an equidistance and that with heterogeneous distances between current and next state vectors.

**Simulation of alternative splicing effects on the identification of the transcription factors**

To address the impact of alternative splicing events on the identification of transcription factors (TFs) within the Boolean network model, we designed and performed a simulation on the count matrices to evaluate the potential effects of splicing variability. To our knowledge, directly obtaining or generating the isoform-level splicing data is not feasible due to the limitations inherent to short-read single-cell RNA sequencing. So, we have modeled alternative splicing events by adjusting the spliced (S) and unspliced (U) RNA counts for the 13 TFs involved in our network. In particular, we introduced an adjustment factor, referred to as alpha, which transfers some portion of the gene expression level from S to U, or vice versa. Then, by varying the value of alpha between -5% and 5%, we altered the S and U counts for the selected TFs while keeping the overall gene expression level constant. This adjustment allowed us to systematically vary the balance between spliced and unspliced counts, simulating splicing outcomes while preserving total expression. The changes in splicing balance were implemented uniformly across all 13 TFs. Then, the network structure construction was performed using the BENEIN workflow. We recalculated RNA velocity, calculated conditional mutual information, pruned the network with pySCENIC, and extracted the largest SCC to determine whether it contained the TFs from the Boolean network. The results show that up to two TFs (NR4A2 and PROX1) were not properly identified in the inferred network structure owing to the introduced noise. However, we found that these excluded TFs are output nodes within the Boolean network model, indicating that their absence does not actually affect the regulatory dynamics of the model, thus their exclusion does not significantly disrupt the system's behavior. Nevertheless, in general, these results implies that the noise caused by alternative splicing events may affect the identification of transcription factors, though the effect was minimal in the case of human colon enterocyte differentiation used in our study.


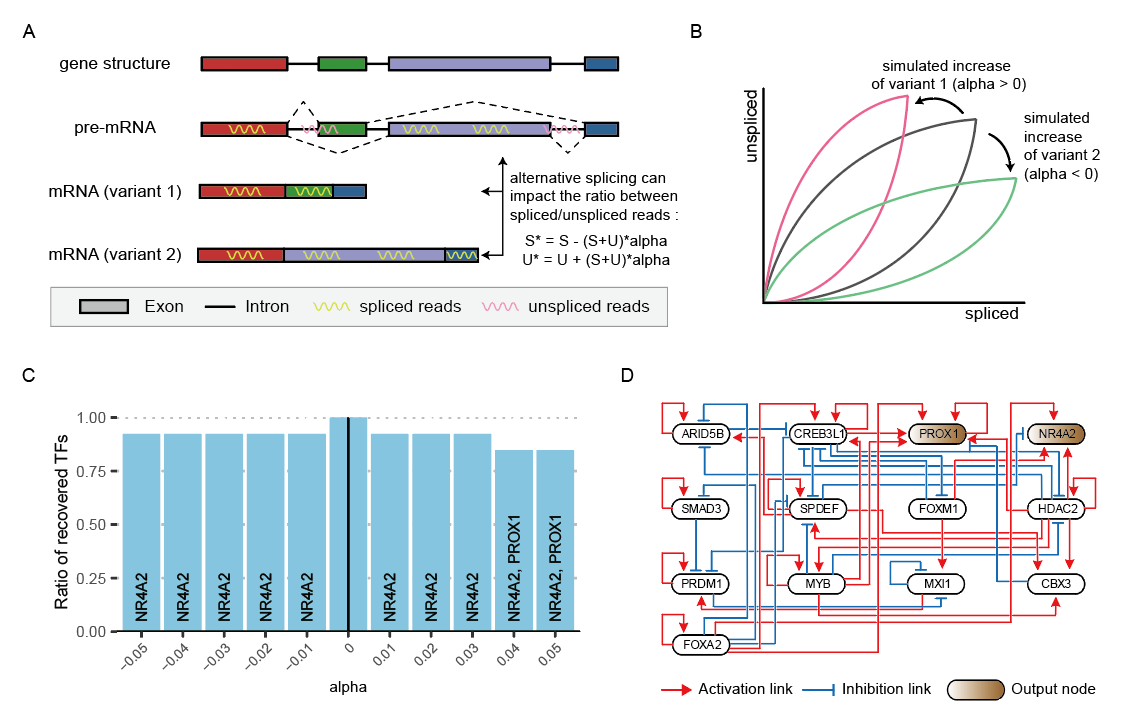


Supplementary Note Figure 8. Simulating the effects of alternative splicing events on the identification of TFs. (A) Schematic of alternative splicing affecting the spliced/unspliced RNA reads. (B) Schematic of the effects of simulated alternative splicing events on the phase portrait. (C) Bar plot of the ratio of recovered TFs over noise levels. TF(s) marked on the bar represents the TF(s) unidentified in the resulting network structure. (D) Boolean network model in the study with output nodes marked. The TFs frequently unidentified in (C) are the output nodes.

**Reference**

1. Barabasi, A.L. and Z.N. Oltvai, *Network biology: understanding the cell's functional organization.* Nat Rev Genet, 2004. **5**(2): p. 101-13.

2. Albert, R. and A.L. Barabási, *Statistical mechanics of complex networks.* Reviews of Modern Physics, 2002. **74**(1): p. 47-97.

3. Mai, Z.X. and H.Y. Liu, *Boolean network-based analysis of the apoptosis network: Irreversible apoptosis and stable surviving.* Journal of Theoretical Biology, 2009. **259**(4): p. 760-769.

4. Dahlhaus, M., et al., *Boolean modeling identifies Greatwall/MASTL as an important regulator in the AURKA network of neuroblastoma.* Cancer Lett, 2016. **371**(1): p. 79-89.

5. Choi, S.R., et al., *Network Analysis Identifies Regulators of Basal-Like Breast Cancer Reprogramming and Endocrine Therapy Vulnerability.* Cancer Research, 2022. **82**(2): p. 320-333.

6. Lu, J.Y., et al., *Network modelling reveals the mechanism underlying colitis-associated colon cancer and identifies novel combinatorial anti-cancer targets.* Scientific Reports, 2015. **5**.

7. Grieco, L., et al., *Integrative Modelling of the Influence of MAPK Network on Cancer Cell Fate Decision.* Plos Computational Biology, 2013. **9**(10).

8. Cohen, D.P.A., et al., *Mathematical Modelling of Molecular Pathways Enabling Tumour Cell Invasion and Migration.* Plos Computational Biology, 2015. **11**(11).
