# Supplementary material for: Control of Cellular Differentiation Trajectories for Cancer Reversion
Source: Adv Sci (Weinh). 2024 Dec 11;12(3):2402132. doi: 10.1002/advs.202402132 (PMC11744559; doi:10.1002/advs.202402132)
Supplement: Supplementary file 3 — Supporting Information [file ADVS-12-2402132-s004.pdf]

ADVANCED SCIENCE
Open Access

Supporting Information

for *Adv. Sci.*, DOI 10.1002/advs.202402132

Control of Cellular Differentiation Trajectories for Cancer Reversion

*Jeong-Ryeol Gong, Chun-Kyung Lee, Hoon-Min Kim, Juhee Kim, Jaeog Jeon, Sunmin Park and Kwang-Hyun Cho**

Supplementary Note 2

Control of cellular differentiation trajectories for cancer reversion

*Jeong-Ryeol Gong, Chun-Kyung Lee, Hoon-Min Kim, Juhee Kim, Jaeog Jeon, Sunmin Park, and Kwang-Hyun Cho**

This file includes:

Supplementary Note 2.
Application of BENEIN to mouse granular cell differentiation, mouse T cell development, and mouse CD8 T cell activation as well as a benchmark for evaluating the effectiveness of BENEIN.

Application of BENEIN to mouse granular cell differentiation - Analysis of single cell transcriptome data of mouse dentate gyrus for BENEIN workflow

To assess the capability of BENEIN in reconstructing Boolean GRN (gene regulatory network) models and in identifying control targets, we applied it to single cell transcriptome data of the mouse dentate gyrus neurogenesis across postnatal development[1] downloaded from http://pklab.med.harvard.edu/velocyto/DentateGyrus/DentateGyrus.loom. This dataset comprises 18,213 cells, with pre-annotated cell types such as neural intermediate progenitor cells. We focused on the differentiation trajectory leading to granular cell types, chosen due to its substantial cell count within a single trajectory (Supplementary Note Figure 1A).

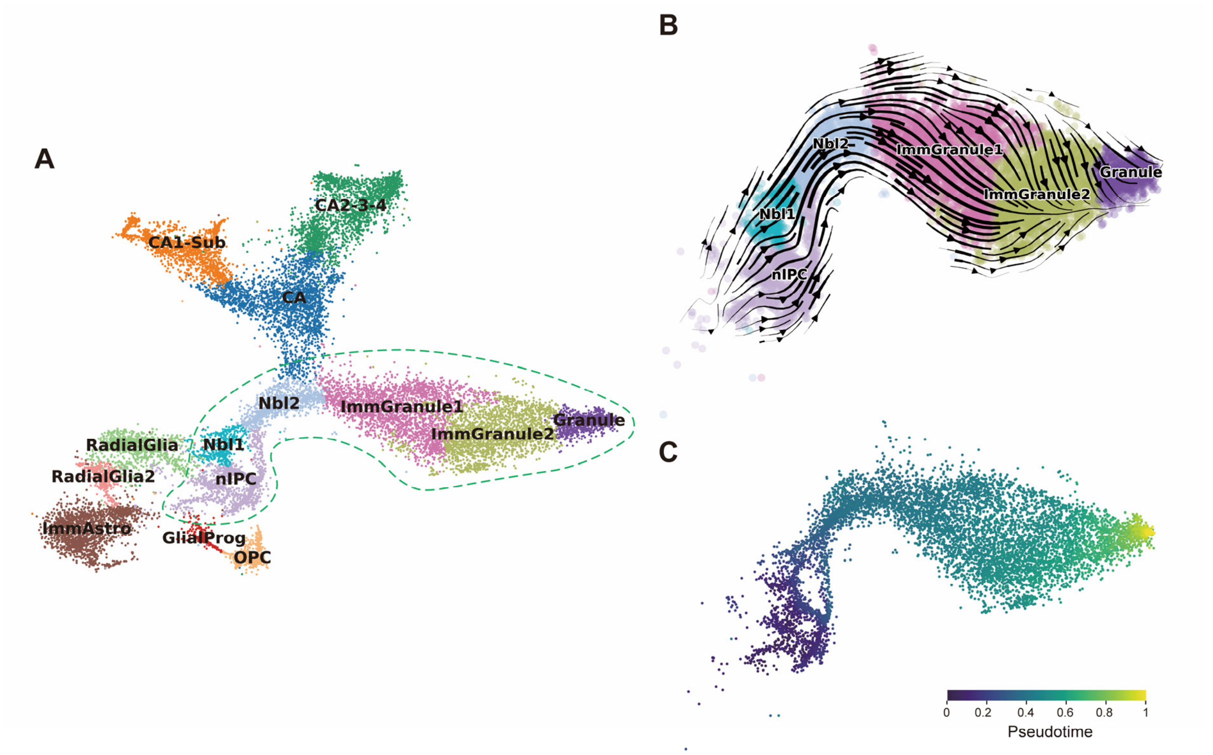

Supplementary Note Figure 1. scVelo analysis of the granular cell differentiation trajectory from mouse dentate gyrus

(A) tSNE (t-distributed stochastic neighbor embedding) visualization with cell types of mouse dentate gyrus single cell transcriptome data. tSNE and cell types were provided with the data. Granular cell differentiation for the study is marked with a green dotted line.

(B) Velocity stream plot of the granular cell differentiation trajectory calculated by scVelo analysis.

(C) Pseudotime (latent time) of the granular cell differentiation trajectory inferred by scVelo analysis.

We selected a subset of neural intermediate progenitor cells (nIPC), neuroblast (Nbl1, Nbl2), immature granule (ImmGranule1, ImmGranule2), and granule cell types composed of 8,074 cells from this data, representing the granular cell differentiation trajectory. We then conducted a scVelo[2] analysis in dynamical mode on this specific trajectory. The purpose was to compute the velocity projection for 2,065 velocity genes and to determine the latent time of the trajectory, based on the dynamical RNA velocity model predicted by scVelo (Supplementary Note Figures 1B and 1C). The latent time is regarded as pseudotime.

Application of BENEIN to mouse granular cell differentiation - Reconstruction of the Boolean GRN model for granular cell differentiation using BENEIN

BENEIN requires the number of windows and the width of the window as parameters for network structure reconstruction. For granular cell differentiation, we used 15 windows with a width of 1000 for the time window of the cells in the trajectory. BENEIN calculated the weights of the TF (transcription factor) – TF and TF – TG (target gene) regulatory backbone networks using conditional mutual information (CMI)[3], based on pseudotime and velocity projection. Subsequently, we employed the ctx step of pySCENIC[4] to refine the backbone network for each window. In this process, we used gene-based motif ranking databases of mm10, motif to transcription factor annotations database, and the list of TFs downloaded from cisTarget resources (https://resources.aertslab.org/cistarget/). The networks from all windows were then merged to form the complete weighted network structure of the granular cell differentiation trajectory, consisting of 1,885 TGs and 94 TFs. For links appearing repeatedly across different windows, we chose the one with the highest weight.

A total of four steps of node and link reductions were performed to reconstruct a Boolean GRN model from the initial structure:

1. It is widely known that the dynamics of GRNs are influenced by feedback [5, 6]. We identified the strongly connected components (SCCs), which include all nodes encompassing feedback in the network. There was one SCC consisting of 1,873 links and 81 nodes, forming a dense network with an average in-degree of 23.12.
2. Given that BENEIN efficiently performs logic inference for nodes with an in-degree of 13 or less, we utilized link weights inferred from the ctx step of pySCENIC to determine the cutoff of link weights. This process yielded a network of 267 links with a maximum in-degree of 13 and 81 nodes.
3. The resulting network from the previous step no longer qualifies as SCC. Therefore, we re-extracted SCCs from the network to identify the core structure for BENEIN, consisting of 140 links and 34

nodes (Supplementary Note Figure 2A).

4. BENEIN performed the binarization of the unspliced/spliced matrix based on the velocity dynamic model fitted for nodes included in this core structure (Supplementary Note Figure 2B). BENEIN integrated these binarized matrices with the network structure by applying the Quine-McCluskey (QM) algorithm-based logic inference to construct the Boolean GRN model. From this process, 47 links were deleted by Boolean function minimization of QM algorithm, and 7 nodes were deleted, including the nodes whose values were fixed to True or False by QM algorithm and the nodes canalized by these nodes.

Through these processes, BENEIN was able to construct the Boolean GRN model of granular cell differentiation, comprising 27 nodes and 93 links (Supplementary Note Figure 2C). The detailed regulatory logics of the Boolean GRN model is provided in Supplementary Note Table 1.

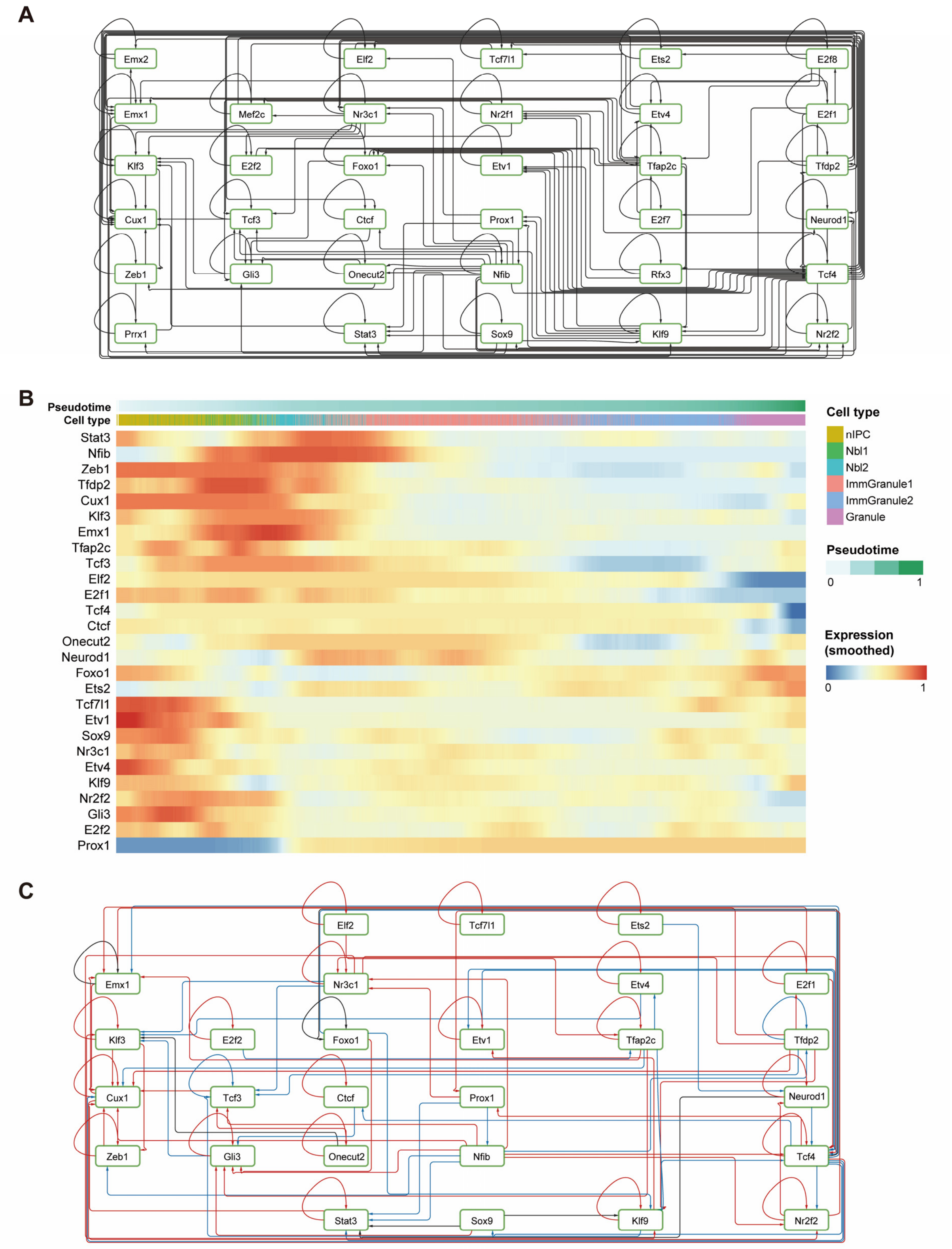

79
80 **Supplementary Note Figure 2. Inference of the Boolean GRN model for granular cell differentiation**

(A) The core structure of granular cell differentiation inferred by BENEIN.

(B) Heatmap illustrating the binarized gene expression across the pseudotime. Binarized gene expressions were smoothed for improved visualization.

(C) The Boolean GRN model of granular cell differentiation reconstructed by BENEIN. The modality of each edge in the network was determined based on the logic's truth table, with edges representing activation colored in red, inhibition in blue, and regulation in black.

Supplementary Note Table 1. Update logic of the Boolean GRN model of mouse granular cell differentiation

<table>
<tr><th>Node</th><th>Update logic</th></tr>
<tr><td>Ctcf</td><td>Ctcf & !Tcf4</td></tr>
<tr><td>Cux1</td><td>(Emx1 & Klf3 & Tcf3) | (Emx1 & Nfib & Tfdp2) | (Cux1 & Tcf3 & !Tcf4) | (Cux1 & Klf3 & Stat3 & Zeb1) | (Klf3 & Nfib & Tfdp2 & Zeb1) | (Cux1 & !Tcf4 & !Tfap2c) | (Cux1 & Emx1 & Tcf3 & !Zeb1) | (Cux1 & Klf3 & Tcf3 & !Tfap2c) | (Emx1 & Stat3 & Zeb1 & !Nfib) | (Emx1 & Nfib & !Tfap2c & !Zeb1) | (Cux1 & Klf3 & Nfib & Tfap2c & !Stat3) | (Cux1 & Klf3 & Stat3 & Tfap2c & !Tcf3) | (Cux1 & Nfib & Tcf3 & Zeb1 & !Tfap2c) | (Cux1 & Tcf3 & Tfdp2 & Zeb1 & !Stat3) | (Cux1 & Stat3 & Zeb1 & !Nfib & !Tcf3) | (Cux1 & Stat3 & Zeb1 & !Nfib & !Tfdp2) | (Cux1 & Stat3 & Tcf3 & Tfap2c & Tfdp2 & !Klf3) | (Cux1 & Nfib & !Tfap2c & !Tfdp2 & !Zeb1) | (Cux1 & Tcf3 & Tfap2c & Zeb1 & !Emx1 & !Nfib) | (Cux1 & !Klf3 & !Stat3 & !Tcf4 & !Zeb1) | (Cux1 & Stat3 & Zeb1 & !Emx1 & !Tcf3 & !Tfap2c) | (Cux1 & Klf3 & !Emx1 & !Stat3 & !Tfap2c & !Zeb1)</td></tr>
<tr><td>E2f1</td><td>E2f1 | Tfdp2</td></tr>
<tr><td>E2f2</td><td>E2f2</td></tr>
<tr><td>Elf2</td><td>Elf2</td></tr>
<tr><td>Emx1</td><td>(Cux1 | Tfdp2) & (E2f1 | Tfdp2) & (Cux1 | !Tcf4) & (Tfap2c | !Tcf4) & (Emx1 | !E2f1 | !Tcf4) & (!Emx1 | !Tcf4 | !Tfdp2)</td></tr>
<tr><td>Ets2</td><td>Ets2</td></tr>
<tr><td>Etv1</td><td>Etv1 | (Klf9 & !Tcf4)</td></tr>
<tr><td>Etv4</td><td>Etv4 | !Tfap2c</td></tr>
<tr><td>Foxo1</td><td>(Foxo1 & Tcf4) | (!Foxo1 & !Tcf4)</td></tr>
<tr><td>Gli3</td><td>(Gli3 | Nfib) & (Gli3 | !Ctcf) & (Ctcf | Nfib | Sox9) & (Ctcf | Nfib | Tfap2c) & (Foxo1 | Gli3 | Sox9) & (Foxo1 | Gli3 | Tfap2c) & (Foxo1 | Tfap2c | !Ctcf | !Nfib) & (Sox9 | Tfap2c | !Ctcf | !Nfib)</td></tr>
<tr><td>Klf3</td><td>(Klf3 | Tcf4) & (Tcf4 | !Etv4) & (Tcf4 | !Onecut2) & (Klf3 | Onecut2 | !Gli3) & (Klf3 | !Etv4 | !Gli3) & (Klf3 | !Etv4 | !Nr3c1) & (Onecut2 | !Etv4 | !Gli3)</td></tr>
<tr><td>Klf9</td><td>(Klf9 & Tfdp2) | (Klf9 & !Tfap2c) | (Foxo1 & Klf9 & Sox9) | (Klf9 & Nr3c1 & !Foxo1) | (Nr3c1 & !Foxo1 & !Tfap2c) | (Nr3c1 & Tfdp2 & !Foxo1 & !Sox9) | (Foxo1 & Nr3c1 & Tfap2c & !Klf9 & !Tfdp2) | (Foxo1 & Sox9 & Tfdp2 & !Nr3c1 & !Tfap2c) | (!Nr3c1 & !Sox9 & !Tfap2c & !Tfdp2)</td></tr>
<tr><td>Neurod1</td><td>Neurod1 & Nr2f2 & !Ets2</td></tr>
<tr><td>Nfib</td><td>!Prox1</td></tr>
</table>

Nr2f2	Nr2f2 & (Cux1 | Nfib | !Tcf4)
Nr3c1	(Nfib | Prox1) & (Prox1 | Tfdp2) & (Nr3c1 | !Prox1) & (!Nfib | !Prox1 | !Tfdp2)
Onecut2	Onecut2
Prox1	Nr2f2 | Tcf4
Sox9	!Tcf4
Stat3	(Sox9 & !Prox1) | (Stat3 & !Tcf4) | (!Neurod1 & !Prox1) | (!Prox1 & !Stat3) | (Neurod1 & Stat3 & !Nfib & !Sox9)
Tcf3	(Onecut2 & !Tfdp2) | (!Klf9 & !Tfdp2) | (!Nr3c1 & !Tfdp2) | (Onecut2 & !Klf9 & !Tcf3) | (Nfib & Onecut2 & !Klf9 & !Nr3c1)
Tcf4	(Nfib & Nr3c1) | (Nr3c1 & Tcf4) | (E2f1 & !Foxo1) | (E2f1 & !Nr3c1) | (Tcf4 & !Etv1) | (!Etv1 & !Foxo1) | (Tcf4 & !Neurod1 & !Nfib) | (!Etv1 & !Klf9 & !Neurod1) | (!Foxo1 & !Klf9 & !Neurod1)
Tcf7l1	Tcf7l1
Tfap2c	Tfap2c & (Elf2 | !E2f2)
Tfdp2	!Nfib | !Tfdp2
Zeb1	Zeb1 & (Klf3 | !Nfib)

Application of BENEIN to mouse granular cell differentiation - Attractor landscape analysis of the Boolean GRN model for granular cell differentiation

We performed simulation analysis and explored the attractor landscape to evaluate the representation of the differentiation trajectory by the Boolean GRN model. Here, the attractor landscape is a set of stable states, known as attractors, which a system eventually reaches, along with their respective basins of attraction. These attractors are associated with cell phenotypes. In the Boolean GRN model, the attractor landscape includes 3,028 point attractors and 797 cyclic attractors. We mapped the point attractors onto the tSNE space using the same method described in the main text. Briefly, we selected the cell(s) with the lowest Hamming distance to its binarized state for each attractor. We then calculated the mean pseudotime of these cells. The cell closest to this mean pseudotime was designated as the representative for the attractor, allowing for the mapping of attractors onto the embedding space. In case of multiple attractors mapped to a single cell, the most similar attractor (the one with the lowest Hamming distance to the cell) was chosen. We found that these point attractors are well-distributed across the tSNE space, signifying that the GRN model is capable of representing the overall features of the data (Supplementary Note Figure 3A).

Furthermore, we quantified the similarity score for each steady state relative to each cell type in the differentiation trajectory. The binarized states for each cell type were determined as outlined in the main text: we calculated an average binarized activity based on the mean binarized state for each cell type, then applied a threshold based on the average across all cells in the differentiation trajectory (Supplementary Note Figure 3B).

Employing these binarized states, we computed the similarity score of each steady state as the Hamming distance between the binarized state of each cell type and the steady states. By projecting these similarity scores onto the tSNE space, we observed a trend of the similarity score to the nIPC/granule state decreasing/increasing over pseudotime (Supplementary Note Figures 3C and 3D).

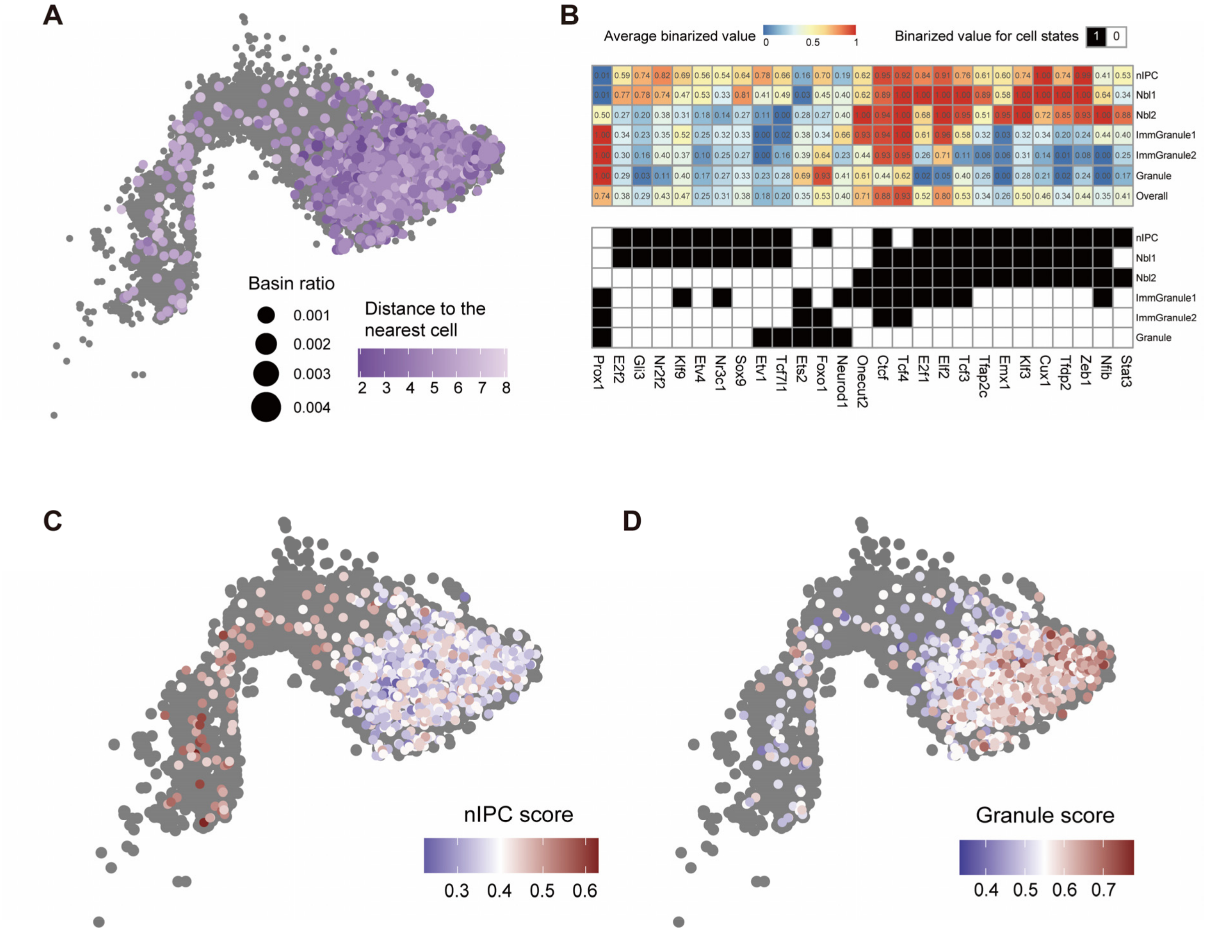

Supplementary Note Figure 3. Analysis of the Boolean GRN model for granular cell differentiation

(A) Point attractors of the Boolean GRN model mapped onto the tSNE space, with the basin ratio and Hamming distance between each attractor and binarized expression of the nearest cell.

(B) Determination of the binarized states for each cell type. Comparing the average binarized expressions of each cell type with the average binarized expressions of all the cells (top) gives the binarized states for each cell type (bottom)

(C, D) Similarity scores of the attractors with respect to nIPC (C) and granule (D) upon the tSNE space.

Application of BENEIN to mouse granular cell differentiation - Identification of optimal control targets for induction of granular cell differentiation

We aimed to identify potential control targets from the Boolean GRN model for inducing granular cell differentiation. After we chose the attractor with the highest pseudotime value as the desired state, BENEIN reduced the reconstructed Boolean GRN model using the BNSimpleReduction algorithm[7].

This reduction aimed to preserve the essential dynamics leading to the desired state. The number of edges in the reduced network decreased significantly, dropping from 93 to 47.

Following this reduction, BENEIN employed the FVS (feedback vertex set) control algorithm on the reduced Boolean GRN to ensure that the controlled network converges to the desired state regardless of initial conditions. This analysis led to the discovery of five FVS configurations, each consisting of 13 elements: ('E2f1', 'E2f2', 'Elf2', 'Ets2', 'Etv1', 'Etv4', 'Foxo1', 'Klf9', 'Nr3c1', 'Onecut2', 'Tcf7l1', 'Tfap2c', 'Zeb1'), ('E2f2', 'Elf2', 'Ets2', 'Etv1', 'Etv4', 'Foxo1', 'Klf9', 'Nfib', 'Nr3c1', 'Onecut2', 'Tcf7l1', 'Tfap2c', 'Zeb1'), ('E2f2', 'Elf2', 'Ets2', 'Etv1', 'Etv4', 'Foxo1', 'Klf9', 'Nr3c1', 'Onecut2', 'Prox1', 'Tcf7l1', 'Tfap2c', 'Zeb1'), ('E2f2', 'Elf2', 'Ets2', 'Etv1', 'Etv4', 'Foxo1', 'Klf9', 'Nr3c1', 'Onecut2', 'Tcf4', 'Tcf7l1', 'Tfap2c', 'Zeb1'), and ('E2f2', 'Elf2', 'Ets2', 'Etv1', 'Etv4', 'Foxo1', 'Klf9', 'Nr3c1', 'Onecut2', 'Tcf7l1', 'Tfap2c', 'Tfdp2', 'Zeb1'). We identified these five minimal FVS configurations as potential control targets (Supplementary Note Figure 4B).

To further reduce the control targets while ensuring sufficient efficacy, we systematically explored all potential combinations in the union of minimal FVSs through attractor simulations. For each combination, we computed average activity vectors and cosine similarities with the desired state. Notably, the simultaneous overexpression of Tcf4 and Klf9 combined with the inhibition of Etv4 exhibited the highest similarity, approximately 0.89. Consequently, Tcf4, Klf9, and Etv4 were identified as the optimal control targets (Supplementary Note Figure 4B).

Previous studies have reported these control targets are related to granular cell differentiation. Haploinsufficiency for Tcf4 was reported to disrupt neurogenesis in the hippocampus of adult mice[8]. Klf9 was reported to be crucial for dentate granule neuron differentiation by behavioral analysis of Klf9-null mice [9]. Depletion of Etv4 was reported to lead differentiation of basal keratinocytes[10].

Overall, the application of BENEIN on mouse granular cell differentiation demonstrates its efficacy in reconstructing Boolean GRN models and identifying control targets for cellular differentiation.

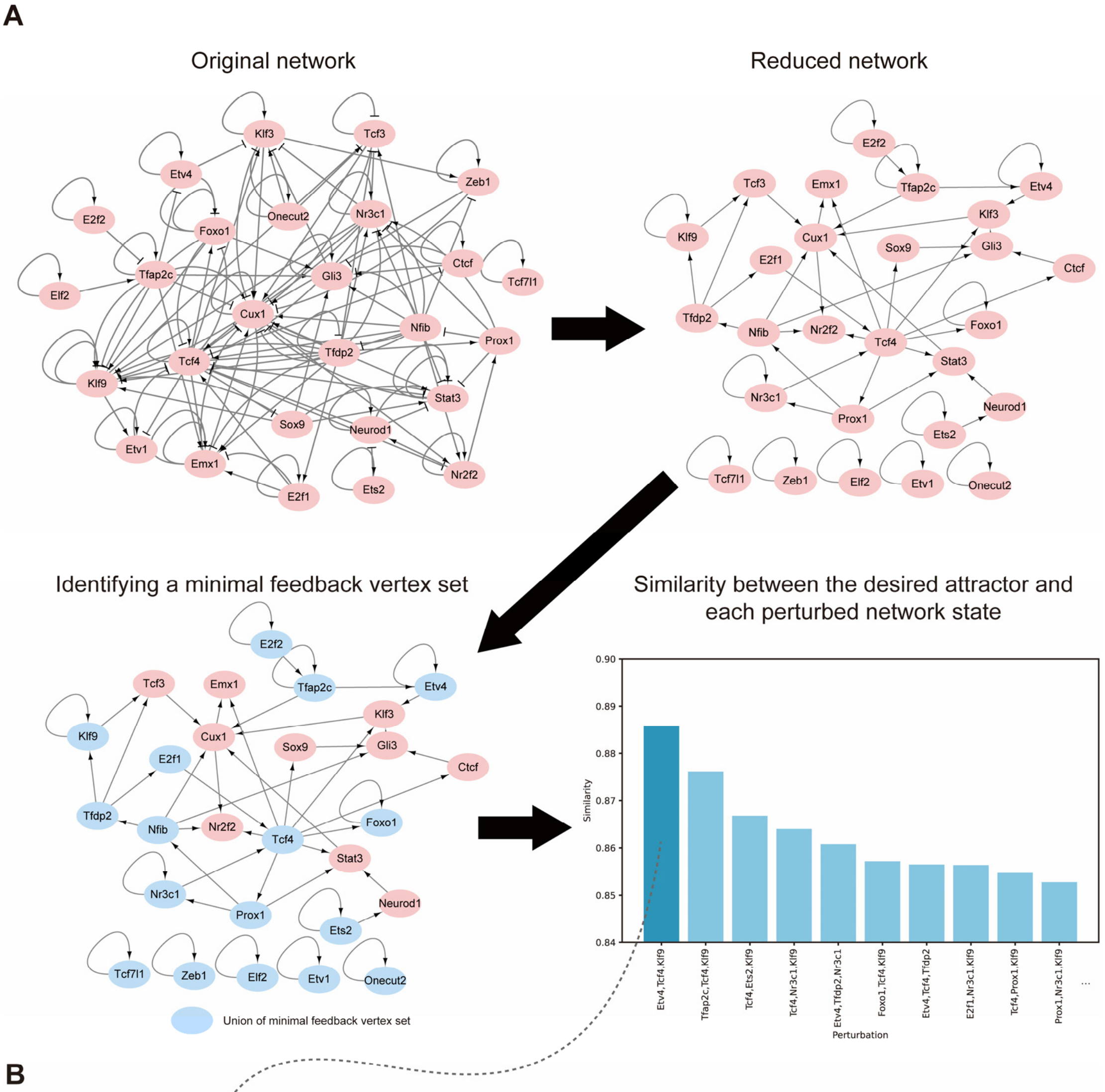

B

Network state	Ctcf	Cux1	E2f1	E2f2	Elf2	Emx1	Ets2	Etv1	Etv4	Foxo1	Gli3	Klf3	Klf9	Neurod1	Nfib	Nr2f2	Nr3c1	Onecut2	Prox1	Sox9	Stat3	Tcf3	Tcf4	Tcf7l1	Tfap2c	Tfdp2	Zeb1
Desired attractor	0	0	1	0	0	0	1	1	0	1	0	1	1	0	0	0	0	1	1	0	0	0	1	1	1	1	0
Tcf4=On, Klf9=On, Etv4=Off	0	0.2	1	0.5	0.5	0	0.5	0.6	0	0.5	0	1	1	0	0	0.1	0.3	0.5	1	0	0	0	1	0.5	0.4	1	0.3

Supplementary Note Figure 4. Identifying optimal control targets for inducing granular cell differentiation

(A) The original network is shown with arrow edges representing activation and bar edges representing inhibition (top left). The reduced network is obtained through the BNSimpleReduction algorithm applied to the original network (top right). The minimal FVSs consisting of 13 nodes are marked in blue in the reduced network (bottom left). The bar plot shows the cosine similarity between the desired attractor and the average activity of each perturbed network. The simultaneous perturbation of Tcf4, Klf9, and Etv4 yields the highest similarity (approximately 0.89), and is chosen as the set of optimal control targets (bottom right).

(B) The average activity vector obtained after the simultaneous perturbation of Tcf4, Klf9, and Etv4 closely resembles the desired attractor vector.

Application of BENEIN to T cell development - Analysis of single-cell RNA-seq data of T cell development for BENEIN workflow

To demonstrate the capability of BENEIN, we applied BENEIN to single-cell RNA-seq data of early mouse T cell development [11] and compared the identified master TFs with the previous studies. The dataset consists of two runs of single-cell RNA-seq, each comprises 1,722 and 4,060 cells after preprocessing, respectively, containing ETP (early T precursor), DN2A (double negative 2A), DN2B (double negative 2B) and DN3 (double negative 3) cells. After excluding a cluster containing majority of ETP cells which showed stable state dynamics of ETP cells and distorting overall RNA velocity dynamics, a trajectory containing 4,582 cells were selected which represents the T cell development. We then conducted a scVelo [2] analysis in dynamical mode on this specific trajectory, with 30 principal components and 30 neighbors for calculating momentum. As a result, velocity estimates of 706 genes and subsequently the latent time for the cells in the trajectory were calculated (Supplementary Note Figure 5A). The latent time is regarded as pseudotime.

Application of BENEIN to T cell development - Reconstruction of the Boolean GRN model for T cell development using BENEIN

BENEIN requires the number of windows and the width of the window as parameters for network structure reconstruction. We used 20 windows with a width of 450 cells for the time window of the cells in the trajectory. BENEIN calculated the weights of the TF–TF and TF–TG backbone networks using conditional mutual information (CMI) [3], based on pseudotime and velocity projection. Subsequently, we employed the ctx step of pySCENIC [4] to refine the backbone network for each window. In this process, we used gene-based motif ranking databases of mm10, motif to transcription factor annotations database, and the list of TFs downloaded from cisTarget resources (https://resources.aertslab.org/cistarget/). The networks from all windows were then merged to form the complete weighted network structure of the T cell development trajectory, consisting of 669 TGs and 24 TFs. For links appearing repeatedly across different windows, we chose the one with the highest weight.

The backbone network from the previous steps were then undergone with node and link reductions to reconstruct a Boolean GRN model from the initial structure. First, we identified the strongly connected components (SCCs), which include all nodes encompassing feedback in the network. This step resulted in one SCC consisting of 240 links and 24 nodes. Secondly, we utilized link weights inferred from the ctx step of pySCENIC to determine the cutoff of link weights to further reduce the network to be

applicable to BENEIN. In detail, we repeatedly removed the edge with the lowest weight until the reduced network has an SCC with a maximum in-degree of 13. After this, we yielded an SCC of 159 links with and 20 nodes ready for the logic inference.

BENEIN performs the binarization of the unspliced/spliced matrix based on the velocity dynamic model fitted for nodes included in this core structure, then integrates these binarized matrices with the network structure by applying the Quine-McCluskey (QM) algorithm-based logic inference to construct the Boolean GRN model. From this process, 51 links were deleted by Boolean function minimization of QM algorithm along with 5 nodes whose values were fixed to True or False after logic inference.

Through these processes, BENEIN was able to construct the Boolean GRN model of T cell development, comprising 15 nodes and 108 links (Supplementary Note Figure 5B). The detailed regulatory logics of the Boolean GRN model is provided in Supplementary Note Table 2.

Supplementary Note Table 2. Update logic of the Boolean GRN model of mouse T cell development

Node	Update logic
Ahr	(Klf3 & ~Ets1 & ~Hmga2) | (Lef1 & ~Mef2c & ~Myb) | (Bhlhe40 & Mef2c & Myb & ~Gata3) | (Lef1 & Myb & ~Gata3 & ~Hmga2) | (Bhlhe40 & ~E2f8 & ~Hmga2 & ~Klf3) | (E2f1 & ~Gata3 & ~Hmga2 & ~Klf3) | (Gata3 & ~Bhlhe40 & ~Ets1 & ~Myb) | (Klf3 & ~Bhlhe40 & ~Hmga2 & ~Mef2c) | (Klf3 & ~E2f1 & ~E2f8 & ~Mef2c) | (Mef2c & ~E2f8 & ~Ets1 & ~Lef1) | (Ahr & Bhlhe40 & E2f1 & ~Hmga2 & ~Myb) | (Ahr & E2f8 & Myb & ~Gata3 & ~Lef1) | (Bhlhe40 & Klf3 & Lef1 & ~Ets1 & ~Gata3) | (E2f1 & Gata3 & Mef2c & ~E2f8 & ~Klf3) | (E2f1 & Lef1 & Myb & ~Bhlhe40 & ~Gata3) | (E2f8 & Gata3 & Lef1 & ~Bhlhe40 & ~Ets1) | (Ets1 & Gata3 & Lef1 & ~E2f8 & ~Klf3) | (Gata3 & Mef2c & Myb & ~Ets1 & ~Klf3) | (Hmga2 & Klf3 & Mef2c & ~E2f1 & ~Ets1) | (Hmga2 & Klf3 & Myb & ~Ahr & ~Gata3) | (Klf3 & Lef1 & Mef2c & ~E2f8 & ~Gata3) | (Ahr & Bhlhe40 & E2f8 & Hmga2 & Myb & ~Klf3) | (Bhlhe40 & E2f8 & Klf3 & Lef1 & Mef2c & ~Hmga2) | (~Bhlhe40 & ~Gata3 & ~Hmga2 & ~Lef1) | (Ahr & Myb & ~Hmga2 & ~Klf3 & ~Lef1) | (Bhlhe40 & E2f8 & ~Ahr & ~Ets1 & ~Myb) | (Bhlhe40 & E2f8 & ~Ets1 & ~Gata3 & ~Klf3) | (Bhlhe40 & Hmga2 & ~E2f1 & ~Gata3 & ~Lef1) | (Bhlhe40 & Mef2c & ~Ahr & ~Klf3 & ~Lef1) | (E2f1 & Myb & ~Ahr & ~Bhlhe40 & ~Gata3) | (Klf3 & Mef2c & ~E2f1 & ~Hmga2 & ~Lef1) | (Ahr & Bhlhe40 & Hmga2 & Mef2c & ~Ets1 & ~Lef1) | (Ahr & Gata3 & Lef1 & Mef2c & ~Bhlhe40 & ~Klf3) | (Ahr & Hmga2 & Klf3 & Mef2c & ~Ets1 & ~Myb) | (Ahr & Lef1 & Mef2c & Myb & ~Bhlhe40 & ~E2f8) | (Ahr & Lef1 & Mef2c & Myb & ~Bhlhe40 & ~Hmga2) | (Bhlhe40 & E2f1 & Gata3 & Mef2c & ~E2f8 & ~Myb) | (Bhlhe40 & E2f8 & Ets1 & Lef1 & ~Ahr & ~E2f1) | (E2f1 & Ets1 & Lef1 & Myb & ~Gata3 & ~Klf3) | (Ahr & Bhlhe40 & E2f1 & E2f8 & Hmga2 & Myb & ~Ets1) | (E2f1 & Ets1 & Gata3 & Hmga2 & Klf3 & Lef1 & ~Mef2c) | (Ets1 & ~Ahr & ~E2f8 & ~Gata3 & ~Myb) | (Ahr & Bhlhe40 & Ets1 & ~Gata3 & ~Klf3 & ~Lef1) | (Ahr & Bhlhe40 & Lef1 & ~E2f8 & ~Ets1 & ~Gata3) | (Ahr & E2f8 & Ets1 & ~Bhlhe40 & ~Gata3 & ~Mef2c) | (Ahr & Gata3 & Hmga2 & ~Bhlhe40 & ~E2f8 & ~Klf3) | (Ahr & Gata3 & Myb & ~Ets1 & ~Klf3 & ~Lef1) | (Gata3 & Mef2c & Myb & ~Ahr & ~Bhlhe40 & ~Klf3) | (Hmga2 & Klf3 & Myb & ~Gata3 & ~Lef1 & ~Mef2c) | (E2f8 & Gata3 & Hmga2 & Klf3 & Mef2c & ~Bhlhe40 & ~Lef1) | (~Ets1 & ~Gata3 & ~Klf3 & ~Mef2c & ~Myb) | (Ahr & E2f8 & ~E2f1 & ~Ets1 & ~Gata3 & ~Myb) | (E2f1 & Mef2c & ~Bhlhe40 & ~E2f8 & ~Gata3 & ~Myb)
Bhlhe40	Bhlhe40 | ~Gata3
E2f1	E2f1 | (Lef1 & ~Myb)
E2f2	E2f2 | (Gata3 & Myb)

E2f8	(Mef2c & Zbtb16 & ~Lef1 & ~Myb) | (E2f8 & ~E2f1 & ~Ets1 & ~Lef1) | (E2f8 & ~Ets1 & ~Lef1 & ~Zbtb16) | (E2f8 & Ets1 & Gata3 & ~Hmga2 & ~Myb) | (E2f1 & E2f8 & Ets1 & Gata3 & Myb & ~Mef2c) | (E2f8 & Gata3 & Lef1 & Myb & Zbtb16 & ~Hmga2) | (E2f1 & Gata3 & ~Lef1 & ~Mef2c & ~Zbtb16) | (E2f8 & Myb & ~Ets1 & ~Hmga2 & ~Mef2c) | (E2f8 & Runx3 & ~Ets1 & ~Gata3 & ~Zbtb16) | (E2f8 & Zbtb16 & ~E2f1 & ~Ets1 & ~Hmga2) | (E2f8 & Gata3 & Myb & Zbtb16 & ~Lef1 & ~Mef2c) | (E2f1 & E2f8 & Ets1 & Mef2c & Myb & Zbtb16 & ~Hmga2) | (E2f1 & E2f8 & Hmga2 & Lef1 & Mef2c & Myb & Runx3 & Zbtb16) | (E2f8 & Gata3 & Lef1 & ~Hmga2 & ~Mef2c & ~Myb) | (E2f8 & Myb & Zbtb16 & ~E2f1 & ~Hmga2 & ~Mef2c) | (E2f1 & E2f8 & Gata3 & Mef2c & Zbtb16 & ~Hmga2 & ~Lef1) | (E2f1 & E2f8 & Lef1 & Myb & Zbtb16 & ~Gata3 & ~Hmga2) | (E2f8 & Ets1 & Hmga2 & Lef1 & Mef2c & Myb & Runx3 & ~Gata3) | (E2f1 & Gata3 & ~E2f8 & ~Ets1 & ~Hmga2 & ~Mef2c)
Ets1	(Ets1 | ~E2f8) & (Gata3 | ~E2f1) & (Hmga2 | ~Myb) & (E2f8 | Gata3 | Hmga2) & (~E2f1 | ~Myb) & (E2f8 | ~Ets1 | ~Gata3) & (Gata3 | ~E2f8 | ~Myb) & (Hmga2 | ~E2f1 | ~Ets1)
Gata3	(Gata3 & ~E2f8 & ~Runx3) | (E2f8 & Ets1 & Gata3 & Hoxa9 & Myb) | (Ets1 & Gata3 & Hoxa9 & Klf3 & Myb) | (Gata3 & Klf3 & ~E2f1 & ~Hmga2) | (Gata3 & Klf3 & ~E2f8 & ~Hmga2) | (Gata3 & Klf3 & ~Hmga2 & ~Lef1) | (Gata3 & Klf3 & ~Hmga2 & ~Myb) | (E2f8 & Ets1 & Gata3 & Lef1 & ~Hmga2) | (E2f8 & Gata3 & Klf3 & Myb & ~Hoxa9) | (Ets1 & Gata3 & Hoxa9 & Lef1 & ~Hmga2) | (Ets1 & Klf3 & Myb & ~E2f1 & ~E2f8) | (Gata3 & Hoxa9 & Klf3 & ~E2f8 & ~Hmga2) | (Gata3 & Klf3 & Myb & ~E2f8 & ~Hoxa9 & ~Lef1)
Hmga2	Hoxa9 | (Hoxa9 & Myb) | (E2f8 & Gata3 & Lef1) | (E2f8 & Myb & Runx3) | (~Ahr & ~Gata3) | (~Ets1 & ~Lef1) | (Ahr & Runx3 & ~E2f1) | (E2f1 & Ets1 & ~Lef1) | (E2f8 & Hmga2 & ~Myb) | (Gata3 & Myb & ~Hmga2) | (Hmga2 & Myb & ~Gata3) | (Myb & ~Ets1 & ~Hmga2) | (~Ets1 & ~Myb & ~Runx3) | (E2f1 & Myb & ~Ahr & ~Ets1)
Hoxa9	Hoxa9 & (E2f1 | Gata3)
Klf3	(E2f8 & Ets1 & Hmga2 & Klf3 & Lef1 & ~Myb) | (E2f8 & Klf3 & ~Hmga2 & ~Lef1 & ~Myb) | (Gata3 & Klf3 & ~Ets1 & ~Hmga2 & ~Myb) | (Gata3 & Zbtb16 & ~Ahr & ~E2f1 & ~Hmga2) | (E2f1 & Klf3 & Lef1 & ~Ahr & ~Ets1 & ~Myb) | (Ets1 & Klf3 & Lef1 & ~Ahr & ~E2f1 & ~Myb) | (~E2f1 & ~Ets1 & ~Hmga2 & ~Lef1 & ~Zbtb16) | (Hmga2 & ~Ahr & ~E2f1 & ~Ets1 & ~Lef1 & ~Myb) | (Ahr & Klf3 & Runx3 & ~Ets1 & ~Gata3 & ~Lef1 & ~Myb) | (Ets1 & Klf3 & Runx3 & ~Ahr & ~E2f8 & ~Hmga2 & ~Lef1)
Lef1	(Hmga2 & ~Bhlhe40) | (Ets1 & Lef1 & Runx3) | (~Gata3 & ~Myb) | (Bhlhe40 & Lef1 & ~Ets1) | (E2f1 & Hmga2 & ~E2f8) | (E2f1 & Runx3 & ~Gata3) | (Gata3 & ~Bhlhe40 & ~E2f1) | (Bhlhe40 & E2f1 & Hmga2 & ~Myb) | (E2f1 & Lef1 & Myb & ~Bhlhe40) | (E2f8 & Ets1 & Gata3 & ~Bhlhe40) | (~Bhlhe40 & ~E2f8 & ~Ets1) | (~E2f1 & ~E2f8 & ~Ets1) | (~Ets1 & ~Lef1 & ~Myb) | (Bhlhe40 & E2f8 & Ets1 & Hmga2 & ~E2f1)
Mef2c	(E2f8 & Ets1) | (Hmga2 & Runx3) | (Ahr & ~Ets1) | (Hoxa9 & ~Myb) | (Gata3 & Myb & Runx3) | (~Ahr & ~Hoxa9) | (~E2f8 & ~Ets1) | (Gata3 & Mef2c & ~Myb) | (Ahr & Hoxa9 & Mef2c & Runx3) | (~Gata3 & ~Mef2c & ~Myb)
Myb	Bhlhe40 & Ets1 & Gata3 & Hmga2 & ~E2f1 & ~Lef1
Runx3	(~Gata3 & ~Hmga2) | (E2f1 & Ets1 & Myb & ~Gata3) | (~E2f2 & ~Gata3 & ~Myb) | (E2f1 & Hmga2 & ~E2f2 & ~Ets1) | (Ets1 & Runx3 & ~Gata3 & ~Lef1) | (Gata3 & Myb & ~E2f1 & ~Ets1) | (E2f1 & Gata3 & Hmga2 & Lef1 & ~Ets1) | (Ets1 & Gata3 & Hmga2 & Lef1 & ~E2f1) | (E2f1 & ~E2f2 & ~Ets1 & ~Myb) | (E2f1 & ~Ets1 & ~Gata3 & ~Lef1) | (Gata3 & ~E2f2 & ~Ets1 & ~Lef1) | (E2f1 & Hmga2 & Runx3 & ~E2f2 & ~Lef1) | (E2f1 & Hmga2 & Runx3 & ~Lef1 & ~Myb) | (E2f2 & Hmga2 & Runx3 & ~Ets1 & ~Myb)
Zbtb16	(Myb & Zbtb16) | (Runx3 & Zbtb16) | (Zbtb16 & ~E2f2) | (~E2f2 & ~Ets1) | (Ahr & Zbtb16 & ~Lef1) | (Runx3 & ~Ahr & ~Ets1)

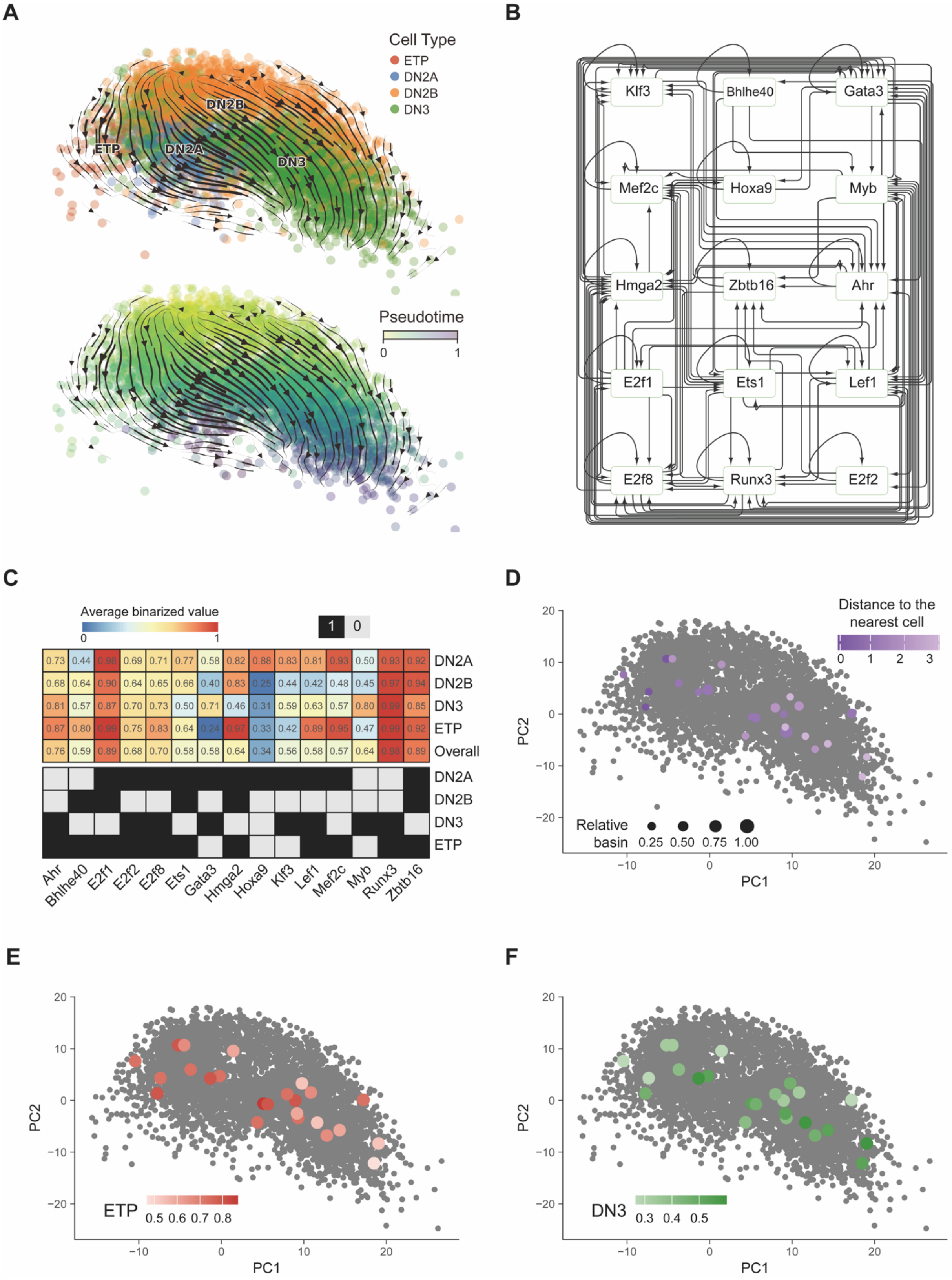

Supplementary Note Figure 5. Inference and analysis of the Boolean GRN model for T cell development

(A) Single-cell RNA-seq data of CD8 T cell development upon the PC embedding space with velocity stream plot (top) and pseudotime (bottom).

(B) The Boolean GRN model for T cell development constructed by BENEIN.

(C) Heatmap illustrating the average gene expression and the binarized states for each cell type.

(D) 25 attractors of the Boolean GRN model mapped onto the PC embedding space, with the relative basin of attraction and Hamming distance between each attractor and the binarized expression of the nearest cell.

(E-F) Similarity scores of the attractors with respect to the ETP state (E) and the DN3 state (F) upon the PC embedding space.

Application of BENEIN to T cell development - Attractor landscape analysis of the Boolean GRN model for T cell development

We performed simulation analysis and explored the attractor landscape to evaluate the representation of the differentiation trajectory by the Boolean GRN model. Here, the attractor landscape is a set of stable states, known as attractors, which a system eventually reaches, along with their respective basins of attraction. These attractors are associated with cell phenotypes. In the Boolean GRN model, the attractor landscape includes 21 point attractors and 2 cyclic attractors with period of 2. We mapped the attractors onto the PC (principal component) space and found that these attractors are well-distributed across the embedding space, signifying that the GRN model can represent the overall features of the data (Supplementary Note Figure 5D).

Furthermore, we quantified the similarity score for each steady state relative to ETP and DN3 state in the differentiation trajectory. The binarized states for each cell type were determined as outlined in the main text: we calculated an average binarized activity based on the mean binarized state for each cell type, then applied a threshold based on the average across all cells in the differentiation trajectory.

Employing these binarized states (Supplementary Note Figure 5D), we computed the similarity score of each steady state as the Hamming distance between the binarized state of each cell type and the steady states. By projecting these similarity scores onto the embedding space, we observed a trend of the similarity score to the ETP / DN3 state decreasing/increasing over pseudotime (Supplementary Note Figure 5E-F).

Application of BENEIN to T cell development - Identification of optimal control targets for induction of T cell development

After we chose the attractor with the highest pseudotime value as the desired state, BENEIN reduced the reconstructed Boolean GRN model using the BNSimpleReduction algorithm[7]. This reduction aimed to preserve the essential dynamics leading to the desired state. Following this reduction, BENEIN

employed the FVS (feedback vertex set) control algorithm on the reduced Boolean GRN to ensure that the controlled network converges to the desired state regardless of initial conditions. This analysis led to discover the union of FVS consists of 10 nodes which served as potential control target candidates. We aimed to find a single target that can promote the T cell development most efficiently in the model, enabling comparison with the other GRN inference-based tools such as SCENIC and VIPER. For the potential control target candidates, we computed average activity vectors and cosine similarities with the desired state(Supplementary Note Figure 6A). As a result, Gata3 overexpression followed by Kfl3 overexpression and E2f1 knockdown were chosen as top-ranking control target candidates. We found that the attractor landscape of the perturbed GRN model with Gata3 overexpression showed that the attractors mapped to the cells with lower pseudotime are either decreased or eliminated, while the basin of the attractors mapped to the cells with higher pseudotime increased (Supplementary Note Figure 6B).

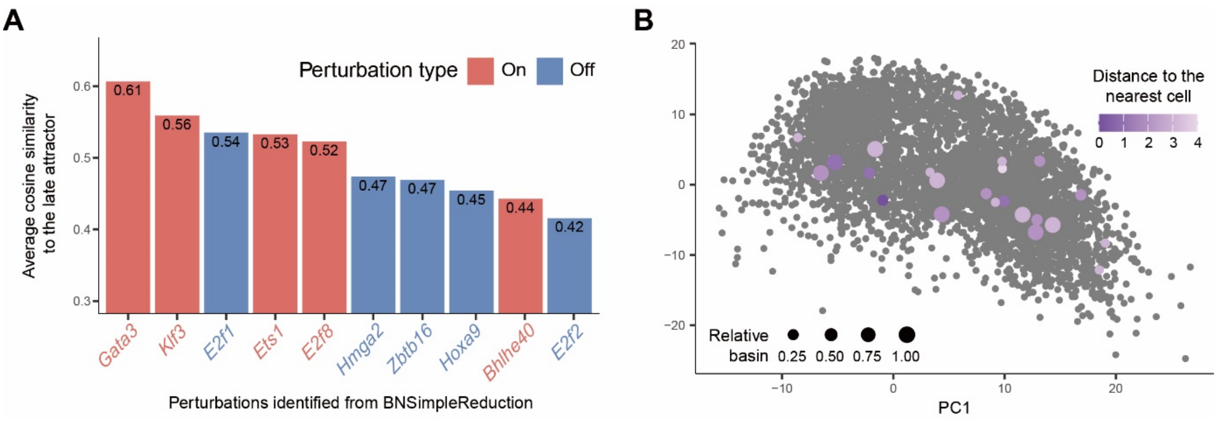

Supplementary Note Figure 6. Identification of an optimal control target for T cell development

(A) Bar plot of average cosine similarity between the desired attractor and the average activity of each perturbed network. The control target candidates and perturbation types were identified by BNSimpleReduction.

(B) 24 attractors of the perturbed Boolean GRN model with Gata3 overexpression mapped onto the PC embedding space, with the relative basin of attraction and Hamming distance between each attractor and the binarized expression of the nearest cell.

Application of BENEIN to T cell development - Benchmarking BENEIN with SCENIC and VIPER in identification of the master regulators for T cell development

To benchmark BENEIN with the other algorithms, we performed SCENIC and VIPER with the same data. Specifically, the algorithms were run with the same T cell development trajectory data, and top-ranked regulators that are highly enriched in the DN3 clusters were chosen. While the other algorithms struggled to find potential master regulators promoting T cell development, BENEIN was able to identify more plausible potential master regulators supported by previous studies (Supplementary Note

Table 3). Of the top three Gata3, Klf3 and E2f1 TFs identified by BENEIN, Gata3 is the first to be reported as a master regulator [12] and Klf3 is the second to be reported as a potential master regulator during the development of DN3 T cells in mouse thymus [13]. In contrast, only Egr1 of the top three master regulators identified by SCENIC, Mafb, Egr1 and Smad3, is reported to be a master regulator of DN3 T cell development [14]. Moreover, none of the master regulators identified by VIPER, Ybx1, Mis18bp1 and Nme2, are known to be master regulators.

Overall, the application of BENEIN on mouse T cell development demonstrates its efficacy in reconstructing Boolean GRN models as well as better performance in identifying master regulator candidates than the other GRN inference-based algorithms.

Top3 by BENEIN	Ref.	Top3 by SCENIC	Ref.	Top3 by VIPER	Ref.
Gata3	O	Mafb	X	Ybx1	X
Klf3	O	Egr1	O	Mis18bp1	X
E2f1	X	Smad3	X	Nme2	X

Supplementary Note Table 3. Comparison of top-ranked genes identified by BENEIN, SCENIC and VIPER for T cell development.

'O' denotes genes that are significantly up-regulated during T cell development or are known master regulators of DN3 T cell [12-15], while 'X' denotes genes that are not yet known or are not master regulators of DN3 T cell.

Application of BENEIN to CD8 T cell activation - Analysis of single-cell RNA-seq data of CD8 T cell activation for BENEIN workflow

To further demonstrate the capability of BENEIN, we applied BENEIN to single-cell RNA-seq data of mouse T cell activation [16] and compared the identified master TFs with the previous studies. The data comprises 4,865 cells after preprocessing, containing naïve, early effector, and effector cell types of T cell activation. We then conducted a scVelo[2] analysis in dynamical mode on this trajectory, with 50 principal components and 500 neighbors for calculating momentum. As a result, velocity estimates of 193 genes and subsequently the latent time for the cells in the trajectory were calculated (Supplementary Note Figure 7A). The latent time is regarded as pseudotime. BENEIN performed the binarization of the unspliced/spliced matrix based on the velocity dynamic model fitted for these genes with velocity estimates.

Application of BENEIN to CD8 T cell activation - Reconstruction of the Boolean GRN model for CD8 T cell activation using BENEIN

For network structure reconstruction using BENEIN, we used 40 windows with a width of 250 cells for the time window of the cells in the trajectory. After calculating conditional mutual information (CMI)[3] and subsequently refining the backbone network with pySCENIC as in the T cell development case, we were able to obtain network structure of the CD8 T cell activation trajectory, consisting of 189 TGs and 19 TFs. For links appearing repeatedly across different windows, we chose the one with the highest weight.

The backbone network from the previous steps were then undergone with node and link reductions to reconstruct a Boolean GRN model from the initial structure. First, we identified the strongly connected components (SCCs), which include all nodes encompassing feedback in the network. This step resulted in one SCC consisting of 288 links and 19 nodes.

Secondly, we excluded the nodes that does not show variability in the binarized matrices. Nodes with less than 5% of variations were excluded, resulting in a network with 5 nodes and 23 links ready for the logic inference.

BENEIN integrates the binarized matrices with the network structure by applying the Quine-McCluskey (QM) algorithm-based logic inference to construct the Boolean GRN model. From this process, 6 links were deleted by Boolean function minimization of QM algorithm. Through these processes, BENEIN was able to construct the Boolean GRN model of T cell activation, comprising 5 nodes and 17 links (Supplementary Note Figure 7B). The detailed regulatory logics of the Boolean GRN model is provided in Supplementary Note Table 4.

Supplementary Note Table 4. Update logic of the Boolean GRN model of mouse T cell activation

Node	Update logic
Eomes	(Eomes & ~Stat1) | (Eomes & ~Tbx21)
Fosl2	Stat1 | ~Eomes | (Fosl2 & Tbx21)
Rbpj	Eomes
Stat1	(Stat1 & ~Eomes) | (~Eomes & ~Fosl2) | (~Eomes & ~Rbpj) | (Rbpj & ~Fosl2 & ~Tbx21)
Tbx21	Stat1 | ~Eomes | (Fosl2 & Tbx21)

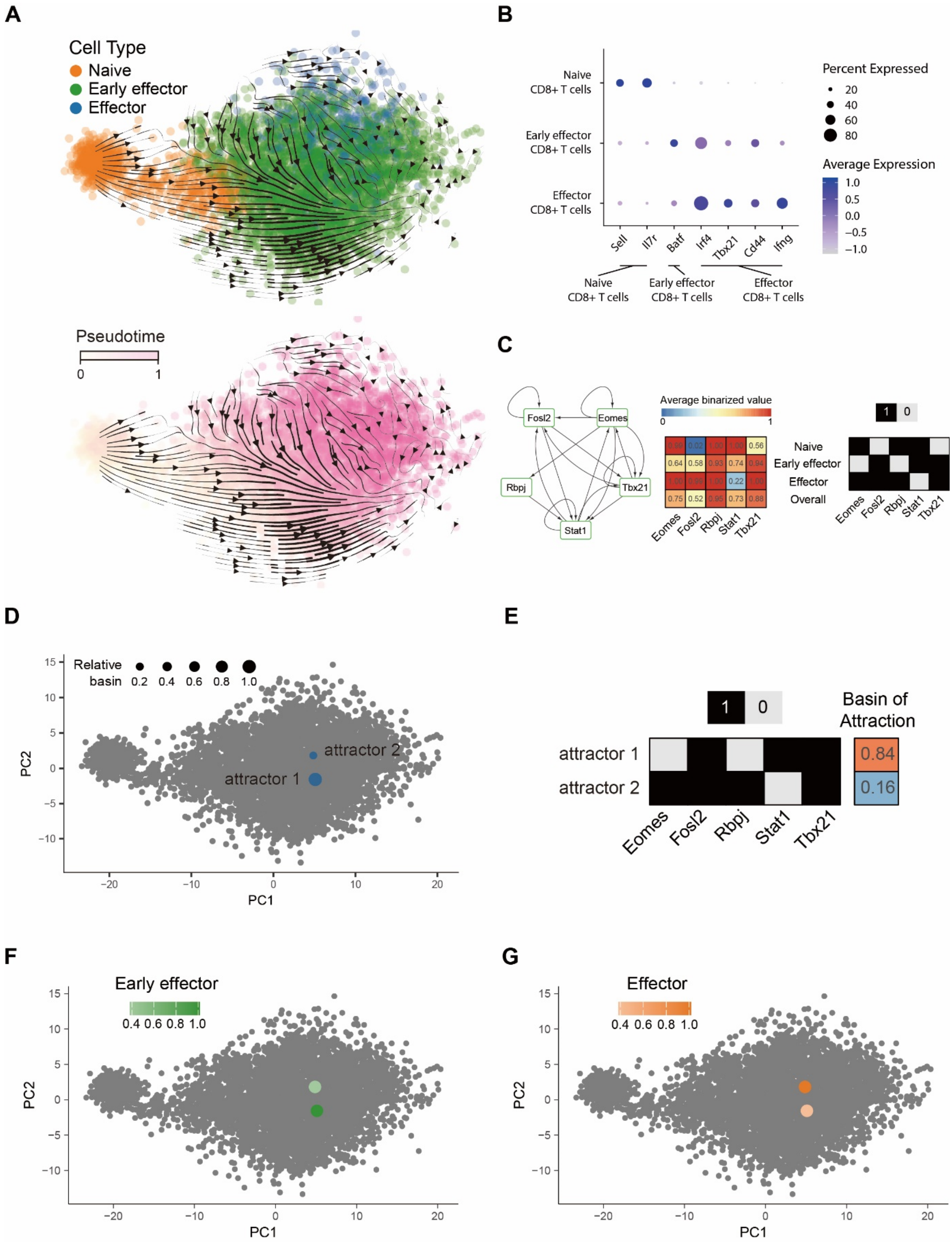

Supplementary Note Figure 7. Inference and analysis of the Boolean GRN model for CD8 T cell activation.

(A) Single-cell RNA-seq data of T cell activation upon the PC embedding space with velocity stream plot (top)

and pseudotime (bottom).

(B) Dot plot shows expression levels of marker genes for naïve CD8 T cells, early effector CD8 T cells, and effector CD8 T cells.

(C) The Boolean GRN model for T cell activation constructed by BENEIN (left). Heatmap illustrating the average gene expression (middle) and the binarized states for each cell type (right).

(D) Two attractors of the Boolean GRN model mapped onto the PC embedding space, with the relative basin of attraction.

(E) Heatmap of the two attractor states and their basin of attraction.

(F-G) Similarity scores of the attractors with respect to the early effector state (F) and the effector state (G) upon the PC embedding space.

Application of BENEIN to CD8 T cell activation - Attractor landscape analysis of the Boolean GRN model for T cell activation

We performed simulation analysis and explored the attractor landscape to evaluate the representation of the differentiation trajectory by the Boolean GRN model. In the Boolean GRN model, the attractor landscape includes two point attractors (Supplementary Note Figure 7D-E). Then we quantified the similarity score for each steady state relative to the cell types in the differentiation trajectory. Employing the binarized states for the cell types (Supplementary Note Figure 7C), we computed the similarity score of each steady state as the Hamming distance between the binarized state of each cell type and the steady states. By mapping the attractors onto the PC (principal component) space along with the similarity scores, we found that the two attractors each represent early effector cells and effector cells, respectively (Supplementary Note Figure 7F-G).

Application of BENEIN to CD8 T cell activation - Identification of optimal control targets for induction of T cell activation

After we chose the attractor that represents effector cell type, BENEIN reduced the reconstructed Boolean GRN model using the BNSimpleReduction algorithm[7] followed by FVS (feedback vertex set) identification on the reduced Boolean GRN to ensure that the controlled network converges to the desired state regardless of initial conditions. This analysis led to the discovery of FVS consists of 3 nodes (Eomes ON, Fosl2 ON, Tbx21 ON) which served as potential control target candidates.
We aimed to find a single target that can promote the T cell activation most efficiently in the model, enabling comparison with the other GRN inference-based tools such as SCENIC and VIPER. For the potential control target candidates found previously, we computed average activity vectors and cosine similarities with the desired state. As a result, Eomes, followed by Fosl2 and Tbx21, all of them with

overexpression, were chosen as top-ranking control target candidates (Supplementary Note Figure 8A). We found that the attractor landscape of the perturbed GRN model with Eomes overexpression showed that every initial states of the network model converged to the attractor representing effector cell type, ensuring the most effective target on the network model (Supplementary Note Figure 8B).

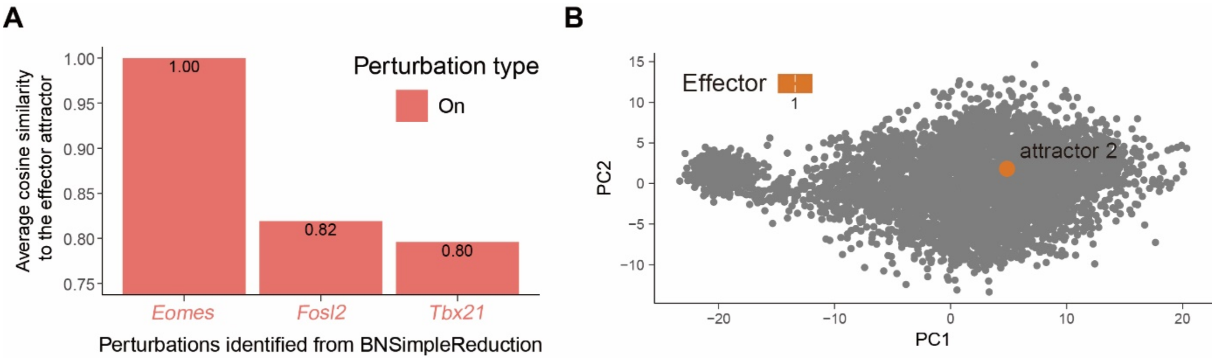

Supplementary Note Figure 8. Identification of an optimal control target for CD8 T cell activation

(A) Bar plot of average cosine similarity between the desired attractor and the average activity of each perturbed network. The control target candidates and perturbation types were identified by BNSimpleReduction.

(B) One point attractor of the perturbed Boolean GRN model with Eomes overexpression mapped onto the PC embedding space, with the similarity score to the effector state.

Application of BENEIN to CD8 T cell activation - Benchmarking BENEIN with SCENIC and VIPER in identification of the master regulators for T cell activation

To benchmark BENEIN with the other algorithms, we performed SCENIC and VIPER with the same data. Specifically, the algorithms were run with the same data of T cell activation trajectory, and top-ranked regulators that are highly enriched in the effector cell clusters were chosen. While the other algorithms struggled to find potential master regulators, BENEIN was able to identify more plausible potential master regulators supported by previous studies (Supplementary Note Table 5). Of the top three Eomes, Fosl2, and Tbx21 TFs identified by BENEIN, the all of top three TFs as master regulators during the CD8 T cells activation. On the other hand, among the Mafb, Egr1, and Smad3 identified by SCENIC, Nfatc1 and Egr3 are known to be master regulators of CD8 T cell activation, and among the Aff3, Irf1, and Egr3 identified by VIPER, Irf1, and Egr3 are known to be master regulators of CD8 T cell activation. Overall, the application of BENEIN on mouse CD8 T cell activation demonstrates its efficacy in reconstructing Boolean GRN models as well as better performance in identifying master regulator candidates than the other GRN inference-based algorithm.

Top3 by BENEIN	Ref.	Top3 by SCENIC	Ref.	Top3 by VIPER	Ref.
Eomes	○	Egr1	×	Aff3	×
Fosl2	○	Nfatc1	○	Irf1	○
Tbx21	○	Egr3	○	Egr3	○

Supplementary Note Table 5. Comparison of top-ranked genes identified by BENEIN, SCENIC and VIPER for CD8 T cell activation.

'O' denotes genes that are significantly up-regulated during T cell activation or are known master regulators of CD8 T cell activation [17-21], while 'X' denotes genes that are not yet known or are not master regulators of CD8 T cell activation.

Benchmarking BENEIN with SCENIC and VIPER in master regulator identification for human colon enterocyte differentiation

To benchmark the master regulators identified with BENEIN against the other algorithms, we applied VIPER, SCENIC, and DEG analysis to the human colon enterocyte differentiation single-cell RNA-seq data used in the manuscript. To evaluate the potential impact of the candidates identified by each method, we performed Gene Set Variation Analysis (GSVA) [22] with differentiation-related gene set signatures, employing expression data of HT-29 colon cell line within the L1000 dataset [23].

From the results, we found that while the candidate master regulators identified by VIPER, SCENIC and DEG analysis implicate some differentiation effects on HT-29 cells compared to DMSO, the three master regulators (MYB, HDAC2, and FOXA2) identified by BENEIN are implicated to be more effective. Furthermore, predictions based on the L1000 dataset suggest that the regulators identified by BENEIN may result in similar or better differentiation effects compared to those identified by the other methods (Supplementary Note Figure 9).

In summary, BENEIN outperforms VIPER, SCENIC, and DEG analysis by providing dynamic insights into regulatory mechanisms and predicting synergistic interactions between TFs that are crucial for effective control of cellular states (Supplementary Note Table 6). These findings further demonstrate proficiency of BENEIN in reconstructing Boolean GRN models and identifying master regulators from single-cell RNA-seq data.

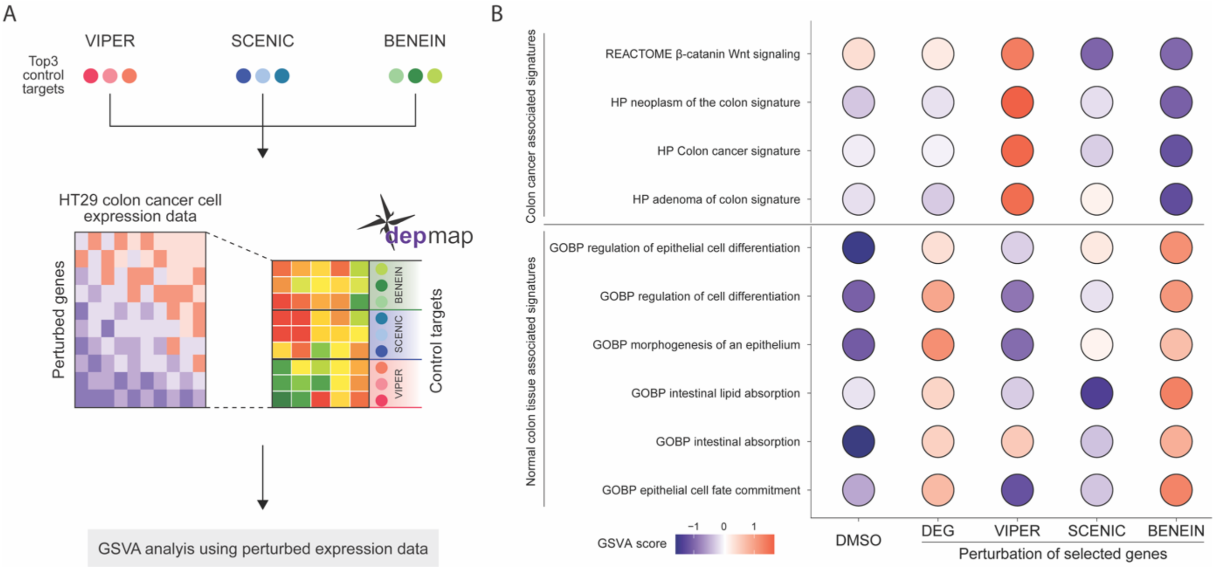

Supplementary Note Figure 9. Comparing the performance of the BENEIN to DEG, VIPER, and SCENIC in identifying key TFs in cell fate decisions.

(A) The schematic figure illustrates the procedures for comparative performance assessment of the top three key TFs identified by BENEIN (FOXA2, CREB3L1, and MYB), VIPER (ASCL2, SOX4, and PROX1), SCENIC (MYC, ETS2, and HMGA1), and DEG (ASCL2, SMOC2, and LGR5). The analysis employed HT29 colon cancer cell Dependency Map (DepMap) data to investigate the potential impact of regulating the top three key TFs identified by each algorithm on colon cancer tumorigenesis and reversion of colon cancer by GSVA [22]

(B) The dot plot illustrates that BENEIN outperforms the other algorithms. In particular, the loss of FOXA2, CREB3L1, and MYB, identified by BENEIN, may suppress colon cancer-associated signatures while inducing normal colon tissue-associated signatures.

	Differentially expressed genes	VIPER	SCENIC	BENEIN
Identifying master regulator of specific type of cells	✕	○	○	○
Identifying combination targets to drive cells to the desired state.	✕	○	✕	○
In silico perturbation simulation	✕	✕	✕	○
Simulation of cell state transitions based on cellular molecular dynamics.	✕	✕	✕	○
Discovery of the underlying molecular mechanism through analysis of mathematical model	✕	✕	✕	○

Supplementary Note Table 6. A comparison of BENEIN with other methods.

The performance of differentially expressed genes (DEGs), VIPER, and SCENIC was evaluated in comparison to BENEIN with the objective of assessing their capacity to identify master TFs.

Reference

1. Hochgerner, H., et al., *Conserved properties of dentate gyrus neurogenesis across postnatal development revealed by single-cell RNA sequencing.* Nat Neurosci, 2018. **21**(2): p. 290-299.
2. Bergen, V., et al., *Generalizing RNA velocity to transient cell states through dynamical modeling.* Nat Biotechnol, 2020. **38**(12): p. 1408-1414.
3. Qiu, X., et al., *Inferring Causal Gene Regulatory Networks from Coupled Single-Cell Expression Dynamics Using Scribe.* Cell Syst, 2020. **10**(3): p. 265-274 e11.
4. Van de Sande, B., et al., *A scalable SCENIC workflow for single-cell gene regulatory network analysis.* Nat Protoc, 2020. **15**(7): p. 2247-2276.
5. Kim, J.R., Y. Yoon, and K.H. Cho, *Coupled feedback loops form dynamic motifs of cellular networks.* Biophys J, 2008. **94**(2): p. 359-65.
6. Thomas, R., D. Thieffry, and M. Kaufman, *Dynamical behaviour of biological regulatory networks--I. Biological role of feedback loops and practical use of the concept of the loop-characteristic state.* Bull Math Biol, 1995. **57**(2): p. 247-76.
7. Yang, J.M., C.K. Lee, and K.H. Cho, *Stabilizing Control of Complex Biological Networks Based on Attractor-Specific Network Reduction.* Ieee Transactions on Control of Network Systems, 2021. **8**(2): p. 928-939.
8. Braun, K., et al., *Enriched environment ameliorates adult hippocampal neurogenesis deficits in Tcf4 haploinsufficient mice.* BMC Neurosci, 2020. **21**(1): p. 50.
9. Scobie, K.N., et al., *Kruppel-like factor 9 is necessary for late-phase neuronal maturation in the developing dentate gyrus and during adult hippocampal neurogenesis.* J Neurosci, 2009. **29**(31): p. 9875-87.
10. Finnegan, A., et al., *Single-Cell Transcriptomics Reveals Spatial and Temporal Turnover of Keratinocyte Differentiation Regulators.* Front Genet, 2019. **10**: p. 775.
11. Heydari, T., et al., *IQCELL: A platform for predicting the effect of gene perturbations on developmental trajectories using single-cell RNA-seq data.* PLoS Comput Biol, 2022. **18**(2): p. e1009907.
12. Garcia-Ojeda, M.E., et al., *GATA-3 promotes T-cell specification by repressing B-cell potential in pro-T cells in mice.* Blood, 2013. **121**(10): p. 1749-59.
13. Bao, X., et al., *Transcriptional Regulation of Early T-Lymphocyte Development in Thymus.* Front Immunol, 2022. **13**: p. 884569.
14. Carpenter, A.C. and R. Bosselut, *Decision checkpoints in the thymus.* Nat Immunol, 2010. **11**(8): p. 666-73.
15. Mingueneau, M., et al., *The transcriptional landscape of alphabeta T cell differentiation.* Nat Immunol, 2013. **14**(6): p. 619-32.
16. Fernandez-Garcia, J., et al., *CD8(+) T cell metabolic rewiring defined by scRNA-seq identifies a critical role of ASNS expression dynamics in T cell differentiation.* Cell Rep, 2022. **41**(7): p. 111639.
17. Collins, S., et al., *Cutting Edge: TCR-induced NAB2 enhances T cell function by coactivating IL-2 transcription.* J Immunol, 2006. **177**(12): p. 8301-5.
18. Intlekofer, A.M., et al., *Effector and memory CD8+ T cell fate coupled by T-bet and eomesodermin.* Nat Immunol, 2005. **6**(12): p. 1236-44.
19. Klein-Hessling, S., et al., *NFATc1 controls the cytotoxicity of CD8(+) T cells.* Nat Commun, 2017. **8**(1): p. 511.

20. Renoux, F., et al., *The AP1 Transcription Factor Fosl2 Promotes Systemic Autoimmunity and Inflammation by Repressing Treg Development.* Cell Rep, 2020. **31**(13): p. 107826.
21. Velasco Cardenas, R.M., et al., *Harnessing CD3 diversity to optimize CAR T cells.* Nat Immunol, 2023. **24**(12): p. 2135-2149.
22. Hanzelmann, S., R. Castelo, and J. Guinney, *GSVA: gene set variation analysis for microarray and RNA-seq data.* BMC Bioinformatics, 2013. **14**: p. 7.
23. Tsherniak, A., et al., *Defining a Cancer Dependency Map.* Cell, 2017. **170**(3): p. 564-576 e16.
